# Automated eDNA sampling for marine monitoring and biosecurity: optimising temporal resolution, remote deployments, and community engagement

**DOI:** 10.7717/peerj.21287

**Published:** 2026-05-28

**Authors:** Michelle Scriver, Ulla von Ammon, Suz Te Tai, Melissa Tipene, Krushil Watene, Nick Bamford, Austen C. Thomas, Anastasija Zaiko

**Affiliations:** 1 Sequench Ltd., Nelson, New Zealand; 2 Biosecurity Group, Cawthron Institute, Nelson, New Zealand; 3 Ngati Manu/Nga Kaitiaki o te Ahi, Kāretu, New Zealand; 4 Ngati Manu, Kāretu, New Zealand; 5 School of Humanities: Philosophy, University of Auckland, Auckland, New Zealand; 6 Northland Regional Council, Whangarei, New Zealand; 7 Smith-Root, Vancouver, WA, United States of America

**Keywords:** eDNA, Automated sampler, Metabarcoding, Biosecurity, Marine surveillance, Invasive species, Biodiversity monitoring, Temporal resolution, Robotic sampler, Community engagement

## Abstract

Environmental DNA (eDNA) offers unprecedented potential for monitoring high-risk coastal environments impacted by anthropogenic activities and is increasingly used for marine biosecurity applications. Yet, optimal temporal-spatial sampling strategies remain unresolved for dynamic coastal settings. Recent advances in automated eDNA sampling have allowed practitioners to improve temporal resolution, enabling simplified sampling methods and more adaptive sampling strategies. In this study, we evaluated whether higher temporal resolution improves biodiversity assessment and marine non-indigenous species (NIS) detection in a marina in Ōpua (Te Pēwhairangi (Bay of Islands), Aotearoa-New Zealand) using the recently introduced Smith-Root automated eDNA sampler. For this purpose, daily samples were collected over four weeks between 12:00–18:00 to assess eukaryotic and metazoan biodiversity using metabarcoding of the small ribosomal subunit RNA (18S rRNA) and mitochondrial *Cytochrome C Oxidase subunit* I (COI) genes. The results revealed that alpha diversity remained similar among weeks, but beta diversity shifted significantly, indicating that weekly replication captures meaningful ecological change in this setting. In parallel, NIS detections by screening the data with the Pest Alert Tool (PAT) comprised a mix of consistently present bivalves (*e.g.*, *Arcuatula senhousia*) and sporadically detected other fouling taxa (*e.g.*, *Botrylloides* spp.), illustrating how short-term (daily) temporal resolution modulates detection probability for intermittent targets. These patterns align with expectations for dynamic estuarine-coastal systems where behaviour, reproduction, and transport processes drive short-term variability. Comparison with prior single-day sampling at the same site suggests that a multi-week temporal design better resolves biodiversity compositional change. Operational refinements to the autosampler device (*e.g.*, optimised maintenance and temperature management) would further stabilise performance. Overall, automated high-frequency or repeated weekly eDNA sampling enhances biodiversity monitoring and biosecurity surveillance in coastal marinas by resolving temporal variability that governs detectability of rare taxa. Because these systems are low-infrastructure and portable, they are also suited for remote deployments and structured community-science use.

## Introduction

Environmental DNA (eDNA) continues to emerge as an effective, cost-efficient, and non-invasive tool for biodiversity monitoring and marine biosecurity, particularly in high-risk coastal environments where traditional methods are logistically challenging (Bowers et al., 2021; Jacquemot et al., 2024; Jarman et al., 2024; Lacoursiere-Roussel et al., 2018; Zaiko et al., 2018). Its value is especially evident in countries with extensive coastlines and limited surveillance resources (Carvalho et al., 2023), as it enables the generation of comprehensive biodiversity data at scales previously difficult to achieve (Deiner et al., 2017; Stat et al., 2017). As such, eDNA metabarcoding and other biomolecular technologies are increasingly being adopted as key tools to enhance ocean biomonitoring through global-scale collaborations and long-term research initiatives (*i.e.,* Ocean Biomolecular Observing Network (OBON), Ocean Biodiversity Information System (OBIS), and Marine Barcode of Life (MarBOL)). Improved biodiversity surveillance and monitoring are crucial for effectively managing the threats of marine non-indigenous species (NIS), which are intensifying due to anthropogenic activities (*e.g.*, shipping, aquaculture, and canal construction) and climate change (Blakeslee et al., 2020; Chan et al., 2019; Lieurance et al., 2025; Occhipinti-Ambrogi, 2007). As the uptake of eDNA-based surveillance accelerates globally, its value increasingly depends on sampling design: specifically, when, where, and how sampling is conducted, as well as the replication effort applied.

Traditional eDNA sampling methods can be labour-intensive and often require separate collection and filtration steps (Hendricks et al., 2023). To address the need for simplified and accessible collection methods for routine eDNA-based surveillance, especially for application by non-scientist end-users, a range of innovative approaches has emerged, including in-water filtration systems (Pochon et al., 2023; Von Ammon et al., 2023b), passive samplers (Von Ammon et al., 2025), and low-cost *in situ* devices (Maiello et al., 2023). Interest is also growing in eDNA sampling automation, which could address logistical barriers and enable flexible temporal designs, including daily, sub-daily, or repeated weekly series, while reducing handling steps and contamination risk, and even offer *in situ* analyses (George et al., 2024; Hansen et al., 2020; Hendricks et al., 2023; Nicholson et al., 2025; Yamahara et al., 2019). Critically, these systems allow temporal replication that can expose rare or transient signals missed by sparse sampling schedules. Recent work shows daily sampling captures rapid community dynamics, whereas coarser (*e.g.*, monthly) intervals yield the highest taxonomic richness (Sevellec et al., 2025); together, this highlights the importance of temporal resolution for enhanced biosurveillance sampling designs and early detections of NIS incursions (Sevellec et al., 2025). Automated devices, especially those with built-in preservation capabilities, enable these flexible sampling regimes tailored to specific biodiversity or biosecurity goals (Yamahara et al., 2019).

To explore the potential of automated eDNA sampling for coastal biosecurity, a small-scale pilot study was conducted in the Bay of Islands Marina in Ōpua, Te Pēwhairangi (Bay of Islands), Aotearoa-New Zealand, using a Smith-Root eDNA Autosampler (Smith-Root, USA). The marina is a key entry point for international yachts and recreational vessels, posing a risk for the introduction and spread of marine NIS (Acosta & Forrest, 2009; Floerl, Inglis & Hayden, 2005; Goldstien, Schiel & Gemmell, 2010). It is therefore one of 11 sites monitored under the National Marine High-Risk Site Surveillance (MHRSS) program (Woods et al., 2021a).

More importantly, the marine area associated with this pilot include sites of enduring mana (authority and responsibility) for the Māori community of Ngāti Manu and holds significant historical, cultural, and ecological value value (Hamilton, 2016). The coastal lands and waterways not only provide food and transport but remain sites of ancestral connection and ongoing socio-environmental practice (Paul-Burke et al., 2022; Te Tai, Peita & Watene, 2024; Watene, 2021). For Ngāti Manu, reconnecting community members, rebuilding knowledge, and pursuing customary practices in ways that nourish social and environmental wellbeing are vital, particularly in the face of climate-induced socio-environmental vulnerabilities. This engagement is especially significant given the growing kin-community diaspora, largely resulting from ongoing social and environmental injustices, affecting many Māori communities in the Far North and across Aotearoa-New Zealand. Therefore, a key aspect of the pilot was the recognition of and engagement with Ngāti Manu as partners in this project. This involved not only including the community in core deployment activities, such as sample collection and data interpretation, but ensuring that the pilot would support the community’s own socio-ecological needs and aspirations. This included understanding the importance of biodiversity loss on the coastal area as well as equipping the community with additional knowledge to support their own adaptation and mitigation strategies (Macinnis-Ng et al., 2024). Such collaboration efforts aim to strengthen relationships with Māori communities, not least through the (1) integration of Mātauranga Māori with modern science, and (2) recognition of the importance of this integration for biosecurity practices (Black et al., 2021; Wehi, Beggs & McAllister, 2019). It is within the context of these commitments that this project was believed to have the potential to resonate with and support the needs and aspirations of Ngāti Manu.

During the pilot study, daily eDNA samples were collected over a four-week period to test the benefits of increased temporal resolution for eDNA-based surveillance. The site location and sampling schedule were informed by a previous study (Scriver et al., 2024a), which examined temporal and spatial eDNA variation across tidal cycles in Ōpua using single-day point sampling at hourly intervals over a 12-hour tidal cycle. Although this does not allow direct comparison, using the same location provides a benchmark for contrasting extended temporal sampling using an autosampler with traditional single-day point sampling methods.

The study aimed to characterise detected eukaryotic community composition and examine patterns in putative marine NIS identified through data screening with the Pest Alert Tool (PAT; Zaiko et al., 2023). To achieve this, samples were analysed using a multi-marker metabarcoding approach targeting the V4 region of the nuclear small subunit ribosomal RNA (18S rRNA) gene, optimised for marine microbial eukaryotes such as protists (Guillou et al., 2013), and the mitochondrial *Cytochrome C Oxidase subunit I* (COI) gene, with primers designed for marine invertebrate communities (Leray et al., 2013). This dual-marker approach has been shown to improve detection of marine NIS and provide a more comprehensive assessment of eukaryotic biodiversity across taxonomic levels, thereby improving diversity estimates (Borrell et al., 2017; Borrell et al., 2018; Holman et al., 2019; Pearman et al., 2020; Scriver et al., 2024a; Von Ammon et al., 2018; Von Ammon et al., 2023a). The integration of COI and 18S rRNA is widely recommended for capturing overall eukaryotic and metazoan biodiversity (Tagliabue et al., 2025), as different markers can differ in taxonomic resolution, PCR amplification bias, taxonomic coverage, and reference database completeness (Hestetun et al., 2020; Stefanni et al., 2018; Zhang et al., 2018).

The study addressed the following objectives:

1. quantify week-scale changes in detected eukaryotic communities;
2. evaluate the effect of multi-week temporal coverage on Aotearoa-New Zealand’s marine NIS detection
3. assess the usability of the automated eDNA sampling approach by local community stakeholders.

By combining fine-scale temporal resolution with Māori partnership, this study aims to contribute to optimised eDNA sampling protocols for routine biosecurity monitoring and improved early detection of marine NIS in high-risk coastal environments.

## Materials & Methods

### Sample collection

**Figure 1:**
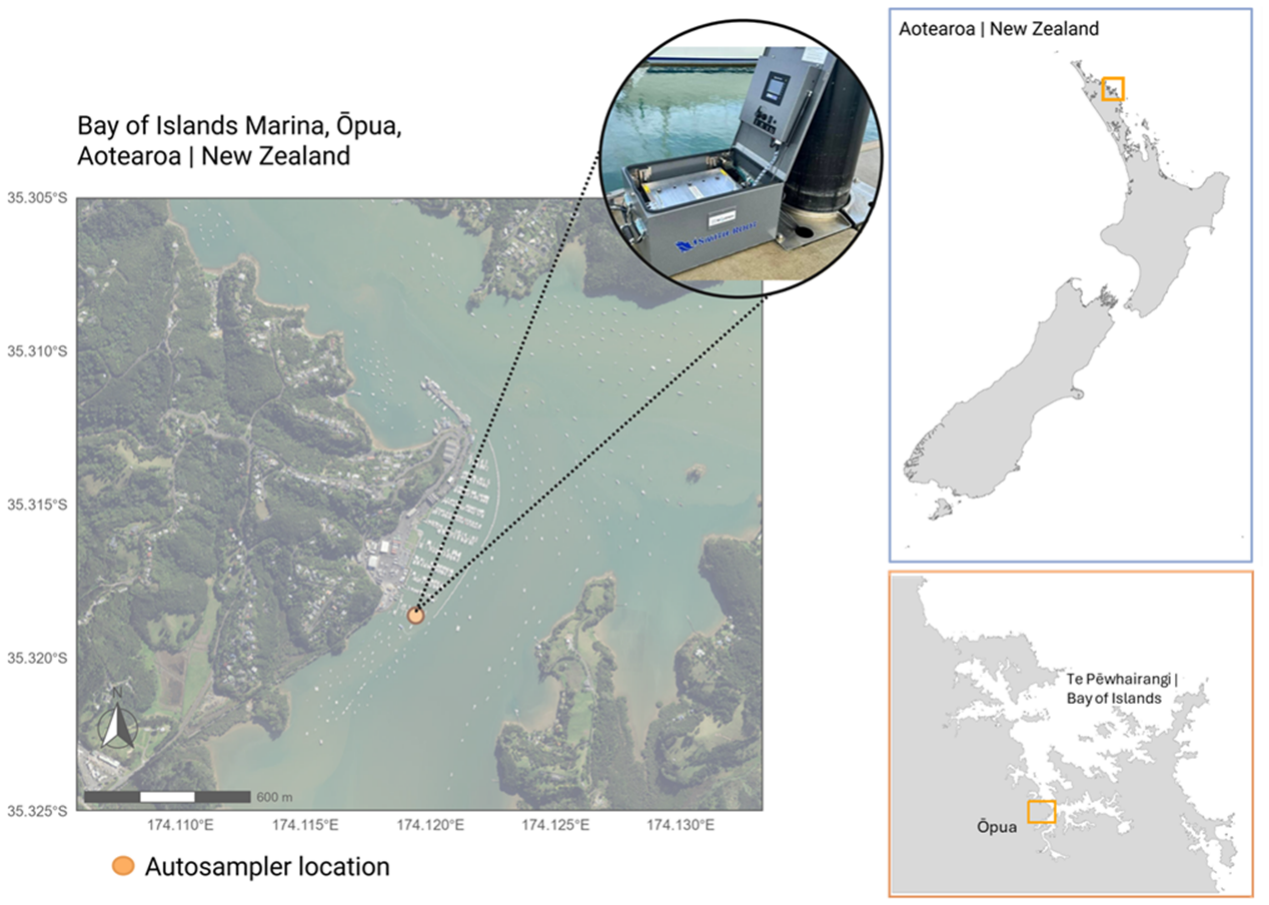
Sample map of Bay of Islands Marina with eDNA Autosampler location, Aotearoa-New Zealand. Map showing the eDNA Autosampler deployment site in the Bay of Islands Marina, Ōpua, Aotearoa-New Zealand. The orange dot indicates the location of the Smith-Root eDNA Autosampler (Smith-Root, USA). The inset maps on the right show the position of Te Pēwhairangi (Bay of Islands) and Ōpua within the wider geographic context of Aotearoa-New Zealand. The overview maps were generated in R using the ggplot2 and sf packages with vector-based basemap layers sourced from the NZ Coastlines and Islands Polygons Topo 1:50,000 dataset (Land Information New Zealand, LINZ). The marina-scale basemap imagery was derived from World Imagery, Firefly (Esri, Maxar, Earthstar Geographics, and the GIS User Community), with the sampling location and autosampler symbol added by the authors. This figure was adapted from Scriver et al. (2024a), licensed under Creative Commons Attribution 4.0 (CC BY 4.0). Changes include the use of alternative basemap imagery and the addition of the autosampler deployment location.

**Figure 2:**
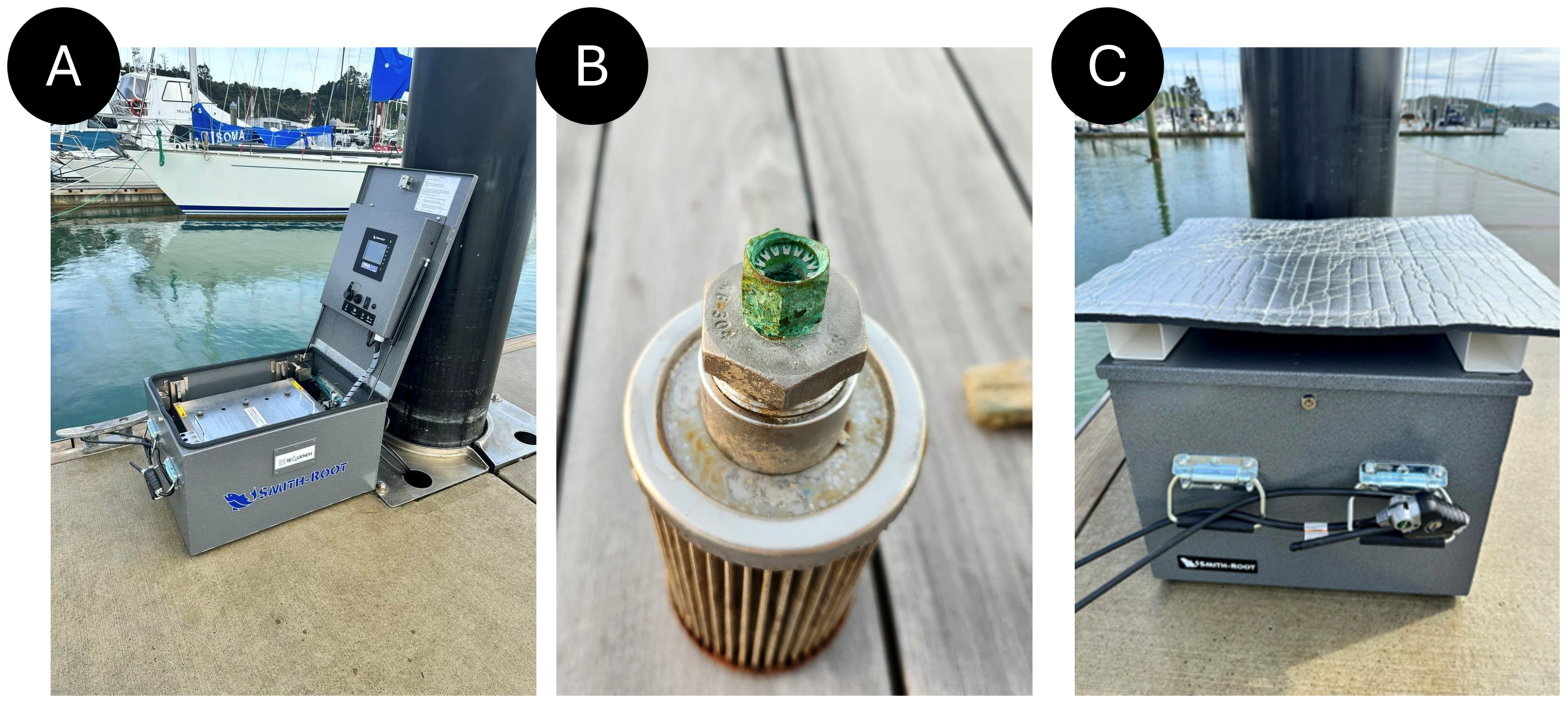
Smith-Root eDNA Autosampler deployment. Photos depicting the deployment of the Smith-Root eDNA Autosampler in the Bay of Islands Marina (A), carried out in collaboration with the local Ngāti Manu hapū. The deployment included a community engagement and training session with Melissa- the local volunteer, and the autosampler was fitted with a 2.5-meter intake hose equipped with a mesh strainer to minimize clogging (B), as well as thermal insulation to protect the unit during operation (C).

A vacuum filtration-based automated eDNA sampler (eDNA Autosampler, Smith-Root, Vancouver, WA, USA) was deployed at the pier located at the end of Ōpua Harbour, Te Pēwhairangi (Bay of Islands), Aotearoa-New Zealand (35.31861°S, 174.11971°E) (Fig. 1) from 2 December 2023 to 4 January 2024, in collaboration with Ngāti Manu community (Fig. 2). This specific marina site corresponded to one of the six locations previously monitored by Scriver et al. (2024a).

All samples are collected under Special Permit (SP822-2) issued to the Cawthron Institute by the New Zealand Ministry for Primary Industries, which permits aquatic sample collection for investigative research.

The autosampler was equipped with thermal protection (Fig. 2C), and a 2.5-meter intake hose fitted with a stainless-steel strainer at the end of the pipe to minimise clogging (Fig. 2B). In addition, two temperature loggers (HOBO^®^, Australia) were deployed: one inside the autosampler and one attached to the intake pipe to monitor internal sampler temperature and water temperature during deployment. Before the deployment, the system was cleaned by pumping through 10L of 5% bleach solution and then flushing it for 5 min with distilled water, following the manufacturer’s recommendations. No bleach cleaning was performed between weekly sampling periods. The autosampler was programmed *via* its onboard graphical user interface to autonomously collect eight daily eDNA samples using Smith-Root’s self-preserving 47 mm, 5 µM polyethersulfone filters (PES; Smith-Root, USA). The programmable settings were as follows: target volume 4 L, target flow 1.0 L/min, flush volume 2 L, and maximum pressure 10.0 PSI. The field team followed the provided detailed sample collection, system settings and autosampler maintenance protocol (Supplementary Data, Appendix 1 & Appendix 2). Sampling occurred between 12:00 and 18:00 over the 34-day period.

Throughout the deployment, a local volunteer, Melissa Tipene from the Ngāti Manu, took charge of the work. Melissa’s involvement stemmed from their strong interest and pursuit of further education in marine science, as well as their desire to be actively engaged in its care. Through this role, they contributed not only to scientific and cultural knowledge but also to the development of their leadership skills, and sense of ownership in the stewardship of their community’s waters. As part of the deployment, every eight days Melissa retrieved the used self-preserving filters, placing the entire housing back into its resealable bag for storage. The eDNA filter housing is made of biodegradable, hydrophilic plastic, which preserves the DNA through desiccation by absorbing remaining moisture, preventing degradation at ambient field temperatures (Thomas et al., 2019). A new set of filters was then installed for the next cycle of automated sampling. All collected filter housings were stored in their resealable bags in a dry, dark environment at room temperature until transport to the Cawthron Institute (Whakatū/Nelson, Aotearoa-New Zealand), as recommended by the manufacturer. Melissa was also responsible for routine maintenance of the sampler, including battery checks, operational assessments, taking control samples as needed, and cleaning of the intake strainer at every exchange cycle.

Control field samples were taken once a week by opening the Smith-Root filter packaging, briefly exposing the filter to the air while mimicking the field manipulations, before sealing the filter back into the manufacturer’s packaging. Additionally, post-deployment control was collected at the end of the experiment following system cleaning, by running distilled water through the intake line and a Smith-Root filter. All field samples were handled and treated the same way as biological samples.

### DNA extractions and amplicon PCR

To prevent cross-contamination, each stage of the molecular workflow, including DNA extraction, PCR setup, template addition, and amplicon PCR clean-up, was conducted in a separate sterile laboratory designated for that specific step. All laboratories were equipped with UV sterilization units, which were activated for at least 15 min before and after each use. PCR setup, template addition, and amplicon PCR clean-up were performed within laminar flow cabinets fitted with HEPA filtration. Throughout all procedures, aerosol-barrier tips (Axygen BioScience, USA) were used to ensure sample integrity.

In the laboratory, Smith-Root filter housing units were opened, and the filters were placed into PowerBead Pro Tubes from the DNeasy PowerSoil Pro Kit (Qiagen, Germany) with 800 µl of Solution CD1. The samples were homogenised *via* bead beating for two minutes (1600 MiniG Spex SamplePrep, United States) and then centrifuged at 10,000 × g for five minutes at 20 °C using an Eppendorf Centrifuge 5430R (Eppendorf, Germany). The supernatant from each homogenised sample was transferred individually into a clean 2 ml microcentrifuge tube and processed using the DNeasy PowerSoil Pro kits (Qiagen, Hilden, Germany) on a QIAcube extraction robot, following the manufacturer’s protocol. DNA extractions were eluted in 100 µl aliquots, with DNA extraction blanks (negative controls) included during each extraction series.

Metabarcoding targeted the mitochondrial COI gene (313 bp) for metazoans (Leray et al., 2013) and the V4 region of the nuclear small ribosomal subunit RNA (18S rRNA) gene (500 bp) for broad eukaryotic diversity (Zhang et al., 2018). These markers were selected to encompass diverse taxonomic groups within the sampled marine communities (Brandt et al., 2020; Leite et al., 2021). COI and 18S rRNA primers included Illumina overhang adaptors following Kozich et al. (2013). PCR amplification used an Eppendorf Mastercycler (Eppendorf, Germany), with 50 µL reactions containing 25 µL MyFi Mix (Bioline, UK), 1 µL of each 10 µM primer, 20 µL DNA-free water, and 3 µL template DNA. Cycling conditions were: initial denaturation at 95 °C for 2 min, followed by 39 cycles of 95 °C for 20 s, 52 °C for 20 s, 72 °C for 20 s, and a final extension of 72 °C for 10 min. PCR was performed with a single PCR replicate per sample.

Negative controls without template DNA were included during PCRs. The amplification products were purified and normalised using the SequalPrep Normalization Kit (Thermo Fisher Scientific, Waltham, MA, USA) to ∼1 ng/µL.

### High-throughput sequencing

The purified amplicons were sent to Sequench Ltd (Whakatū/Nelson, Aotearoa–New Zealand) for indexing using the Nextera XT kit (Illumina, San Diego, CA, USA) and sequenced on an Illumina MiSeq platform. To monitor potential contamination during library preparation, a water blank was included at the indexing step as a sequencing control. The amplicons used for indexing included 21 environmental samples and eight controls (four field controls, one post-deployment control, one extraction blank, one negative PCR control, and one sequencing control). Each sample and control was amplified for two genetic markers (18S rRNA and COI), resulting in 42 environmental amplicons and 16 control amplicons for indexing. The environmental samples were collected once daily and grouped to generate temporal replicates, defined as week 1 (02–10 December 2023), week 3 (19–21 December 2023), and week 4 (26 December 2023–04 January 2024). Due to technical issues, including battery failure and handling errors such as improper insertion, no samples were collected during week 2 (11–16 December 2023), and only three samples were obtained in week 3, compared with nine samples in weeks 1 and 4.

Following indexing, the indexed libraries were pooled and cleaned using a 1.1 × ratio of AMPure XP beads (Beckman Coulter, Brea, CA, USA). The pooled libraries were then quality-checked and quantified using a Qubit Fluorometer (Thermo Fisher Scientific, Waltham, MA, USA) and a Bioanalyzer with the Agilent DNA 1000 Kit (Agilent Technologies, Santa Clara, CA, USA). The final library pool was adjusted to a loading concentration of 6 pM, with a 15% PhiX spike, before sequencing using the MiSeq Reagent Kit v3 (600-cycle, 2  × 301 bp) (Illumina, San Diego, CA, USA). Raw sequence reads have been submitted to the NCBI Short Read Archive under accession number PRJNA1332291.

### Bioinformatics

Bioinformatic pipelines for assigning amplicon sequence variants (ASVs) for both 18S rRNA and COI sequence data were identical unless otherwise stated. The pipeline is outline briefly below, for more details see Von Ammon et al. (2023b).

Primer sequences were removed using Cutadapt (Martin, 2011) allowing one mismatch. Sequences were then processed in R (R Core Team, 2025) using the ‘DADA2’ package (Callahan et al., 2016). Reads were truncated to 225 and 216 bp for 18S rRNA and COI forward and reverse reads, respectively, and filtered with a default maximum number of “expected errors” (maxEE) of 2. Reads not meeting this threshold were discarded. A parametric error matrix was constructed, and sequence variants for the forward and reverse reads were dereplicated. Singletons were removed, paired reads merged (min 10 bp overlap, no mismatches), and chimeras filtered using removeBimeraDenovo.

For the taxonomic classification of 18S rRNA gene amplicons, ASVs were matched against the PR2 database (Guillou et al., 2013). Taxonomic assignments were normalised with the taxa_normalization function in the R package “biohelper” (v0.0.18.0; Laroche, 2023), which standardises eukaryotic names derived from assignTaxonomy function from “DADA2” to NCBI-curated classification. For the COI gene dataset, taxonomic assignment was performed as described in Laroche (2023) using “biohelper” (Laroche, 2023). This method aims to reduce the number of unassigned sequences and increase taxonomic resolution using megablast from Blastn application; and Blastn on the entire GenBank nucleotide (nt) database. The results were combined into a phyloseq object using R package “phyloseq“ (version 1.52.0; McMurdie & Holmes, 2013).

Contaminants were removed using max_v option from “biohelper” package. This approach sets the maximum read count observed across all control types (field blank, extraction blank, PCR blank, and sequencing blank) as the threshold for identifying potentially contaminating ASVs (Bell et al., 2019). ASVs below this threshold in environmental samples were excluded; counts above the threshold were adjusted by subtracting the threshold from their read count. The resulting cleaned phyloseq object was saved, and any taxa assigned to bacteria or with unassigned or unknown taxonomy were removed from the dataset, these final datasets are referred to as the cleaned dataset.

### Statistical analysis

All statistical analyses were performed in R (version 4.4.1; R Core Team, 2025). Rare ASVs with a total abundance (read count) of fewer than five across all samples were removed from the 18S rRNA and COI dataset to improve the analysis and interpretation of community dynamics by enhancing comparability and reducing artifacts and noise (De Santiago et al., 2021; Deng, Umbach & Neufeld, 2024; Zaiko et al., 2022). These datasets with the rare ASVs removed are referred to as the filtered dataset. Rarefaction curves were generated to assess sequencing depth adequacy for biodiversity analyses.

For alpha and beta diversity analyses, read counts were rarefied to standardise sequencing depth across samples using the R packages “vegan” (version 2.7.1; Dixon, 2003; Oksanen, 2020) and “ggplot2” (version 3.5.2; Wickham, 2016). The 18S rRNA gene untransformed filtered dataset was rarefied to 13,000 reads per sample and the COI untransformed filtered dataset to 14,790 reads per sample. These rarefied datasets were used for all diversity metrics and community composition analyses, which were retained at the ASVs level. Alpha diversity (observed ASVs) was calculated using “microeco” (version 1.15.0; Liu et al., 2021), with differences between weeks tested by Kruskal–Wallis and Dunn’s multiple comparisons. Beta diversity was analysed with “microeco” using PERMANOVA and PERMDISP on Hellinger-transformed data and Bray-Curtis dissimilarity (Laporte et al., 2021). Community composition differences across weeks were visualised *via* Principal Coordinates Analysis (PCoA) using the R package “microViz” (version 0.12.7; Barnett, Arts & Penders, 2021).

For general community visualisation, including taxonomic summaries, the rarefied datasets were converted to relative read abundance, calculated as the proportion of reads per ASVs relative to the total reads within each sample (ASV read count divided by total sample read count) and displayed as barplots using the R packages “ggplot2” and “phyloseq”. Differential abundance analysis was also performed on the rarefied datasets to identify significant class-level taxa between sampling weeks. This was done using the *trans_diff* function from the R package “microeco”, applying Dunn’s Kruskal–Wallis multiple comparisons. The resulting data were plotted with “microeco”, with values displayed as relative read abundance.

The relationship between temperature inside the autosampler and detected alpha diversity (observed ASVs) for 18S rRNA and COI was visualised using scatterplots with LOESS smoothing to capture non-linear trends, and linear regression with 95% confidence intervals to assess linear effects. Pearson correlation tests were performed to quantify linear associations. To account for potential non-linear responses, a quadratic model was also fitted with observed ASVs as the response variable and temperature and its square as predictors. All analyses and visualisations were conducted in R using “ggplot2” and “dplyr” and clean datasets, *i.e.,* after decontamination, were used.

### Assessing detection of marine non-indigenous species

The Pest Alert Tool (PAT) (https://pest-alert-tool-prod.azurewebsites.net/), developed by the Marine Biosecurity Toolbox Programme at the Cawthron Institute (Zaiko et al., 2023), was used to screen metabarcoding-derived FASTA files for eDNA signals of NIS in Aotearoa-New Zealand. The decontaminated FASTQ files—the cleaned datasets, not rarefied and without filtering for rare species—were consolidated into a single FASTQ file and uploaded to the PAT for analysis of 18S rRNA (submitted 1,265 ASVs) and COI gene sequences (submitted 1,059 ASVs), with parameters set to a minimum sequence identity match of 100% and a minimum sequence length of 310 bp for COI and 400 bp for 18S rRNA. Putative pest matches were verified through reference sequence comparisons and phylogenetic tree generation using BLAST pairwise alignment in the NCBI database. A robust phylogenetic match was defined, based on the phylogenetic tree, as a taxon grouping on a branch with the same species and clearly separated from other species, as suggested by Zaiko et al. (2023). Species that did not meet this criterion were initially classified at the genus level (*i.e.,* multiple species from the same genus appearing on a single branch). If genus-level placement remained ambiguous, such as when multiple genera clustered on the same branch, these taxa were removed from the analysis. The verified list of putative pests was then used to filter the 18S rRNA and COI gene datasets. Contamination was addressed using the maximum sequence count for each ASVs threshold, as described above, as this method mitigates potential contamination without limiting the detection of rare NIS (Pearman et al., 2020). To account for sequencing depth variability, relative read abundance was calculated using “microeco” for the NIS eDNA metabarcoding data, rather than rarefaction, to avoid data loss and ensure all detections were retained, as detection was the primary goal (Laporte et al., 2021). This methodological framework aligns with what was done by Von Ammon et al. (2023a) and Scriver et al. (2024a), who also employed the PAT for metabarcoding screenings in Ōpua.

To assess the relationship between marine NIS detection and autosampler temperature, the 18S rRNA and COI filtered marine NIS ASVs tables were first converted to presence–absence format, and the number of NIS detected per sample was calculated as the sum of taxa with non-zero counts. The relationship was explored using scatterplots with linear regression smoothing for each marker. Statistical analysis was performed using Poisson generalised linear models (GLMs), with autosampler temperature and sampling week as predictors. Model fit and assumptions were evaluated *via* residual plots, normal Q–Q plots, and Cook’s distance. Data manipulation and cleaning were performed using “dplyr” and “phyloseq”, and the GLMs were fitted using base R.

When comparing data from Scriver et al. (2024a), which employed traditional point eDNA sampling methods, the detected marine NIS data were filtered to include only samples from Station D, ensuring consistency with the sampling location of the present study. Comparisons between traditional point eDNA sampling methods and automated sampling were visualised using Venn diagrams with the R package “eulerr” (version 7.02; Larsson & Gustafsson, 2018). Taxonomy was agglomerated to the species level prior to visualization, and silhouette icons of detected NIS were sourced from PhyloPic (https://www.phylopic.org/).

## Results

In total, 21 samples were successfully collected over the autosampler deployment period. Sampling volumes ranged from 0.4 to 2.6 litres, with an average of approximately 1.5 litres, with four of the 21 samples reporting 0 L volume filtered.

The seawater temperature at the sampling site ranged between 19.0 and 25.8 °C, demonstrating diurnal fluctuation patterns (Fig. S1). The temperature inside the autosampler box ranged between 12.0 and 39.5 °C, fluctuating substantially during the daily cycles, with peak values observed between 15 and 18 h (Fig. S2).

### Biodiversity overview

Overall, data rarefaction curves indicated that sequencing depth was adequate, as all sample diversity curves plateaued (Fig. S3). After contamination removal, there were 511,160 sequence reads and 1,265 ASVs for the 18S rRNA gene, and 770,189 sequence reads and 1,059 ASVs for the COI gene. Following the removal of sequencing reads < 5, the filtered dataset was reduced to 510,847 reads and 1,153 ASVs for 18S rRNA, and 770,036 reads and 1,008 ASVs for COI. After rarefying to 13,000 reads per sample for 18S rRNA, the rarefied dataset included 234,000 reads and 1,126 ASVs, with two samples removed for falling below the rarefaction threshold and 27 ASVs were excluded. For COI, rarefying to 14,790 reads per sample resulted in a rarefied dataset with 251,430 total reads and 996 ASVs, with two samples excluded and 12 ASVs removed. Taxonomic resolution for 18S rRNA spanned 5 kingdoms, 32 phyla, 69 classes, 134 orders, 191 families, 228 genera and 142 species. For COI, 3 kingdoms, 18 phyla, 33 classes, 54 orders, 59 families, 51 genera, and 53 species were detected.

**Figure 3:**
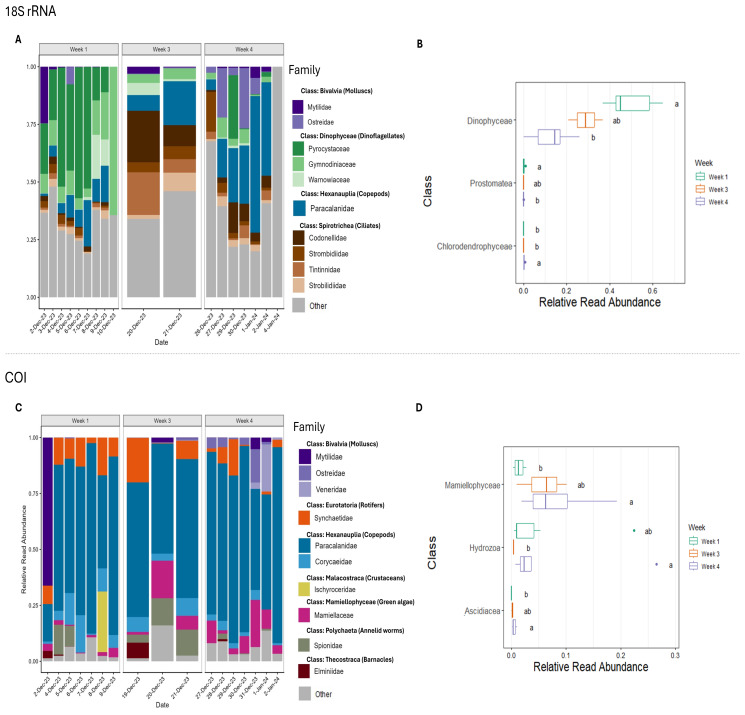
Taxonomic composition and differential abundance by sampling week. Plots show the top 10 families by relative read abundance for the (A) small ribosomal subunit RNA (18S rRNA) and (C) mitochondrial *Cytochrome C Oxidase subunit I* (COI) gene datasets, displayed by sampling date and faceted by sampling week. Classes showing significant differences in relative read abundance across weeks are presented in panels (B) for 18S rRNA and (D) for COI. Significance is indicated by letters (a, b, ab), where taxa sharing a letter are not significantly different, and taxa with different letters differ significantly. Families not within the top 10 are labeled in grey (“Other”). Note that “week 2” was not sampled due to a malfunctioned battery.

Taxonomic composition varied across sampling weeks, with distinct patterns at both the class and family levels (Fig. 3). In the 18S rRNA dataset, Dinophyceae (Dinoflagellates) dominated relative read abundance in week 1 (mean = 49.9%) but declined in weeks 3 (mean = 28.9%) and 4 (mean = 12.6%), with a significant difference between weeks 1 and 4 (Figs. 3A & 3B). In week 4, Hexanauplia, particularly the family Paracalanidae (calanoid copepods), accounted for 25.8% of reads, with abundance increasing over time (Fig. 3A). Other class-level taxa, including Prostomatea (ciliates) and Chlorodendrophyceae (green algae), also showed significant changes, with Prostomatea decreasing from 0.297% to 0.002% and Chlorodendrophyceae increasing slightly from 0.053% to 0.316% from week 1 to week 4, although both taxa remained at low relative abundance (<1%) across all weeks (Fig. 3B). In the COI dataset, Hexanauplia dominated all weeks (mean relative read abundance: week 1 = 50.1%, week 3 = 46.6%, week 4 = 53.9%), with Paracalanidae contributing substantially, and the top ten families were more evenly distributed across classes compared to 18S rRNA (Figs. 3C & 3D). Among notable changes in mean relative read abundance, Mamiellophyceae (green algae), particularly the family Mamiellaceae, increased over the sampling period (week 1 = 1.41%, week 3 = 5.85%, week 4 = 7.96%; Figs. 3C & 3D). Additionally, class-level taxa outside the top ten families, including Hydrozoa (week 1 = 4.83%, week 3 = 0.376%, week 4 = 5.72%) and Ascidiacea (week 1 = 0.0164%, week 3 = 0.167%, week 4 = 0.483%), showed a decrease in relative read abundance in week 3 followed by a significant increase from week 1 to week 4, respectively (Fig. 3D).

When assessing overall alpha diversity, some fluctuations in observed ASVs were noted between sampling weeks (Fig. S4). However, there were no statistically significant differences in observed ASVs across weeks for either the 18S rRNA or COI gene (Kruskal–Wallis Rank Sum Test: *p* = 0.199 for 18S rRNA; *p* = 0.926 for COI).

**Figure 4:**
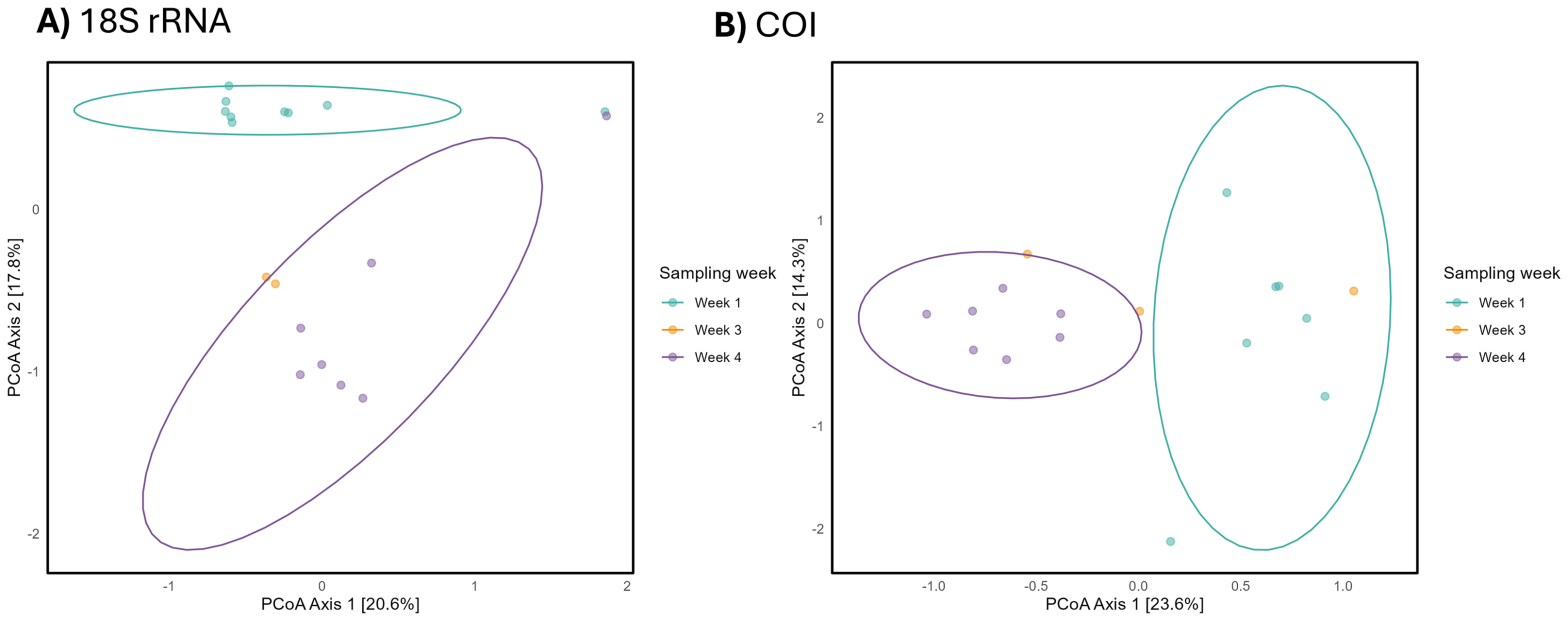
PCoA of eDNA community composition across sampling weeks. Principal coordinates analyses (PCoA) of environmental DNA (eDNA)-derived community composition across sampling weeks for the (A) small ribosomal subunit RNA (18S rRNA) and (B) mitochondrial *Cytochrome C Oxidase subunit I* (COI) gene datasets. The analyses were conducted using Bray–Curtis dissimilarity distances after a Hellinger transformation in R. Note that “week 2” was not sampled due to malfunctioned battery.

In contrast, when examining beta diversity using Bray-Curtis dissimilarities, PERMANOVA analysis revealed significant differences in beta diversity across weeks for both markers (*p* = 0.007 for 18S rRNA; *p* = 0.003 for COI) (Table S1). The analysis indicated that sampling week explained approximately 22.2% of the variation in community composition for 18S rRNA and 20.9% for COI. A PERMDISP (beta dispersion) analysis found no significant differences in dispersion across weeks (*p* = 0.165 for 18S rRNA; *p* = 0.528 for COI) (Table S1). To visualise differences in beta-diversity, Principal Coordinates Analysis (PCoA) was performed, revealing clear separation between week 1 and week 4 samples (Fig. 4). Interpretation of the week 3 sample grouping was limited due to the low number of samples recovered (*n* = 2 for 18S rRNA; *n* = 3 for COI).

Finally, to assess the effect of temperature fluctuations on eDNA integrity and recovered biodiversity, correlations between internal autosampler temperature and the number of ASVs were examined. Although both datasets showed a week negative correlation with temperature (Pearson’s r = −0.262 for 18S rRNA; r = −0.271 for COI), no significant effect on ASVs richness was found for either marker: 18S rRNA (*p* = 0.265) or COI (*p* = 0.261) (Fig. S5).

### Marine non-indigenous species detection

A key aim of this study was to assess the potential of extended temporal eDNA sampling design for enhanced detection of marine NIS, by screening two metabarcoding markers (18S rRNA and COI) datasets for known Aotearoa-New Zealand NIS using the Pest Alert Tool. Detection varied across sampling weeks and the dual-marker approach provided complementary coverage of the community, allowing detection across multiple taxonomic groups and levels.

**Figure 5:**
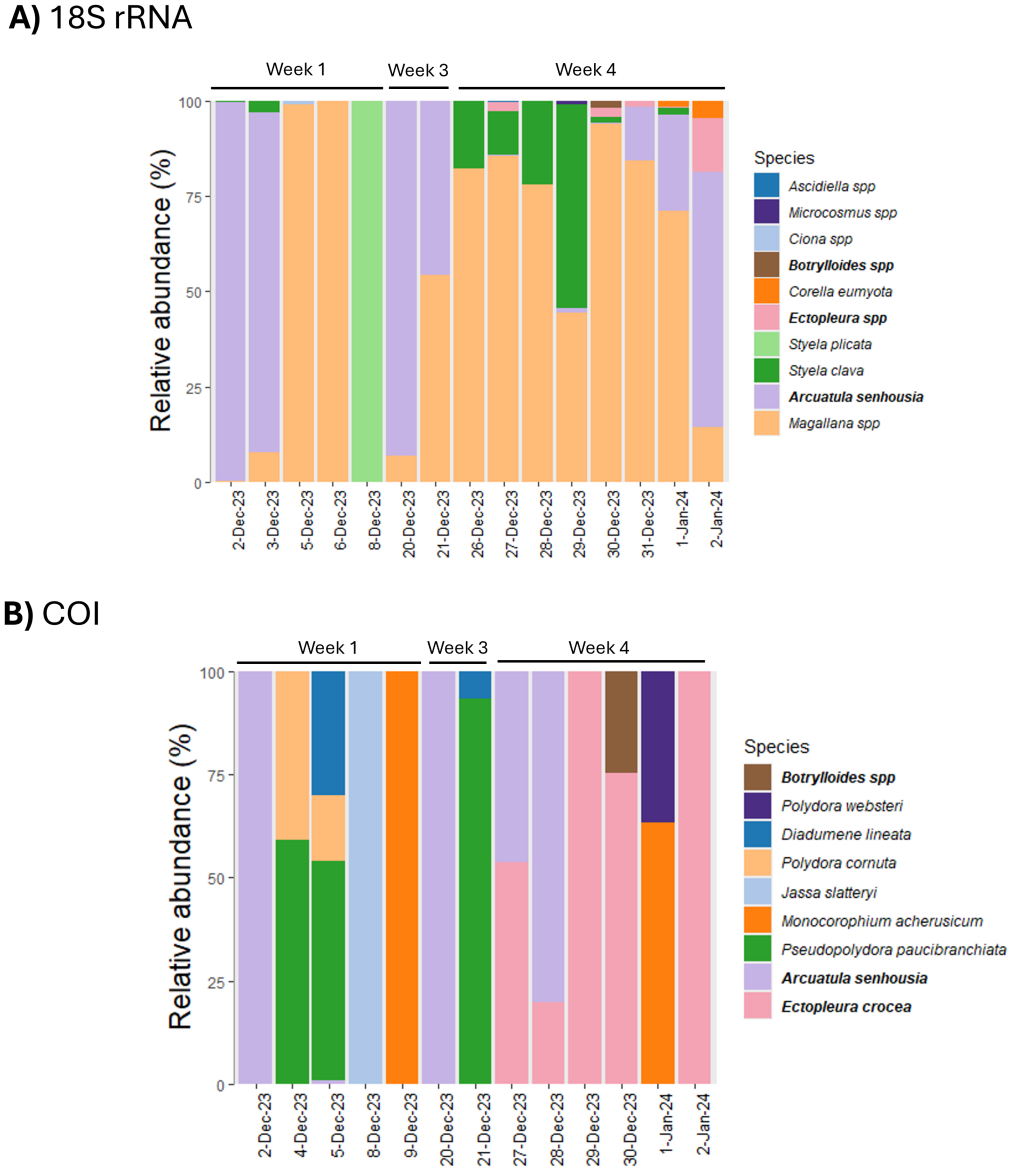
Relative abundance of marine NIS. Bar chart depicting the relative read abundance of detected marine non-indigenous species (NIS) from the (A) small ribosomal subunit RNA (18S rRNA) and (B) mitochondrial *Cytochrome C Oxidase subunit I* (COI) gene datasets by sampling date. Datasets were filtered for detected NIS, aggregated to species level, and transformed to relative abundance. Bold taxa in the legend indicates marine NIS detected in both gene datasets.

In total, ten taxa were identified in the 18S rRNA dataset (four at the species level, six at the genus level), and nine taxa in the COI dataset (eight at the species level, one at the genus level) (Figs. 5A & 5B). One species initially identified by PAT in the COI dataset, *Celleporaria nodulosa*, was removed because its phylogenetic placement was unresolved even at the genus level and therefore could not be confirmed. Three taxa were shared between both markers: *Arcuatula senhousia* (Asian date mussel), *Ectopleura* spp. (tubular hydroids; 18S rRNA)/*Ectopleura crocea* (pink-mouthed hydroid; COI), and *Botrylloides* spp. (colonial ascidian) (Fig. 5).

The 18S rRNA dataset mainly captured sessile biofouling invertebrates in the phyla Mollusca and Chordata, particularly tunicates (seven of 10) (*Styela plicata*, *Styela clava*, *Microcosmus* spp., *Ascidiella* spp., *Ciona* spp., *Corella eumyota*, and *Botrylloides* spp.) and bivalves (*Magallana* spp. and *A. senhousia*). In contrast, the COI marker more commonly detected benthic invertebrates in the phyla Arthropoda and Annelida, including polychaete worms (*Pseudopolydora paucibranchiata*, *Polydora websteri*, *Polydora cornuta*) and amphipods (*Monocorophium acherusicum*, *Jassa slatteryi*).

Patterns of detection consistency varied. Bivalves like *Magallana* spp. and *A. senhousia* were consistently detected across all sampling weeks, often with high relative read abundance (>40%) (Fig. 5). In contrast, most other species showed sporadic detection, typically appearing in only one or two sampling weeks. Some species, such as *Corella eumyota* and *Botrylloides* spp. in the 18S rRNA dataset, had sporadic low relative read abundance, whereas others, like *Styela plicata* (18S rRNA) and *Jassa slatteryi* (COI) showed high relative read abundance, but only during a single sampling period (Fig. 5).

When examining the influence of sampling week and internal autosampler temperature on marine NIS detection, no significant effect was observed in the COI dataset. However, for 18S rRNA, significantly more NIS were detected in week 4 compared to week 1 (*p* =0.00136) (Tables S2 & S3). Temperature showed a slight, non-significant negative association on NIS detection for 18S rRNA and COI (18S: Estimate = −0.0373, *p* = 0.252; COI: Estimate = −0.0515, *p* = 0.261).

### User’s observations regarding the autosampler areas application in coastal marina settings

Another important consideration for automated eDNA sample collection is ease of use (especially for applications by non-specialist users) and operational performance under different field settings and environmental conditions.

In our study, the autosampler device was initially set up in the field through collaboration between experienced molecular field scientists and community members. It was then operated and maintained throughout the experiment independently by local communities with limited prior experience with this technology. Overall, the device functioned well, and no major issues or difficulties were reported by the operators, except for the unexpected failure of an external battery, which led to the loss of one week’s worth of samples. Several samples were also not collected, with the instrument reporting 0 L filtered volume. These sample failures occurred randomly across the experimental timeline and were most likely caused by incorrectly inserted filters or clogged intake hoses.

Lastly, one important observation, especially relevant for saltwater deployments, was the biofouling on the intake hose and rust on the mesh strainer (Fig. S6). Although the mesh strainer was cleaned weekly, the intake hose itself was not, highlighting the need for regular maintenance of the hose and selection of materials suitable for marine environments.

## Discussion

In this study, the Smith-Root eDNA Autosampler was successfully deployed for the first time in a coastal marine environment to detect marine NIS and monitor biodiversity trends relevant to local environmental management and of value to the local hapū (a kinship-based sub-group of the Māori community with shared ancestry and traditional connections to the area). Using a dual-marker metabarcoding approach, biodiversity and NIS detection were characterised over an extended temporal period, revealing distinct patterns and enhancing detection capability.

### Observed temporal detection patterns and implications for sampling design

While ASVs richness appeared relatively stable across weeks, significant shifts in beta diversity indicate temporally dynamic communities. The complementary use of 18S rRNA and COI markers provided a more comprehensive overview of these patterns and, consistent with the literature, produced comparable results and similar trends in community composition analyse (Tagliabue et al., 2025). Both markers consistently showed dominance of Hexanauplia (copepods), in agreement with prior studies reporting high 18S rRNA and COI relative read abundances for copepods (Banerji et al., 2018; Gunther et al., 2018; Zhao et al., 2021). The COI dataset also revealed significant increases in Mamiellophyceae (green algae) over the sampling period. In the 18S rRNA dataset, Dinophyceae initially dominated relative read abundance, whereas Hexanauplia increased in relative read abundance by Week 4. This pattern is consistent with plankton studies in which copepods are consistently detected, while protist taxa such as dinoflagellates exhibit temporally distinct relative sequence abundance patterns compared to arthropods (Banerji et al., 2018).

Comparing relative abundance changes within and between datasets can reveal potential community shifts. For example, declines in relative read abundance of Dinophyceae in 18S rRNA dataset could be associated with increases in Prostomatea (ciliates) and green algae (18S rRNA: Chlorodendrophyceae; COI: Mamiellophyceae), suggesting a phytoplankton community shift toward smaller, fast-growing autotrophs (Avrahami et al., 2025) and active heterotrophic microbial consumers (Calbet, 2025). Similar studies examining plankton responses to environmental variables over time have shown that combined 18S rRNA and COI metabarcoding can detect significant temporal transitions in zooplankton communities (Banerji et al., 2018; Hall et al., 2025; Zhao et al., 2021). In a 24-hour study at the same site, Scriver et al. (2024a) observed fine-scale temporal shifts in community composition, with dinoflagellates and various algal groups fluctuating across tidal cycles. These detected temporal patterns likely reflect biological factors (behaviour, mobility, reproduction, predation, diurnal migration) (Dowell et al., 2024; Sevellec et al., 2025), eDNA detectability, decay, and shedding rates (Wood et al., 2020), as well as environmental drivers such as stratification, currents, wind, nutrient availability, grazing pressure, salinity, and temperature fluctuations (Stewart, 2019; Banerji et al., 2018; Kong et al., 2022; Pearman et al., 2016). Temporal variation in detected eDNA may also be influenced by the accumulation and succession of biofilms and biofouling on submerged sampling equipment, such as intake hoses, which can alter local community composition and lead to partial clogging of pipes, thereby affecting detection patterns (Alexander et al., 2015; Qian et al., 2022; Von Ammon et al., 2025). In the future, combining environmental data with temporal metabarcoding datasets, along with appropriate routine biofouling mitigation, could support accurate early-warning systems for phytoplankton community shifts, thereby improving our understanding of ecosystem dynamics and informing conservation and management strategies (Djurhuus et al., 2018).

These same environmental variables and organismal traits can also influence consistently observed abundance patterns and sporadic detection of marine NIS, such as *Magallana spp*. and *A. senhousia*, compared to *Botrylloides spp*. and *Styela plicata*, respectively. For instance, eDNA approaches have been shown to be particularly sensitive for bivalves, likely due to DNA shedding from both adults and planktonic larvae and their localised presence (Amberg et al., 2019; Prié et al., 2023; Sansom & Sassoubre, 2017; Yang et al., 2025). In contrast, Couton et al. (2022) reported difficulty detecting *Botrylloides violaceus* despite its known presence. Leathery tunicates such as *Styela clava* tend to have lower detection rates, whereas organisms with more fragile morphologies, such as the Mediterranean fanworm *Sabella spallanzanii*, show higher detection rates (Scriver et al., 2024b; Wood et al., 2020). While other bryozoans like *Bugula neritina* can exhibit intermediate or variable detection, likely due to their interconnected zooids enclosed within a calcified exoskeleton (Scriver et al., 2024b). Additional challenges from metabarcoding marker performance, including differential DNA extraction efficiency, primer bias and non-specificity, and the conserved nature of some loci, can limit species-level resolution (Xiong, Li & Zhan, 2016). Therefore, metabarcoding should be used primarily as an initial screening tool, and it is always advisable to perform complementary species-specific assays or visual confirmation to ensure accurate NIS identification (Scriver et al., 2024a; Von Ammon et al., 2018; Von Ammon et al., 2025).

Extended temporal sampling is critical. Daily sampling can capture high variability, such as the intermittent spikes in Malacostraca (amphipods), Polychaeta, and Bivalvia observed in the COI dataset and enhances marine NIS detection, but it may be too dynamic and unstructured to detect consistent ecological trends (Sevellec et al., 2025). Weekly sampling, as observed here, revealed more pronounced shifts that are likely more indicative of meaningful ecological changes. At larger temporal scales, shifts in composition could also be used to signal anthropogenic pressures. For example, DiBattista et al. (2020) observed replacement of keystone taxa, such as arthropods, with opportunistic taxa from phyla like Annelida and Mollusca, under anthropogenic stress. These results suggest that weekly sampling over a longer period (several months or more), combined with protocols to manage biofouling, may offer a holistic view of biodiversity trends and provide insights into ecosystem health while balancing cost and resource demands.

However, while community composition was more consistent within a given week than between weeks, weekly sampling may not be sufficient if the goal is rare species detection. Many studies have shown high variability in eDNA signals for fish and metazoans over 24-hour periods in marine environments, suggesting that temporal sampling should occur at multiple time points to capture species’ behavioural event patterns (Courtaillac et al., 2024; Ely et al., 2021; Jensen et al., 2022; Monuki, Barber & Gold, 2021; Rossouw et al., 2024) and detect rare targets such as marine NIS (Scriver et al., 2024a). Studies recommend sampling at least twice within 24 h, ideally 12 h apart, and collecting three to four samples for rare or low-abundance species (Courtaillac et al., 2024). Recent work on passive eDNA samplers also highlights the role of deployment time in detection sensitivity: even short submergence periods (10 min) can recover substantial biodiversity, while extended deployments (720 min; 12 h) increase the likelihood of detecting marine NIS, emphasising the importance of temporal replication for active sampling (Von Ammon et al., 2025).

Overall, these results underscore the importance of extended temporal sampling and careful biomonitoring programme design to align sampling frequency and strategies with community groups and monitoring objectives.

### How does autonomous environmental DNA sampling compares to complementary marine non-indigenous species detection methods

A key consideration for local Māori communities is the detection and management of marine NIS, ensuring co-governance within existing science frameworks (Von Ammon et al., 2023a; Von Ammon et al., 2023b). Alongside selecting molecular tools that complement existing biosecurity and biomonitoring systems (Varrella et al., 2025; Von Ammon et al., 2018; Westgaard et al., 2024; Zhao et al., 2021), sampling methodologies must be effective at detecting marine NIS, fit for purpose, practical, and resource-efficient (Fernandez et al., 2021; Von Ammon et al., 2018; Zaiko et al., 2018).

An advantage of the chosen study site was the availability of prior eDNA data and its inclusion in the New Zealand Marine High-Risk Site Surveillance (MHRSS) biannual programme, providing complementary datasets. Scriver et al. (2024a) conducted traditional hourly point sampling using vertical plankton tows and filtration at the same location, employing similar metabarcoding and NIS screening protocols. This enabled comparison between automated eDNA sampling over several weeks and single-day point sampling in terms of NIS detection and the influence of sampling regimes. MHRSS reports further provide annual context for how traditional biosecurity surveys compare to molecular marine NIS detection methods (Woods et al., 2024; Woods et al., 2021b).

Across both metabarcoding markers, automated eDNA sampling at Ōpua Marina/Waikare Inlet captured 17 marine NIS, comparable to the MHRSS surveys (2020–2021: *n* = 17; 2023–2024: *n* = 18) and previous traditional point-sampling eDNA campaign (*n* = 18) (Table S4). This suggests that automated eDNA methods using multi-marker approaches can detect similar numbers of marine NIS as conventional biosecurity surveys and eDNA sampling methods.

While total NIS counts were similar, diversity varied across methods (Fig. S7; Table S4), reflecting seasonal and interannual dynamics and inherent differences between molecular and conventional biomonitoring surveys and NIS screening. Scriver et al. (2024a) sampled in austral spring in 2020, whereas the automated eDNA sampler operated in austral summer in 2023 to 2024. Changes in biological dynamics (*e.g.*, abundance and life stage), eDNA processes (production and degradation), and external pressures such as propagule pressure and management action can strongly influence detection (Collins et al., 2022; De Souza et al., 2016; Salter, 2018; Troth et al., 2021; Yang et al., 2025). For example, many biofouling species, such as tunicates and bryozoans, are highly seasonal (Couton et al., 2022), and in this study, increased 18S rRNA detection of ascidians during summer may reflect seasonal abundance, consistent with Von Ammon et al. (2018), while COI detection remained consistent across seasons. Differences between metabarcoding and traditional morphological detection also reflect methodological constraints. Primer bias, amplification inefficiencies and incomplete reference databases can affect metabarcoding screening for marine NIS (Keck, Couton & Altermatt, 2023; Mugnai et al., 2023; Van der Loos & Nijland, 2021; Varrella et al., 2025), while eDNA-specific factors such as shedding rates, preservation, environmental dilution, and limited understanding of eDNA ecology further impact the interpretation of the results (Rishan, Kline & Rahman, 2023). For example, the Pest Alert tool, designed specifically for Aotearoa-New Zealand marine NIS, includes many entries for NIS, unwanted, and notifiable organisms; however, it does not encompass all taxa, likely due to gaps in available reference sequences. This helps explain why *Agnezia sp*., absent from the Pest Alert database, was detected in the MHRSS survey but not in either eDNA metabarcoding campaign. Conversely, traditional surveys may miss cryptic taxa or early life stages (*e.g.*, meroplanktonic larvae) and require greater field effort and taxonomic expertise to reach comparable detection sensitivity (Rishan, Kline & Rahman, 2023; Stat et al., 2017). This could explain why the bivalve *Magallana gigas* was detected by eDNA metabarcoding but not during the MHRSS survey, likely due to the presence of meroplanktonic larvae rather than adult stages (Varrella et al., 2025).

Overall, the results indicate that automated sampling with metabarcoding can detect marine NIS and functions as a complementary tool for biosecurity programmes. A long-term advantage is the capacity to collect high-temporal-resolution eDNA data, capturing seasonal and annual dynamics (Sevellec et al., 2025), which improves early detection and monitoring of NIS populations.

### Study Limitations

This case study provides valuable insights and allowed local Māori community groups to test the usability of automated eDNA sampling for marine biosecurity and biomonitoring. However, the dataset was limited, and there were malfunctions and device management issues. Despite these constraints, the study was novel and offers recommendations to guide larger, long-term co-designed biomonitoring projects.

Operational challenges, such as battery failures and occasional mishandling of filters, reduced the number of usable samples, limiting the ability to evaluate biodiversity patterns or optimise sampling regimes. For example, the loss of week 2 and the limited data from week 3 made it difficult to assess stable weekly patterns or evaluate the influence of autosampler temperature and other environmental conditions on biodiversity and marine NIS detection. Determining the optimal number of samples per day or week, as in Jones et al. (2024), is essential for improving detection while minimizing cost, effort, and human resources, which is fundamental for integrating these systems into routine biomonitoring.

As noted above, in this study, extensive biofouling on intake pipes could influence detected biodiversity patterns, introduce background noise, and reduce the detection of rare taxa (Von Ammon et al., 2025). For example, in the COI dataset, we observed a small but significant increase in the relative read abundance of Ascidiacea (sessile biofouling organisms) by week 4, accounting for less than 1% of total reads across all weeks (Fig. 3). The influence of biofouling in intake pipes was further evident in post-deployment controls, where distilled water was run through the system. In these controls, genera such as *Barentsia*, *Parietochloris*, *Cladophora, Neosiphonia*, *Ectopleura,* and *Austrominius* were detected (Figs. S8 & S9), suggesting that early-settler biofouling organisms can accumulate in intake pipes and could generate low-level background signals. These taxa were removed during downstream analyses, highlighting the importance of appropriate controls to identify and remove noise. While this data-cleaning step minimises bias when monitoring core communities, it may also remove true detections of rare taxa or potentially marine NIS, as biofouling-based sampling itself can serve as a tool to detect pests (Azevedo et al., 2020; Perry et al., 2026; Von Ammon et al., 2025). Consequently, careful consideration of biofouling is critical, particularly when the goal is to track temporal trends. Minimising biofouling during deployment reduces the need for extensive data removal and improves the reliability of temporal assignments, especially in marine environments where submerged materials are unavoidable. For automated samplers, this must be balanced with operational ease. Daily cleaning, for example, is often infeasible, so antifouling measures should be safe, practical, and non-interfering with eDNA detection (Nam et al., 2026). Innovative strategies to minimise fouling could include bubble streams (Hopkins et al., 2021), non-toxic antifouling coatings (Zang et al., 2024), and bio-inspired antifouling materials (Nir & Reches, 2016; Tian, Jin & Tian, 2022).

Carryover contamination and residual eDNA in pipework between sampling campaigns and sites remain key issues for automated samplers in both freshwater and marine systems (George et al., 2024; Preston et al., 2023; Truelove et al., 2022). In this study, a post-deployment control collected after the autosampler’s internal wash (flushing distilled water) still contained eDNA sequences, indicating residual contamination (Figs. S8 & S9). This aligns with George et al. (2024), who found eDNA in most field blanks despite flushing with 5 L of deionised water, suggesting DNA persistence in intake screens, tubing, or internal components. Preston et al. (2023) demonstrated that flushing with a weak bleach solution between samples, along with increased site water flushing before collection, reduces residual DNA carryover. Other mitigation strategies include incorporating contamination controls in data analysis, using low-binding plastics, developing filter-forward autosamplers, and avoiding shared intake lines (George et al., 2024; Hendricks et al., 2023; Yamahara et al., 2019).

Compared to other conventional eDNA sampling methods, the Smith-Root eDNA Autosampler has specific limitations that could influence the results. The autosampler collected localised, small-volume samples from a fixed inlet, reducing spatial and vertical coverage compared to methods such as plankton tows, which sample larger water volumes across depths (Scriver et al., 2024a; Scriver et al., 2024b). Volume filtered is critical; the autosampler occasionally clogged, filtering sometimes less than 1 L per sample. Studies have demonstrated that higher water volumes increase eDNA detection sensitivity, particularly for rare or low-abundance NIS (Cantera et al., 2019; Couton et al., 2022; Schabacker et al., 2020; Shu, Ludwig & Peng, 2020). However, the labour-intensive nature of using some traditional eDNA sampling devices, such as plankton nets in the field, combined with the time-consuming filtering step, suggests a trade-off between sample frequency, duration, and effort *versus* increasing temporal sampling and volume filtered.

We see the ultimate value of temporally extended biodiversity surveys in dynamic coastal environments, aided by automated sampling, as the provision of well-resolved ‘cumulative’ biological information over time. The data gathered in this way represent varying environmental and hydrodynamic conditions alongside temporal variability in communities and eDNA dynamics, which may not be effectively captured by a one-off sampling campaign. For routine surveillance, it would be difficult and expensive to achieve similar resolution with temporal or spatial replication and manned sampling approaches.

### Community engagement and recommendations for future use

As the first study to deploy the Smith-Root eDNA Autosampler in Aotearoa-New Zealand, it was important to engage with potential end-users, in this case Ngāti Manu community, and provide feedback and recommendations for future use. This included collecting feedback from the local community regarding implications for both the process and outcomes (Supplementary Data, Appendix 3). Engagement with local Māori communities began in early 2023 with initial discussions focused on potential installation sites and subsequent collaborative conversations during the device’s deployment.

Māori community feedback highlighted that the eDNA dataset is viewed as a valuable mechanism for tracking environmental change within the Kāretu watershed, particularly in the context of accelerating climate impacts (*e.g.*, intensified storm events, erosion, and hydrological instability) and ongoing pressures such as land-use change, pollution, sea-level rise, and biodiversity loss. These environmental changes have direct implications for marae, kāinga, taonga species, and culturally significant sites. The dataset was considered useful for strengthening local knowledge systems and supporting long-term socio-environmental planning. Ngāti Manu emphasised that understanding ecological shifts is essential for collective adaptation and long-term decision-making, and that the data generated through this study contributed meaningfully toward these goals.

Beyond generating biodiversity data, Ngāti Manu reported that the project contributed to local capability development and strengthened engagement at the mātauranga–science interface. Youth participation was identified as especially important, supporting intergenerational knowledge transmission, fostering local leadership development, and strengthening relationships among neighbouring hapū (Supplementary Data, Appendix 3).

While overall feedback was positive and the tool was reported as meeting expectations, Ngāti Manu recommended deeper integration of mātauranga Māori into future deployment design, sampling strategies, and data interpretation (Supplementary Data, Appendix 3). Incorporating engagement and co-design into future deployments can enhance both scientific outcomes and the potential to resonate with and better support the needs and aspirations of local communities, while fostering intergenerational knowledge sharing and local stewardship of aquatic ecosystems. Future deployments should therefore formalise co-design processes, embed mātauranga alongside molecular data streams, and prioritise community capability building to ensure that scientific outputs align with Māori community aspirations and local environmental stewardship objectives.

From a technical perspective, while the eDNA Autosampler performed well overall, several suggestions emerged that could be implemented to enhance the device’s applicability in marine environments and optimise its use in the field.

Based on findings from this study and the literature, the following recommendations are proposed for using the eDNA Autosampler, particularly in marine settings:

1. User Training and Operation: Provision of hands-on demonstrations for field operators, along with clear protocols for filter installation and troubleshooting, is recommended to minimise sample loss due to improper filter loading.
2. Battery and Power Management: Source several batteries compatible with the device to ensure seamless changeover and back-up opportunities. Ensure batteries are fully charged before deployment. Batteries should be monitored and replaced as needed during longer deployments to prevent data loss.
3. Regular Maintenance to Prevent Fouling: More periodic cleaning, or the use of cost-effective and appropriate anti-fouling measures (*e.g.*, low binding or anti-fouling materials) is recommended for extended marine deployments. Another consideration is the use of alternative materials for the strainers, such as copper, which may help reduce rust.
4. Thermal Management: Additional insulation or shading may be necessary in exposed locations or during warmer months to maintain stable internal temperature.
5. Contamination Risk and Mitigation: To minimise cross-site contamination, follow the manufacturer’s sterilization procedure (Supplementary Data, Appendix 2) for decontaminating the unit with bleach solutions between deployments. Before relocating the device to a new sampling site, replace intake hoses and strainers and increase the volume of site water used for flushing both prior to sample collection and between samples.
6. Field Controls: To detect and minimise biofouling-related noise in eDNA datasets, particularly when assessing temporal trends, collect field controls after flushing the system with freshwater. Ideally, these should be taken at the end of deployment and at regular intervals, such as between filter changes. They allow identification of biofouling taxa accumulating in intake pipes, so their signals can be removed during downstream analyses. Field controls should be used alongside regular maintenance procedures to reduce noise and minimise taxonomic bias in temporal eDNA datasets.

The further optimization and development of mobile, high-resolution eDNA platforms offers the potential to enhance sampling across both temporal and spatial scales. For example, next-generation autonomous samplers such as the third-generation Environmental Sample Processor (ESP) are now being adapted for deployment on autonomous underwater vehicles (AUVs) (Yamahara et al., 2019). These devices can collect and process up to 60 samples while operating freely in the ocean for extended periods, offering high spatial and temporal resolution that would be difficult to achieve through manual methods. In addition, advances in artificial intelligence and machine learning have the potential to further enhance these systems by enabling targeted sampling and real-time data processing for faster ecological monitoring and response (Yamahara et al., 2025).

Despite their promise, these systems still face several challenges and barriers, including the need for validation, robust deployment controls, and standardised device specifications and reporting (Yamahara et al., 2025). In addition, a key limitation remains the high cost of automated systems, with some devices ranging from $55,000 to over $100,000 USD (Hendricks et al., 2023), which presents a significant barrier to wide-scale adoption, particularly for under-resourced jurisdictions, small scientific projects, and community-led initiatives.

## Conclusions

This study demonstrated the successful use of the Smith-Root eDNA Autosampler in a coastal marine environment to detect local biodiversity patterns and screen for marine NIS. The sampler performed reliably, with two metabarcoding markers (COI and 18S rRNA) providing complementary insights on local biodiversity.

Weekly sampling revealed significant temporal shifts in community composition, suggesting that increasing temporal resolution (*e.g.*, weekly sampling) can improve the detection of ecological turnover. While more frequent sampling (*e.g.*, daily or multiple times a day) may further enhance the detection of event-driven or rare targets (*e.g.*, marine NIS). However, it is critical to implement adequate biofouling removal protocols to minimise potential “noise” from fouling organisms, which can bias the observed community composition.

Collaboration with the local Māori communities enabled the trial of simplified sampling protocols with the autosampler, demonstrating that it can be operated with minimal technical training. This integration of community co-design with key technical optimizations (cleaning procedures, battery maintenance, thermal protection, and contamination mitigation) could maximise the usability and reliability of the instrument in the field.

One key advantage of automated samplers is their flexibility, they enable extended temporal sampling without substantially increasing labour demands, with the addition of cost savings for eDNA surveillance at remote or hard-to-access sites (Sepulveda et al., 2020). This flexibility can enhance the resolution of ecological patterns and significantly improve the detection of species-specific targets such as rare or invasive taxa. Ultimately, implementing careful sampling strategies that account for marine environmental conditions helps reduce false negatives and noise, supports early detection of marine NIS and other hard-to-detect species, and enables the capture of true temporal environmental signals.

##  Supplemental Information

10.7717/peerj.21287/supp-1 Supplemental Information 1 Supplementary Figures and Tables

10.7717/peerj.21287/supp-2 Supplemental Information 2 Smith-Root eDNA Autosampler Service Protocol for the Opua Deployment

10.7717/peerj.21287/supp-3 Supplemental Information 3 Smith-Root eDNA Autosampler Manual

10.7717/peerj.21287/supp-4 Supplemental Information 4 Feedback report from the local Māori community of Ngāti Manu

10.7717/peerj.21287/supp-5 Supplemental Information 5 18S rRNA Data Analysis Script Post-Bioinformatics

10.7717/peerj.21287/supp-6 Supplemental Information 6 COI Data Analysis Script Post-Bioinformatics

10.7717/peerj.21287/supp-7 Supplemental Information 7 Analysis Code for Comparing eDNA Sampling Methods: Smith-Root Autosampler vs Traditional Point Sampling

## Additional Information and Declarations

### Field Study Permissions

The following information was supplied relating to field study approvals (*i.e.*, approving body and any reference numbers): Field experiments were approved by the Cawthron Institute. Cawthron holds a Special Permit with the Ministry for Primary Industries (SP822-2) that allows the taking of fish, aquatic life and seaweed for the purposes of education and investigative research.

### Data Availability

The following information was supplied regarding data availability: The data and code are available in the Supplemental Files. The raw sequence data are available at NCBI BioProject: PRJNA1332291.
