# Supplementary material for: Automated eDNA sampling for marine monitoring and biosecurity: optimising temporal resolution, remote deployments, and community engagement
Source: PeerJ. 2026 May 28;14:e21287. doi: 10.7717/peerj.21287 (PMC13222548; doi:10.7717/peerj.21287)
Supplement: Supplemental Information 1 [file peerj-14-21287-s001.docx]

**Automated eDNA sampling for marine monitoring and biosecurity: optimising temporal resolution, remote deployments, and community engagement**

Supplementary Data


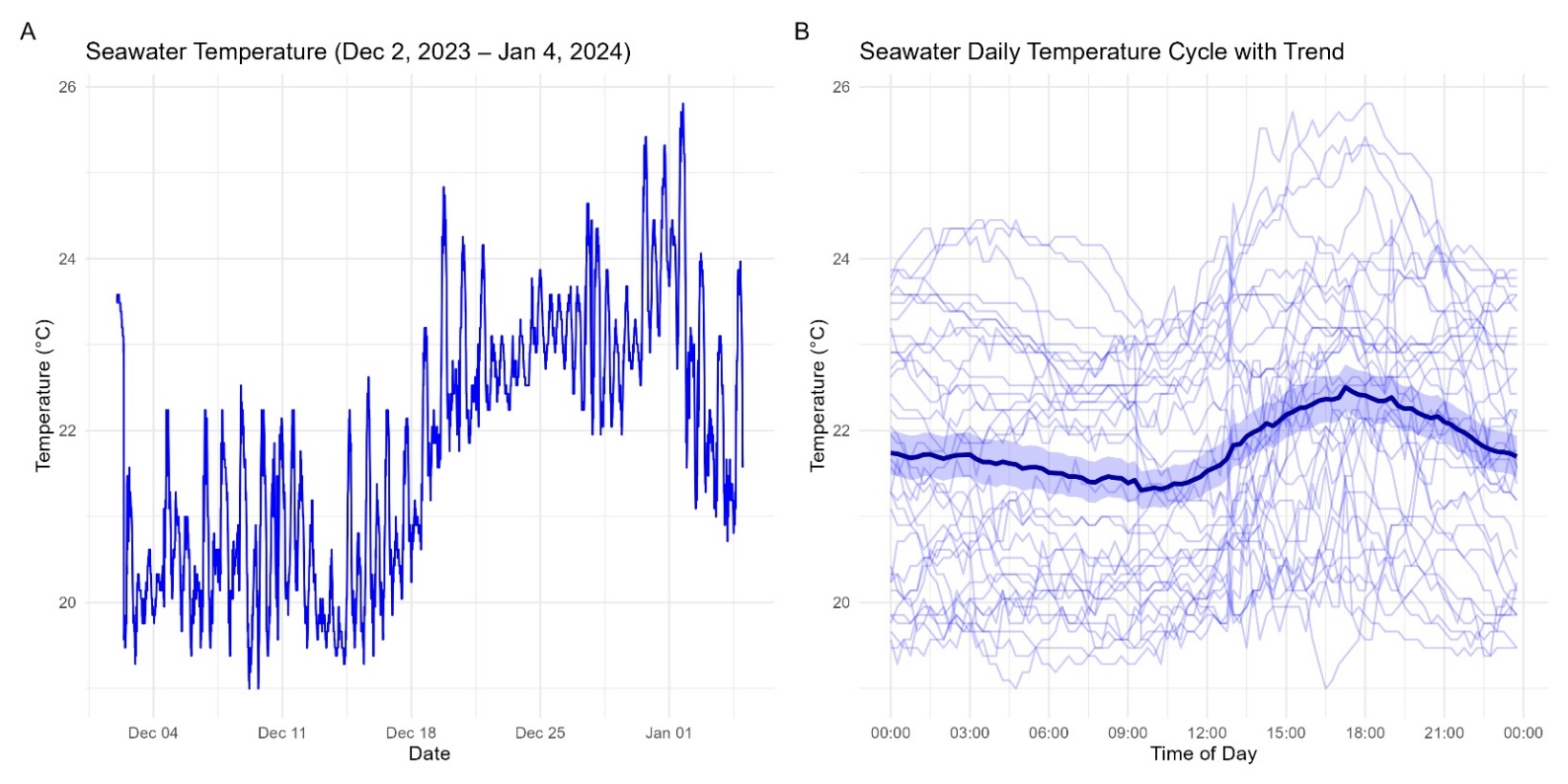


**
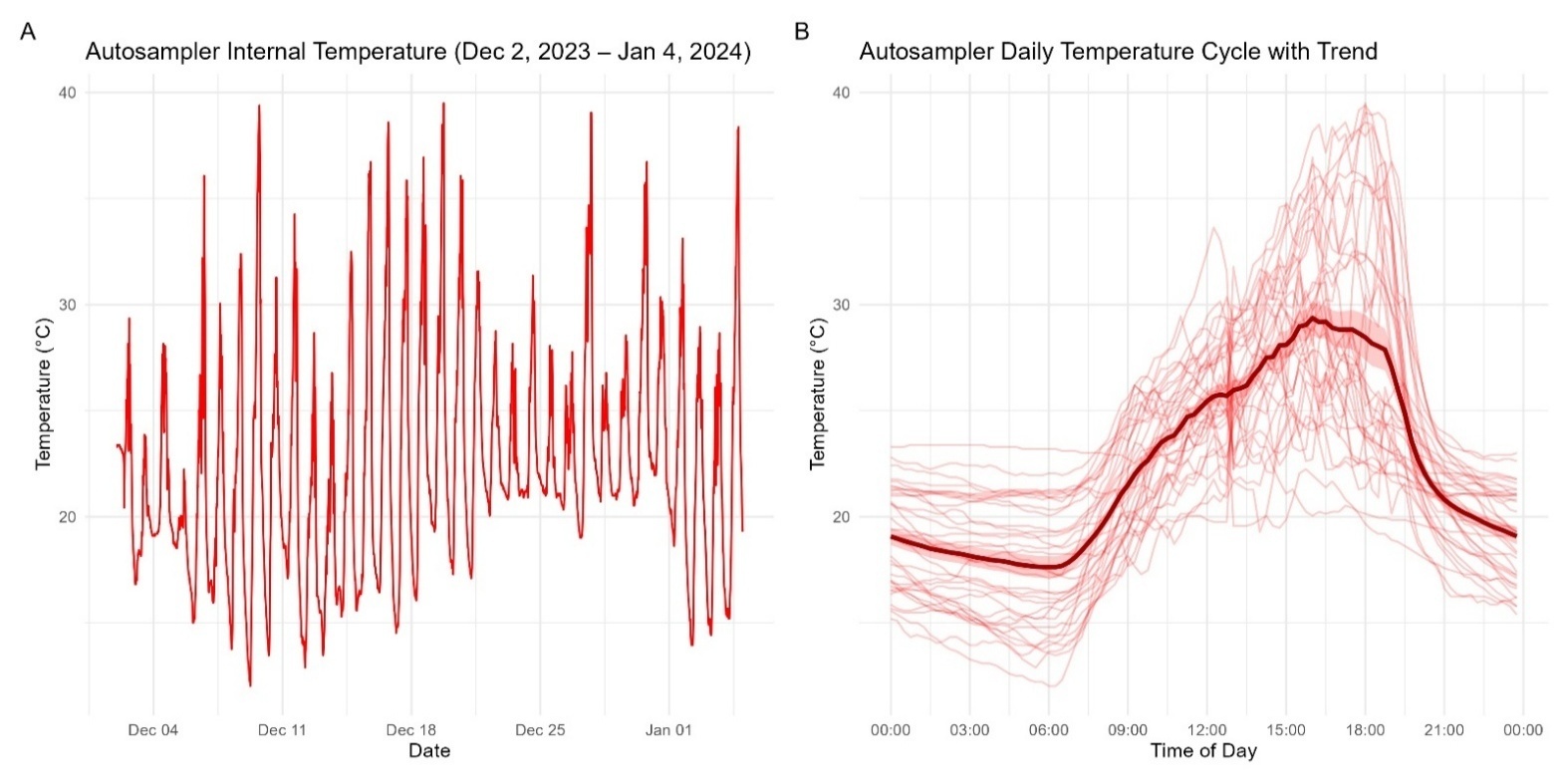
Figure S1.** Temperature (ºC) logs from seawater measured with a HOBO logger at the deployment site in Bay of Islands Marina, Ōpua, Aotearoa New Zealand, from 2 December 2023 to 4 January 2024. (**A**) Daily changes; (**B**) Hourly temperature changes with an average trend line by calculated using ggplot2.

**Figure S2.** Internal temperature (ºC) logs from the Smith-Root eDNA autosampler (Smith-Root, USA), measured with a HOBO logger placed inside the instrument deployed at Bay of Islands Marina, Ōpua, Aotearoa New Zealand, from 2 December 2023 to 4 January 2024. (**A**) Daily temperature changes; (**B**) Hourly temperature changes with an average trend line calculated using ggplot2.


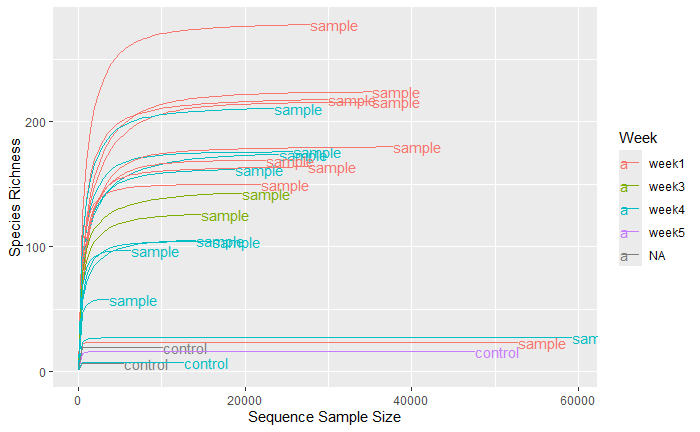


1. 18S rRNA

**B)** COI

**
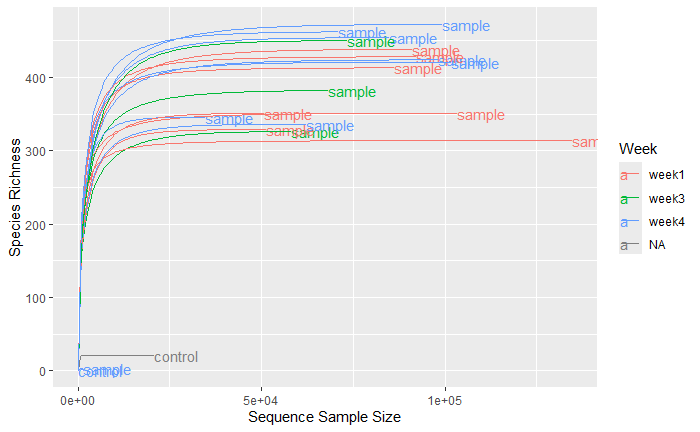
**

**Figure S3.** Rarefaction curves for **(A)** small ribosomal subunit RNA (18S rRNA) and **(B)** mitochondrial *Cytochrome C Oxidase subunit* I (COI) genes from metabarcoding data by sampling week.

**Figure S4.** Alpha diversity measured as Observed amplicon sequence variants (ASVs) for the **(A)** small ribosomal subunit RNA (18S rRNA) and **(B)** mitochondrial *Cytochrome C Oxidase subunit* I (COI) genes datasets. Data is grouped by week of sampling. Significance is indicated by letters , where sampling weeks sharing a letter are not significantly different, and weeks with different letters differ significantly. Note ‘week2’ was not sampled due to deplete battery.

**A)** 18S rRNA

**B)** COI


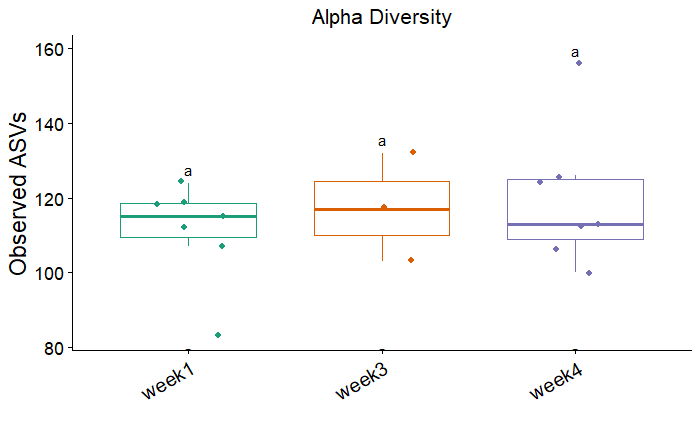

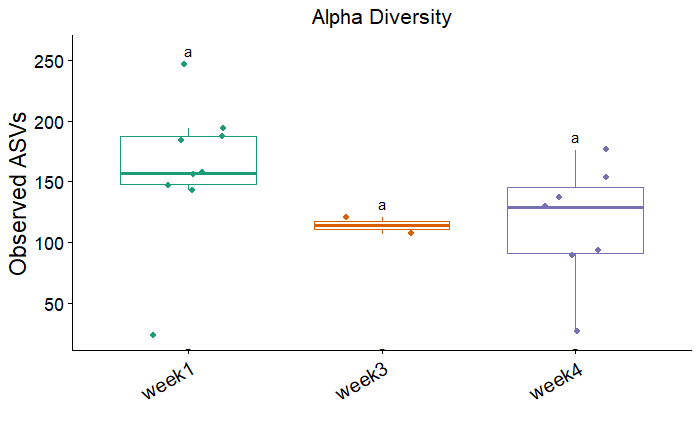

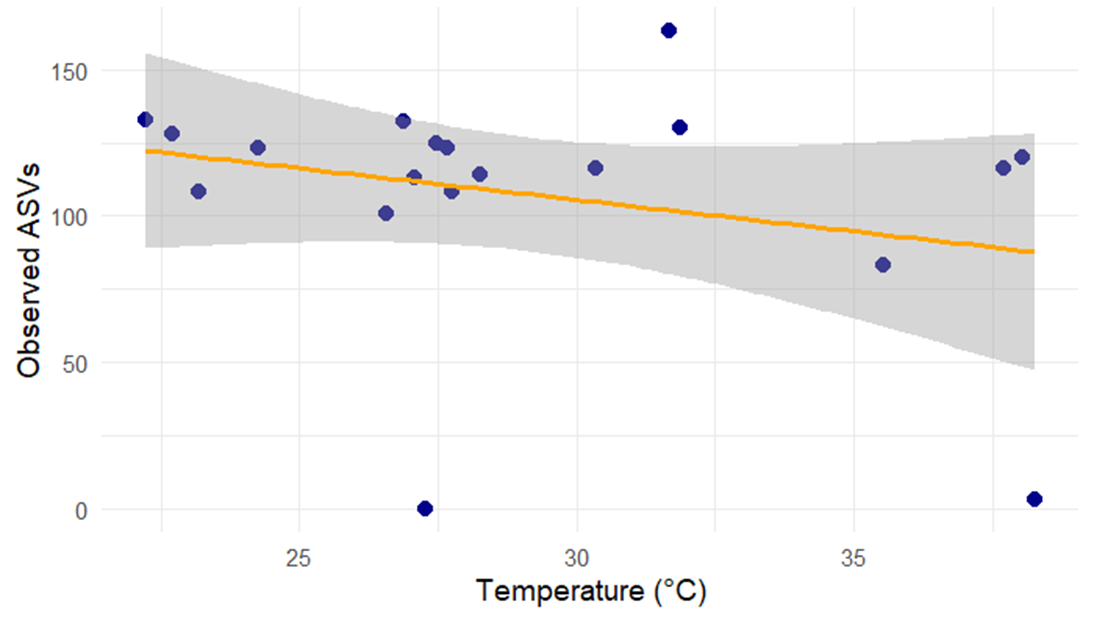

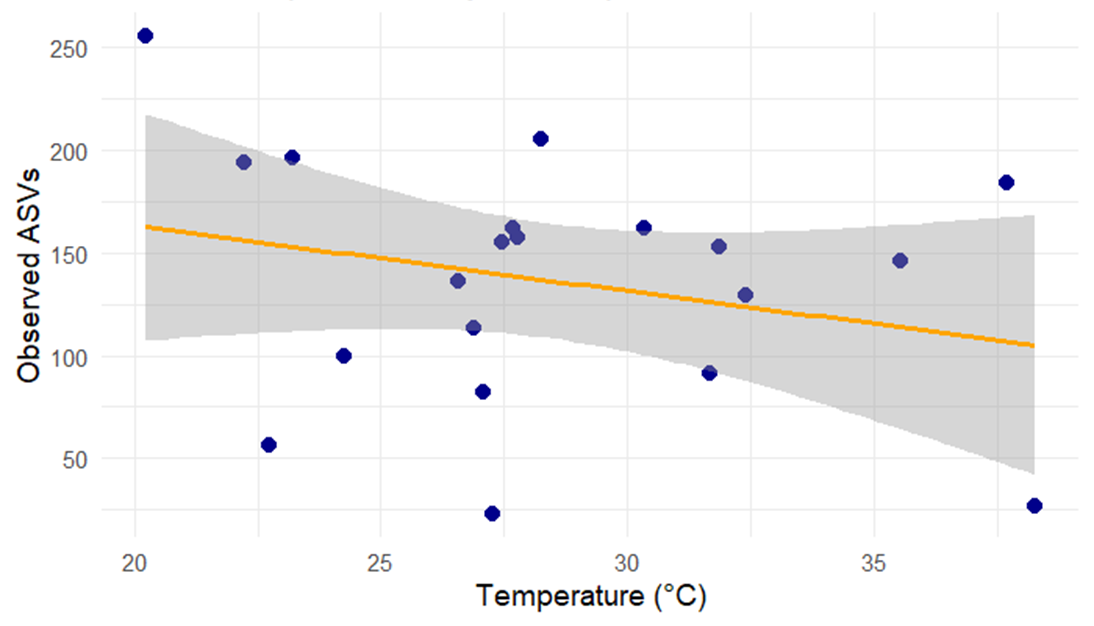


**A)** 18S rRNA

*r=-0.262, p=0.265*

**B)** COI

*r=-0.271, p=0.261*

**Figure S5.** Correlation between temperature recorded by the Smith-Root eDNA autosampler (Smith-Root, USA) and the number of observed amplicon sequence variants (ASVs), used here as a measure of alpha diversity, for (**A**) the small subunit ribosomal RNA gene (18S rRNA) and (**B**) the mitochondrial *Cytochrome C Oxidase subunit* I gene (COI). For 18S rRNA, quadratic regression confirmed that temperature did not explain a meaningful amount of variation (Adjusted R² = -0.00992, *p* = 0.4225). For COI, the quadratic model explained slightly more variation but still not significant (Adjusted R²= -0.0250, *p* = 0.475).


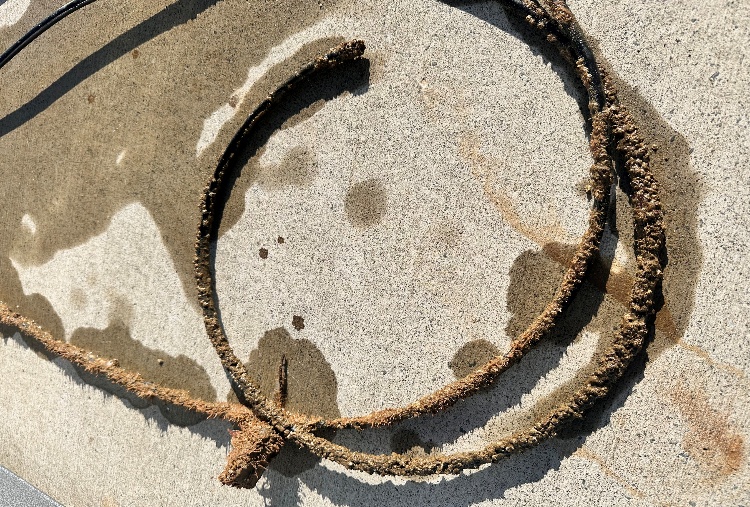

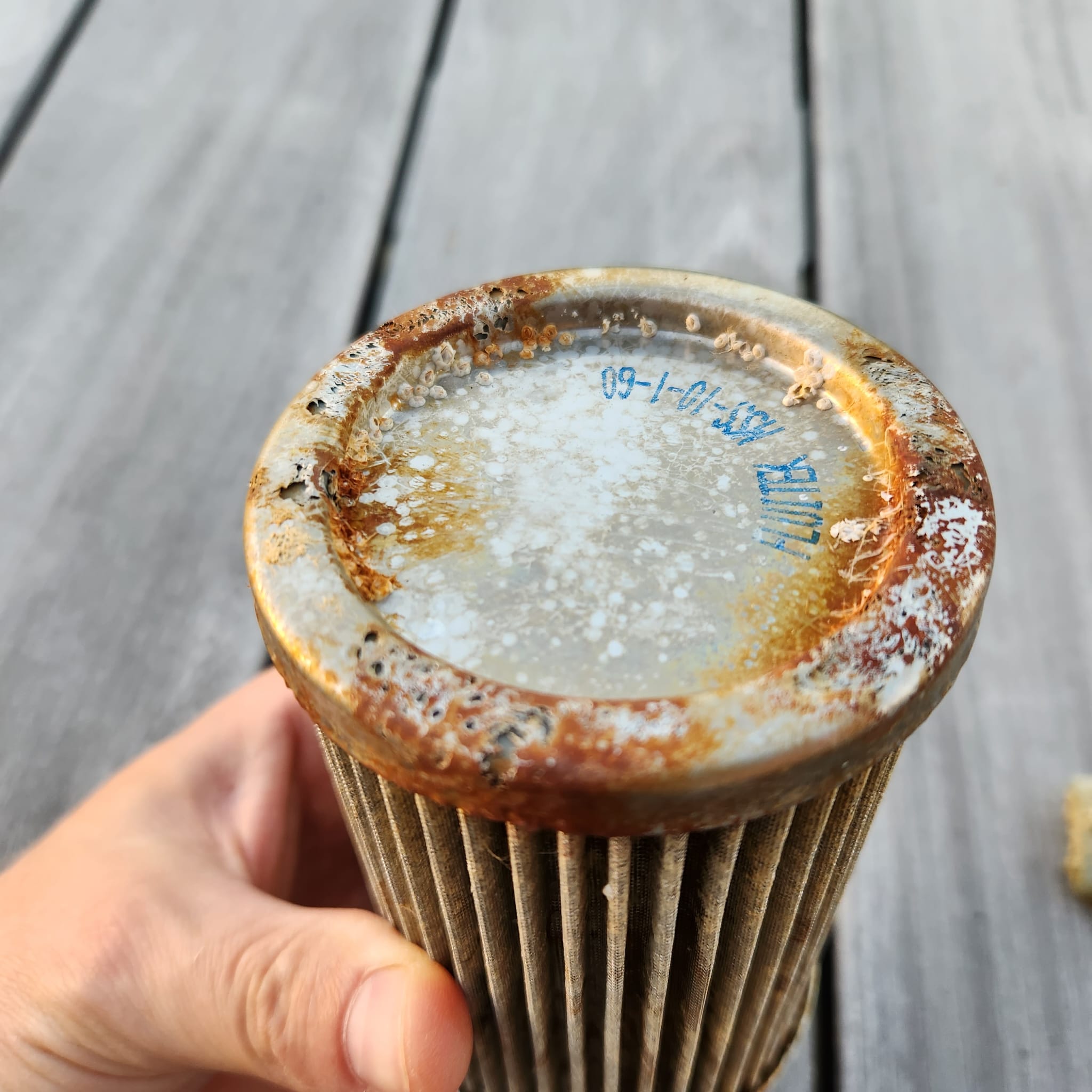

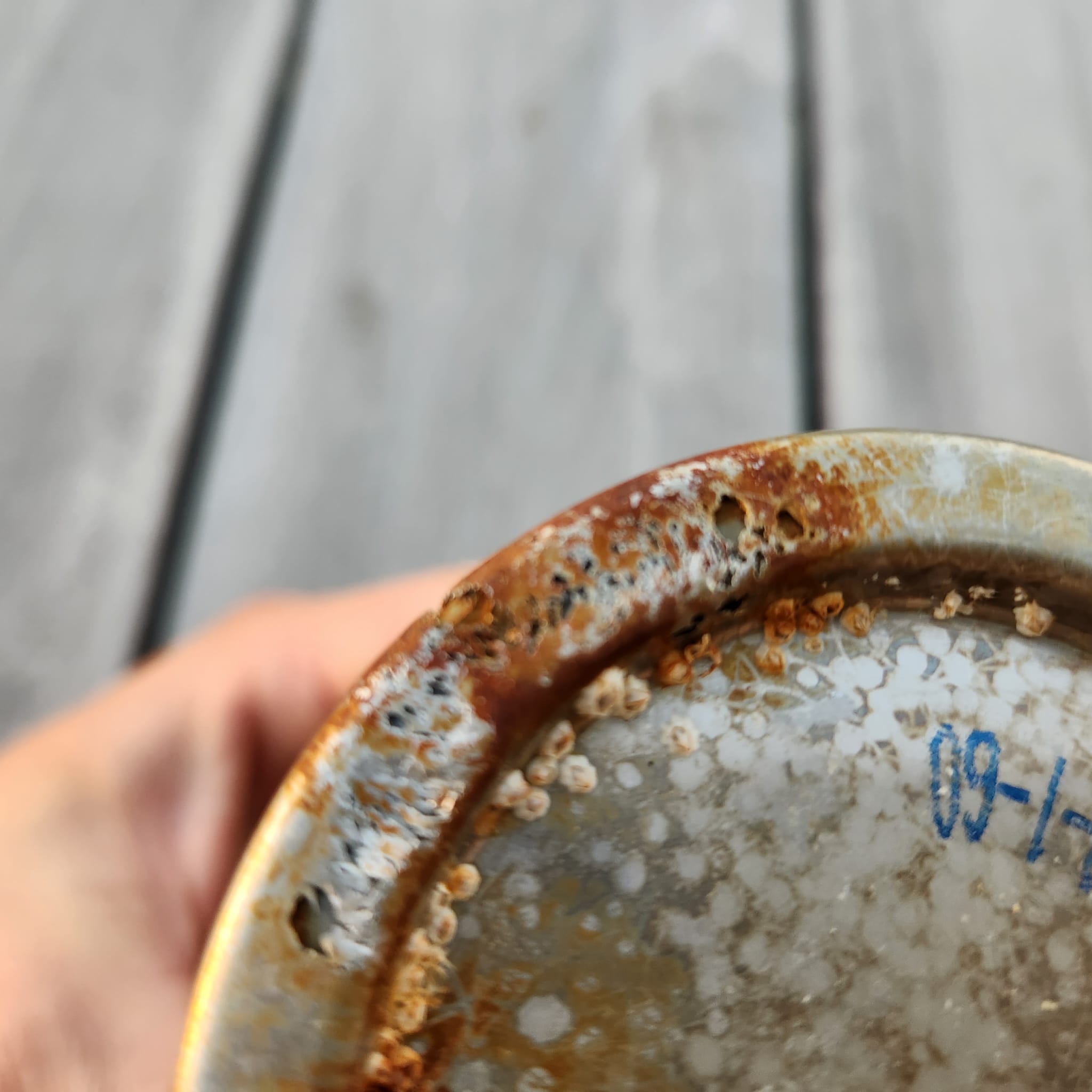

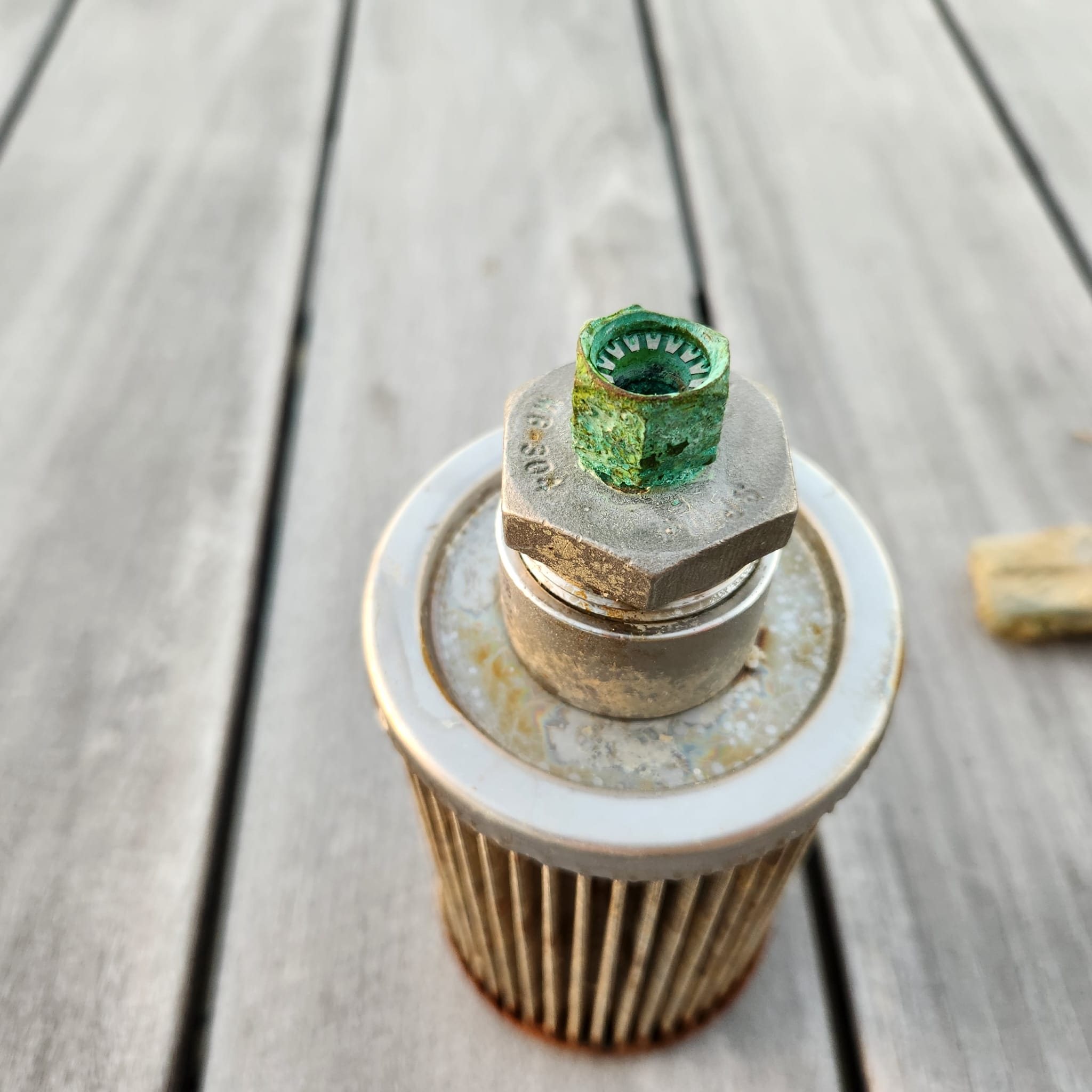


**Figure S6.** Observations of (**A**) the 2-meter intake hose and (**B**) the mesh strainer following deployment of the Smith-Root eDNA Autosampler in the Bay of Islands Marina, Ōpua, Aotearoa-New Zealand, from 2 December 2023 to 4 January 2024.

**
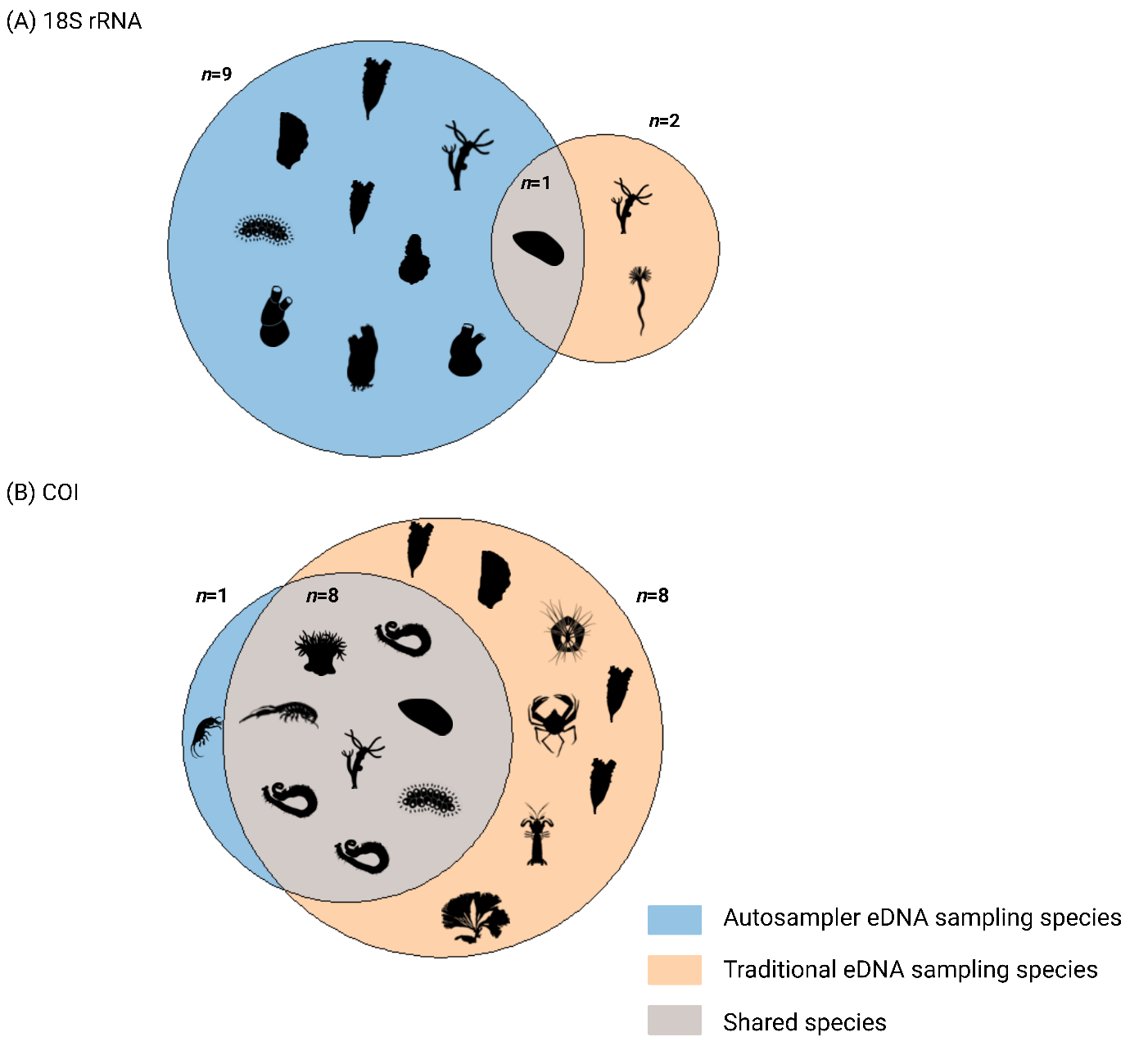
Figure S7.** Euler plot comparing filtration methods for detecting marine non-indigenous species (NIS) (A) small ribosomal subunit RNA (18S rRNA) and (B) mitochondrial Cytochrome C Oxidase subunit I (COI) gene datasets across two studies in Bay of Islands Marina, Ōpua, Aotearoa-New Zealand. Data include marine NIS detected in the present study using the Smith-Root Environmental DNA (eDNA) Sampler (“Autosampler eDNA sampling species,” blue) and in Scriver et al. (2024a) using traditional point sampling with a vertical plankton tow net (“Traditional eDNA sampling species,” orange). Species detected by both methods are shown in grey. Species silhouettes from PhyloPic.org; final figure created with BioRender.com.

**
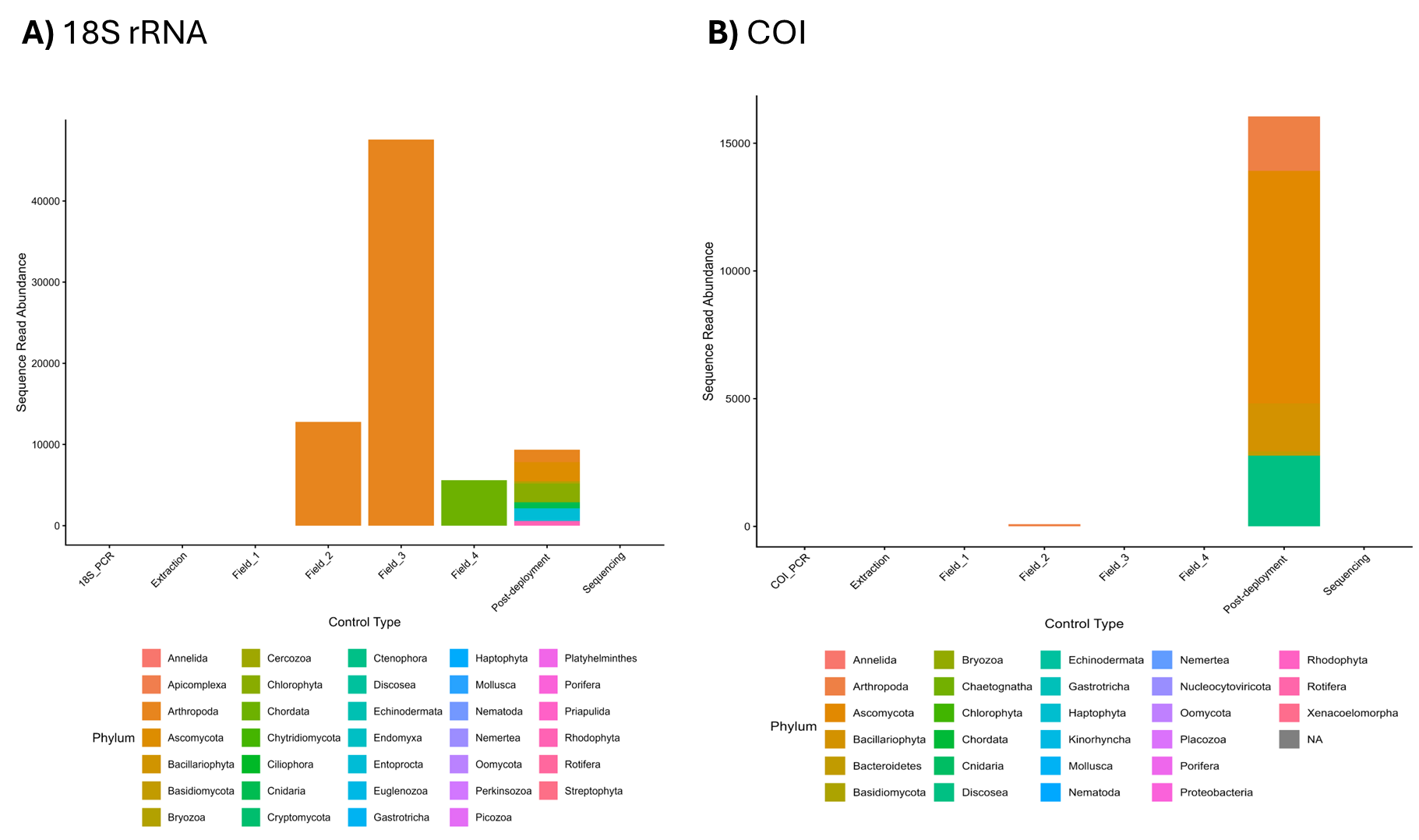
**

**Figure S8**. Bar plot depicting the sequencing reads present in the control samples, including both laboratory controls (extraction, PCR, and sequencing) and field controls (Field 1–4 and post-deployment control), for (**A**) the small subunit ribosomal RNA gene (18S rRNA) and (**B**) the *Cytochrome C Oxidase subunit* I (COI) metabarcoding datasets. Amplicon sequence variants (ASVs) detected in controls were subtracted from the samples prior to downstream analyses.


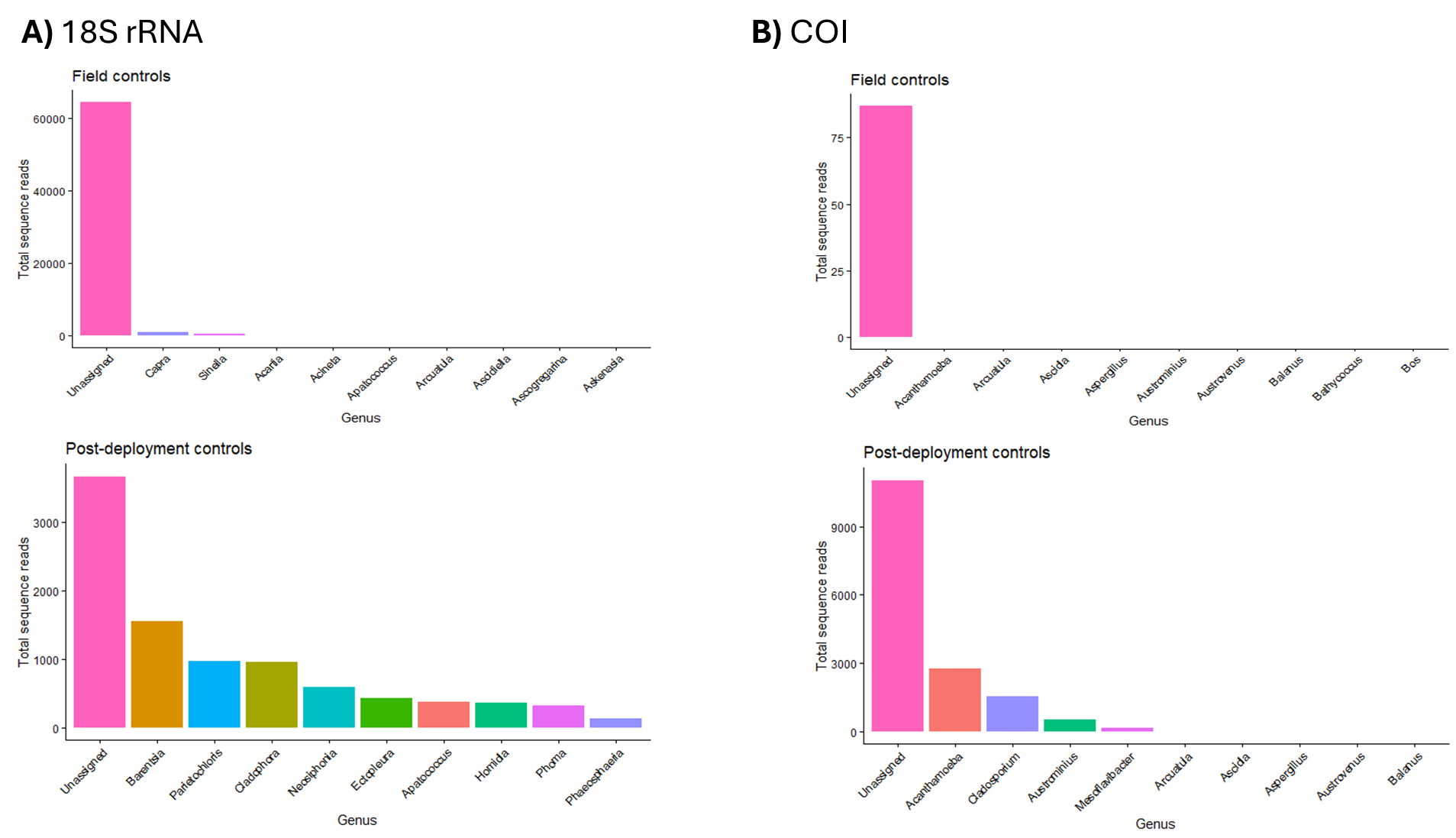
**Figure S9**. Bar plot depicting the sequencing reads from the top ten genus present in the field control samples, grouped by field controls (open and closed filters exposed to the field environment; Field 1-4) and post-deployment control (running distilled water run through the sampling system after deployment), for (**A**) the small subunit ribosomal RNA gene (18S rRNA) and (**B**) the *Cytochrome C Oxidase subunit* I (COI) metabarcoding datasets. Amplicon sequence variants (ASVs) detected in controls were subtracted from the samples prior to downstream analyses.

**Table S1.** Permutational multivariate analysis of variance (PERMANOVA) and heterogeneity (PERMDISP) based on Bray–Curtis dissimilarity distances after a Hellinger transformation data used to assess variation in community composition by sampling (Week).

| 18S rRNA | | | | | | |
| --- | --- | --- | --- | --- | --- | --- |
|  | **Df** | **SumofSqs** | **R2** | **F** | **Pr(>F)** | **Permdisp (p)** |
| Week | 2 | 1.143681 | 0.2223262 | 2.144147 | 0.007** | 0.165 |
| Residual | 15 | 4.000476 | 0.7776738 |  |  |  |
| Total | 17 | 5.144157 | 1 |  |  |  |
| COI | | | | | | |
|  | **Df** | **SumofSqs** | **R2** | **F** | **Pr(>F)** | **Permdisp (p)** |
| Week | 2 | 0.8072406 | 0.2094029 | 1.854068 | 0.003** | 0.528 |
| Residual | 14 | 3.0477228 | 0.7905971 |  |  |  |
| Total | 16 | 3.8549634 | 1 |  |  |  |

**Table Note:** Permutations =999

**Table S2.** Model output from a Poisson generalized linear model assessing the effect of autosampler temperature and sampling week on the number of marine non-indigenous species (NIS) detections based on the small ribosomal subunit RNA (18S rRNA).

| **Variable** | **Estimate** | **Std. Error** | **z value** | **p-value** | **Significance** |
| --- | --- | --- | --- | --- | --- |
| **(Intercept)** | 1.09697 | 0.91131 | 1.204 | 0.2287 |  |
| **autosampler_temp** | -0.03732 | 0.03258 | -1.145 | 0.25203 |  |
| **Week 3** | 0.69697 | 0.60029 | 1.161 | 0.24562 |  |
| **Week 4** | 1.19971 | 0.37464 | 3.202 | 0.00136 | ** |
| glm(formula = nis_detected_count ~ autosampler_temp + Week, family = poisson, data = meta_df) | | | | | |
| Null deviance: 34.718 on 19 degrees of freedom  Residual deviance: 22.853 on 16 degrees of freedom  AIC: 73.87  Number of Fisher Scoring iterations: 5 | | | | | |

**Table Notes:** Significant values are denoted by asterisks: *p* < 0.001 (***), *p* < 0.01 (**), and *p* < 0.05 (*). Marginally significant values (p > 0.05 but < 0.1) are denoted by a dot (.).

**Table S3.** Model output from a Poisson generalized linear model assessing the effect of autosampler temperature and sampling week on the number of marine non-indigenous species (NIS) detections based on the mitochondrial *Cytochrome C Oxidase subunit* I gene (COI).

| **Variable** | **Estimate** | **Std. Error** | **z-value** | **p-value** |
| --- | --- | --- | --- | --- |
| **(Intercept)** | 1.76786 | 1.27536 | 1.386 | 0.166 |
| **Temp** | -0.05152 | 0.04579 | -1.125 | 0.261 |
| **Week 3** | -0.20803 | 0.67001 | -0.31 | 0.756 |
| **Week 4** | -0.03666 | 0.45299 | -0.081 | 0.935 |
| glm(formula = nis_detected_count ~ autosampler_temp + Week, family = poisson,  data = meta_df) | | | | |
| Null deviance: 19.223 on 17 degrees of freedom  Residual deviance: 17.567 on 14 degrees of freedom  AIC: 57.129  Number of Fisher Scoring iterations: 5 | | | | |

**Table S4.** Comparison of marine non-indigenous species (NIS) detected in 2020–2021 and 2023–2024 at Ōpua Marina/Waikare Inlet, Aotearoa-New Zealand. Detections are based on the biannual National Marine High-Risk Site Surveillance (MHRSS) and metabarcoding of the small subunit ribosomal RNA (18S rRNA) and *Cytochrome C Oxidase subunit* I (COI) genes, with results from this study (Dec 2023–Jan 2024) and Scriver et al. (2024a) (Oct 2020).

|  |  | **Metabarcoding Oct 2020** | |  | **Metabarcoding Dec 2023–Jan 2024** | |
| --- | --- | --- | --- | --- | --- | --- |
| **Species** | **NMHRSS Report 2020–2021** | 18S rRNA | COI | **NMHRSS Report 2023–2024** | 18S rRNA | COI |
| ***Acentrogobius pflaumii*** | ✓ |  |  | ✓ |  |  |
| *Agnezia sp.* |  |  |  | ✓ |  |  |
| *Amathia verticillata* | ✓ |  |  |  |  |  |
| *Amphibalanus amphitrite* |  |  | ✓ |  |  |  |
| *Arcuatula senhousia  (Musculista senhousia)†* | ✓ | ✓ | ✓ |  | ✓ | ✓ |
| *Ascidiella spp* |  |  |  |  | ✓ |  |
| *Botrylloides spp* |  |  | ✓ |  | ✓ | ✓ |
| *Botrylloides diegensis* |  |  |  | ✓ |  |  |
| *Botrylloides giganteum* | ✓ |  |  |  |  |  |
| *Bugulina spp* |  |  | ✓ |  |  |  |
| ***Caprella scauroides*** | ✓ |  |  | ✓ |  |  |
| *Celleporaria nodulosa* |  |  |  | ✓* |  |  |
| *Celleporaria umbonatoidea* | ✓ |  |  |  |  |  |
| *Ciona spp.* | ✓ |  |  |  | ✓ |  |
| *Corella eumyota* |  |  |  |  | ✓ |  |
| *Diadumene lineata* |  |  | ✓ |  |  | ✓ |
| *Diplosoma listerianum* |  |  |  | ✓ |  |  |
| ***Ectopleura spp.*** | ✓ |  |  | ✓ | ✓ |  |
| *Ectopleura crocea* |  |  | ✓ |  |  | ✓ |
| *Eudistoma elongatum* | ✓ |  |  | ✓ |  |  |
| *Hydroides ezoensis* |  | ✓ |  |  |  |  |
| *Jassa slatteryi* |  |  |  |  |  | ✓ |
| *Limaria orientalis* |  |  |  | ✓ |  |  |
| *Magallana spp.* |  |  |  |  | ✓ |  |
| *Magallana gigas (Crassostrea gigas)* |  |  | ✓ |  |  |  |
| *Microcosmus spp.* |  |  |  |  | ✓ |  |
| *Microcosmus squamiger* |  |  | ✓ |  |  |  |
| *Monocorophium acherusicum* |  |  | ✓ |  |  | ✓ |
| *Obelia geniculata* |  | ✓ |  |  |  |  |
| *Omobranchus anolius* |  |  |  | ✓ |  |  |
| *Oratosquilla oratoria* |  |  | ✓ | ✓ |  |  |
| *Polyandrocarpa zorritensis* | ✓ * |  | ✓ |  |  |  |
| ***Polycera hedgpethi*** | ✓ |  |  | ✓ |  |  |
| *Polydora cornuta* |  |  | ✓ |  |  | ✓ |
| *Polydora websteri* |  |  | ✓ |  |  | ✓ |
| *Pseudopolydora paucibranchiata* |  |  | ✓ |  |  | ✓ |
| ***Pyromaia tuberculata*** | ✓ |  | ✓ | ✓ |  |  |
| ***Sabella spallanzanii*** | ✓ |  |  | ✓ |  |  |
| *Schizoporella spp.* | ✓ |  |  |  |  |  |
| ***Styela clava*** | ✓ |  |  | ✓ | ✓ |  |
| *Styela plicata* |  |  | ✓ |  | ✓ |  |
| *Thecacera pennigera* |  |  |  | ✓* |  |  |
| ***Theora lubrica*** | ✓ |  |  | ✓ |  |  |
| ***Tritia burchardi*** | ✓ |  |  | ✓ |  |  |
| Total species (non-indigenous species detected) | 17 | 18 | | 18 | 17 | |
| Shared with NMHRSS (metabarcoding only) | — | 3 | | — | 2 | |
| Shared between years (NMHRSS only) | — | — | | 10 | — | |

**Table Notes**: †Although not detected in the NMHRSS Annual Report 2023–2024, these species are known to be established in New Zealand waters. *Indicates range extensions in the NMHRSS, i.e. new detections in Ōpua Marina/Waikare Inlet not previously recorded during the NMHRSS survey. Bold indicates species noted in both NMHRSS reports. If metabarcoding/NMHRSS reports could only resolve to the genus level (spp.), then the genus and its associated species are grouped together (by bar colour) to indicate that the genus-level detection could correspond to one of the listed species.
