# Supplementary material for: Automated eDNA sampling for marine monitoring and biosecurity: optimising temporal resolution, remote deployments, and community engagement
Source: PeerJ. 2026 May 28;14:e21287. doi: 10.7717/peerj.21287 (PMC13222548; doi:10.7717/peerj.21287)
Supplement: Supplemental Information 2 [file peerj-14-21287-s002.docx]

**Smith-Root eDNA Autosampler Service Protocol**

**Opua deployment 1 Dec 2023 – 10 Jan 2024**

| **Autosampler field service schedule:**  01 Dec 2023 12:00pm: Set-up  09 Dec 2023 12:00pm: field service (recharge)  17 Dec 2023 12:00pm: field service (recharge)  25 Dec 2023 12:00pm: field service (recharge)  2 Jan 2024 12:00: field service (recharge)  10 Jan 2024 (or later): device collection by Sequench | | |
| --- | --- | --- |
| 1. | Upon arrival on-site, inspect the device (ensure the intake/outlet lines are not misplaced, no visible damage to the device observed). Take notes on relevant environmental conditions, nature state, etc. | 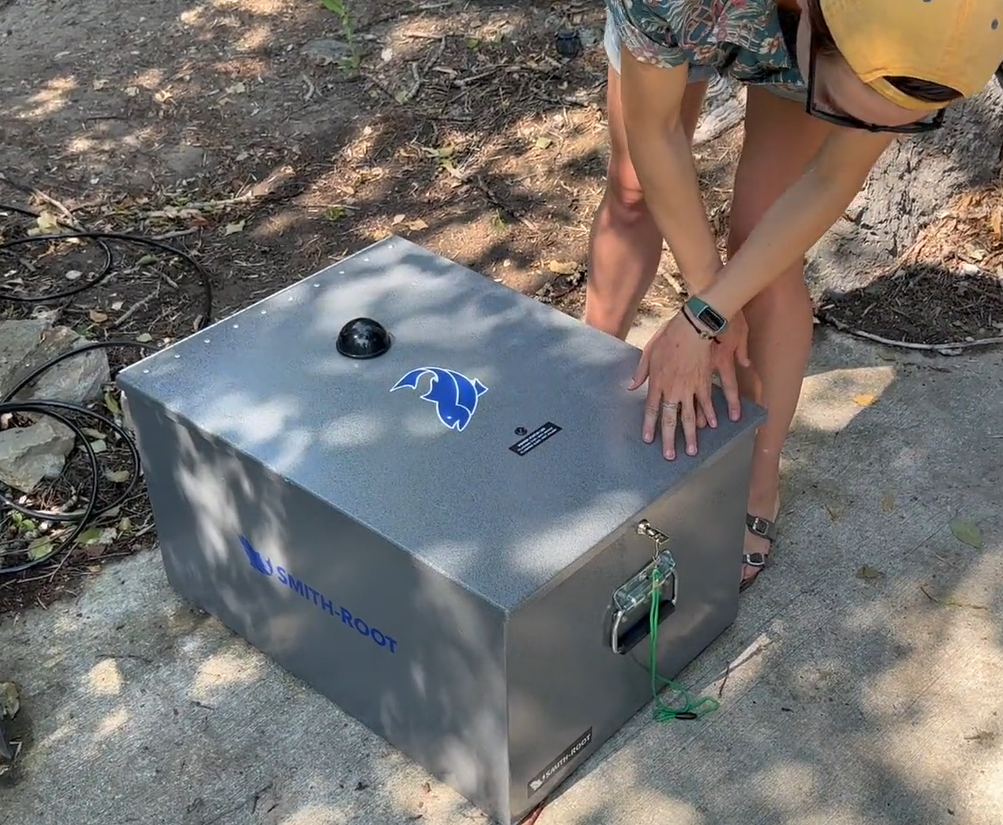 |
| 2. | Retrieve the strainer on the submerged end of the intake line from water. Brush it carefully with the brush provided in the field box to remove any attached debris, rinse in the ambient water. Place the used strainer in a plastic container for follow-up bleaching. |  |
| 3. | Put on a fresh set of single-used gloves. Take out a clean intake strainer from the plastic bag, attach the strainer by firmly pressing one end of the intake tubing into the connection fitting. Press in and then gently pull on the tubing several times to ensure that the tubing is fully seated. Submerge the intake line with attached strainer into water at the designated depth (ensure it is not hitting the bottom). | 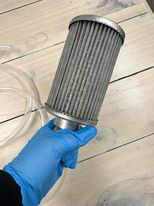 |
| 4. | Unlock the System using the key provided and open the lid. Press the Wake/Sleep button to wake up the System. Note the sample volumes for each filter and check for any unexpected outcomes. Record these in the logbook and/or download the data using the USB key. | 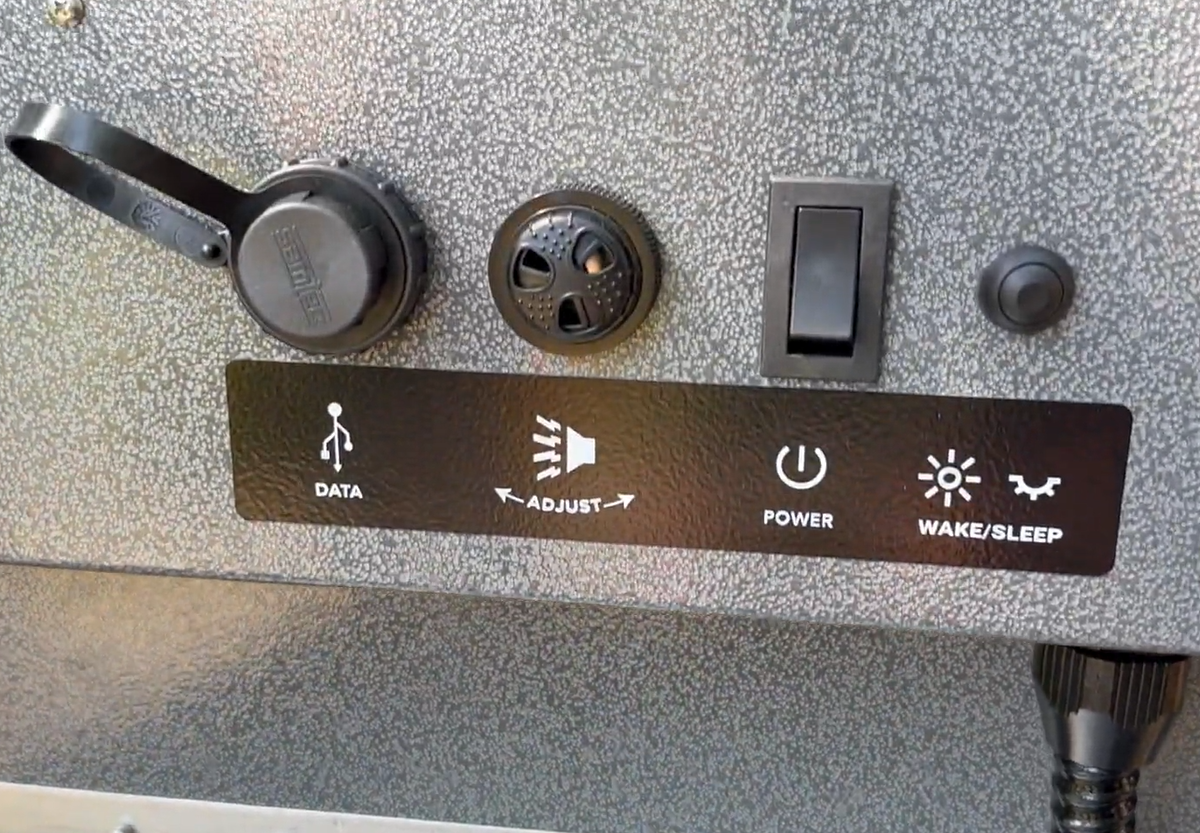 |
| 5. | For each sample that will be collected there should be an empty sample bags in the field box (these are the same bags that the filters came out of and were retained after filter installation). Label each pouch for the samples to be collected using the information provided on the screen. For example: **Sample A1-00345; Date 7/8/23, 14:30, 1.9 L** | 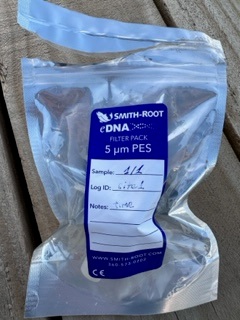 |
| 6. | Remove the 6 manifold bolts and place them in the retaining tray. **DO NOT operate the manifold lift when the bolts are installed**. | 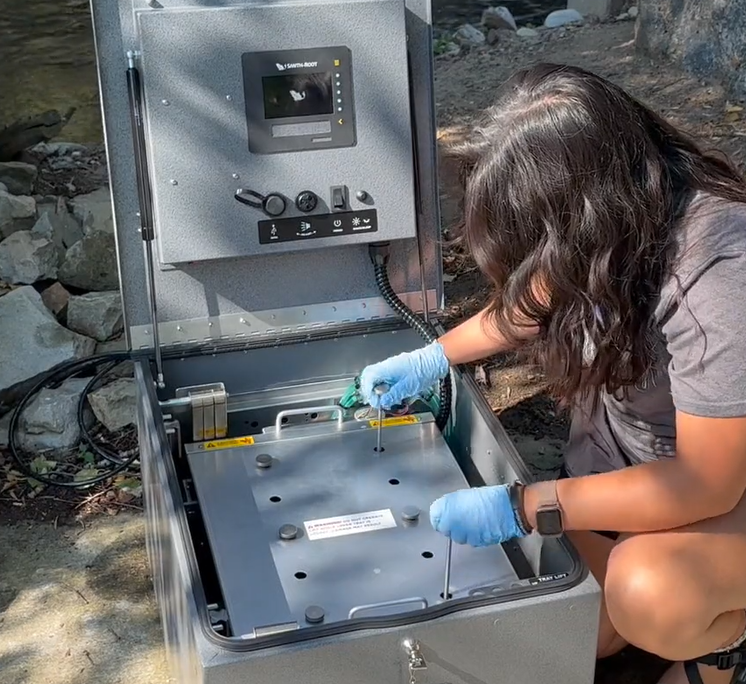 |
| 7. | Activate the electric lift to disengage the top manifold. Stop when the two manifolds separate. Gently lift up on the manifold using the handles and push away until the manifold comes to a rest in a vertical position, exposing the lower manifold and filters. | 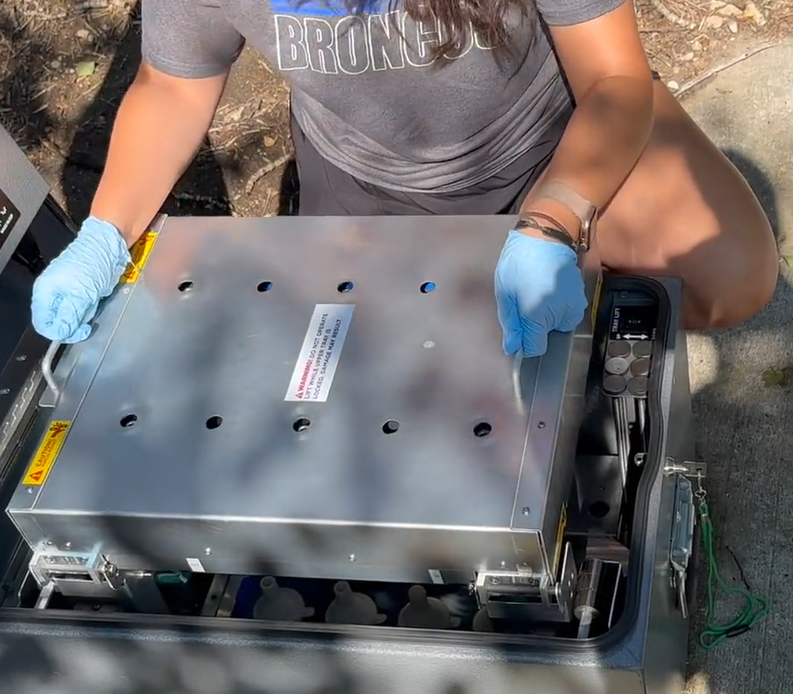 |
| 8. | Put on a fresh pair of single-use gloves. Using a clean paper towel, absorb visible water drips on the outside of the filter housings or on the lower manifold. Avoid touching the open tops of the filter housings to minimize risk of contamination. If any of the filters are stuck in the upper manifold, remove them by grabbing the barbed end and gently rotating/pulling the filter until it comes free. Use mild force, this reduces the likelihood of separating the upper and lower housings which will expose the filter. Place the filter back in its appropriate location in the lower manifold. |  |
| 9. | Starting with filter A1, slide fingers under the filter housing and lift straight up to remove it from the manifold – two hands may be necessary. Avoid placing hands directly over the open nozzle of the filter housing to minimize risk of contamination. Place the filter into the appropriate pre-labeled bag. Minimize air volume in the bag and carefully seal it shut, making sure that the zip-top seal is completely closed. Place the sample in a storage container. | 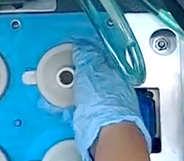 |
| 10. | Once all filters have been collected and bagged, carefully disconnect the used battery and attach the new charged one. | 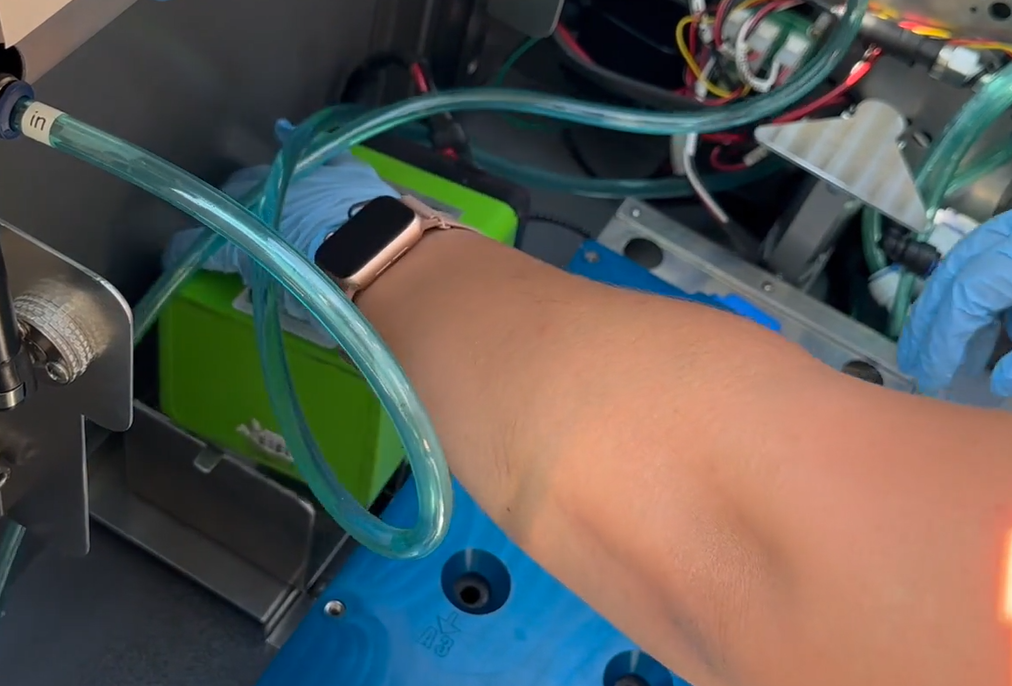 |
| 11. | Proceed to loading the manifold with fresh filters. Put on a fresh set of single-use gloves, open a new Smith-Root self-preserving filter packet, remove the filter and press it firmly into place in the manifold. Make sure that the filter tab is directionally oriented with the arrow etched into the manifold. Continue until all 8 filter locations are loaded. | 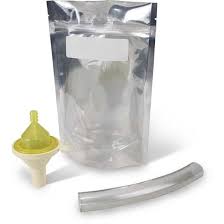 |
| 12. | Reset the top manifold by articulating it into position and pressing gently downward until it makes contact with the lower manifold. **Ensure fingers and pipes are clear of the manifold hinge before articulating the manifold**. Ensure that the electric lift is fully in the down position.  Place the manifold bolts back into position. Loosely tighten each bolt initially, before fully tightening any single bolt. If it is difficult to align the bolts, gentle downward pressure can be applied to the top manifold with the free hand to assist with threading the bolt.  When the manifold is loaded and secure, proceed to Programming the System. | 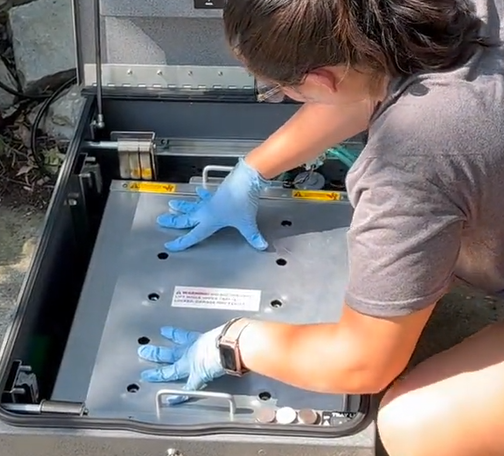 |
| 13. | Click on the Menu button (3 horizontal lines outlined on the Figure).  Select **New Sample Run**. | 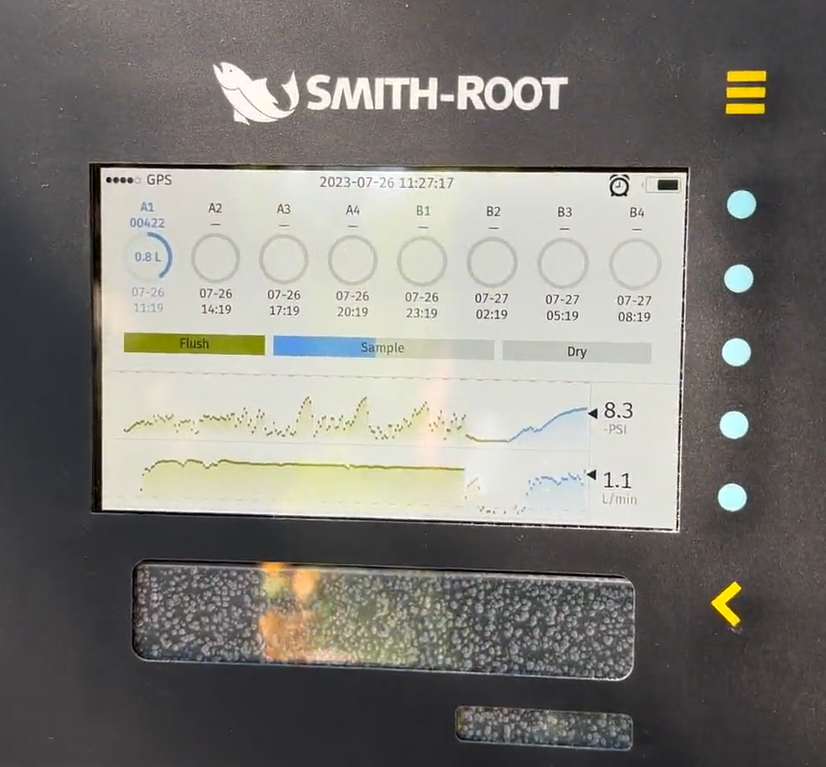 |
| 14. | On the ‘Schedule’ page check that correct settings are selected:  **Number of samples 8**  **Time between samples 24 hours (1,440 min)**  NOTE: the first sample is taken on filter A1 immediately after set-up, so try to attend the device around the same time of the day to maintain consistency in sampling schedule.  On the ‘Settings’, check the following:  **Target Volume 4.0 L**  **Target Flow 1.0 L**  **Maximum Pressure 10.0 PSI**  **Flush Volume 2 L** |  |
| 15. | If everything is correct, go to ‘Start’ page and press **Start.** Observe the first sample. Verify pressure, flow, and volume targets are met. Lock the box with the key. | |
