# Supplementary material for: Automated eDNA sampling for marine monitoring and biosecurity: optimising temporal resolution, remote deployments, and community engagement
Source: PeerJ. 2026 May 28;14:e21287. doi: 10.7717/peerj.21287 (PMC13222548; doi:10.7717/peerj.21287)
Supplement: Supplemental Information 3 [file peerj-14-21287-s003.pdf]

eDNA AUTOSAMPLER

USER MANUAL

v1.0

SMITH-ROOT

www.smith-root.com/support
info@smith-root.com
1-360-573-0202
16603 NE 50th Ave • Vancouver, WA 98686 USA

Contents

1 Safety & Warnings **1**
1.1 Safety 1
1.2 Warnings 1
1.2.1 Battery Warnings 1

2 Overview **2**
2.1 Components 3

3 Setup **4**
3.1 Select a Location 4
3.2 Install 5
3.3 Secondary Enclosure 6

4 Basic Operation **7**
4.1 Display 7
4.2 Open Manifold 8
4.3 Close Manifold 8
4.4 Connect Tubing 9
4.5 Disconnect Tubing 9

5 The Sampling Process **9**
5.1 Load Manifold 9
5.2 Schedule a Sample Run 11
5.3 Recover Samples 12
5.4 Download Logs 14

6 Advanced Operation **15**
6.1 Cancel a Sample Run 15
6.2 Check In on a Sample Run 15
6.3 Filter Clog Point 16
6.4 Prime Time 17
6.5 Advanced Scheduling 17
6.5.1 Create Schedule File 17
6.5.2 Load Schedule File 18
6.6 Collect a Field Blank 18
6.7 Other Features 19
6.7.1 Clock & UTC Offset 19

6.7.2 Power LED 20
6.7.3 Beeper 20

7 Maintenance **20**
7.1 Battery 20
7.1.1 Charge Battery 20
7.1.2 Remove Battery 21
7.1.3 Install Battery 21
7.1.4 Battery Disposal 21
7.2 Cleaning 21
7.3 Sterilize 21
7.4 Update Software 23
7.5 Storage 23
7.6 Battery Storage 24
7.7 Sample Storage & Transport 24

8 Troubleshooting **24**
8.1 Diagnostics 24
8.2 Manual Control 26
8.2.1 Flow Diagram 27
8.3 Common Issues 27
8.3.1 Unable to achieve pressure 27
8.3.2 Excess moisture in unit 27
8.3.3 Can’t download logs 28
8.3.4 Filter stuck in upper manifold 28
8.3.5 Premature clogging 28

9 Specifications **28**
9.1 Technical Specifications 28
9.2 Log File Format 29
9.2.1 Header Section 30
9.2.2 Data Section 31
9.3 The Autosampling Process 32

Index **33**

Symbol	Description
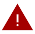	Action may result in injury or damage.
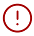	Action may result in suboptimal results.
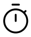	Information on how long an action takes.
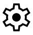	Description of an automated process.
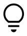	Tips & tricks.

1 Safety & Warnings

1.1 Safety

- ⚠ Keep fingers and tubing clear when articulating the manifold.
- ⚠ Two-person lift required. Equipment exceeds 100 lbs.
- ⚠ Do not move with tubing attached.
- ⚠ Obtain permission from property owners before installing.
- ⚠ Secure tubing so that it does not pose a trip hazard.
- ⚠ Be aware of trip/slip hazards and uneven terrain.

1.2 Warnings

- ⚠ Do not operate tray lift with bolts installed. Damage *will* occur.
- ⚠ Do not operate in freezing temperatures.
- ⚠ Do not allow debris in the lower manifold openings.
- ⚠ Do not operate without intake strainer.

1.2.1 Battery Warnings

Product contains a Lithium Iron Phosphate ($LiFePO_4$) battery.

- ⚠ Do not short circuit battery leads.
- ⚠ Do not drop battery.
- ⚠ Keep battery temperature within -20–56°C.
- ⚠ Do not submerge battery.
- ⚠ Only charge with provided charger or AC cord.
- ⚠ Do not use battery if it has been damaged in any way.

2 Overview

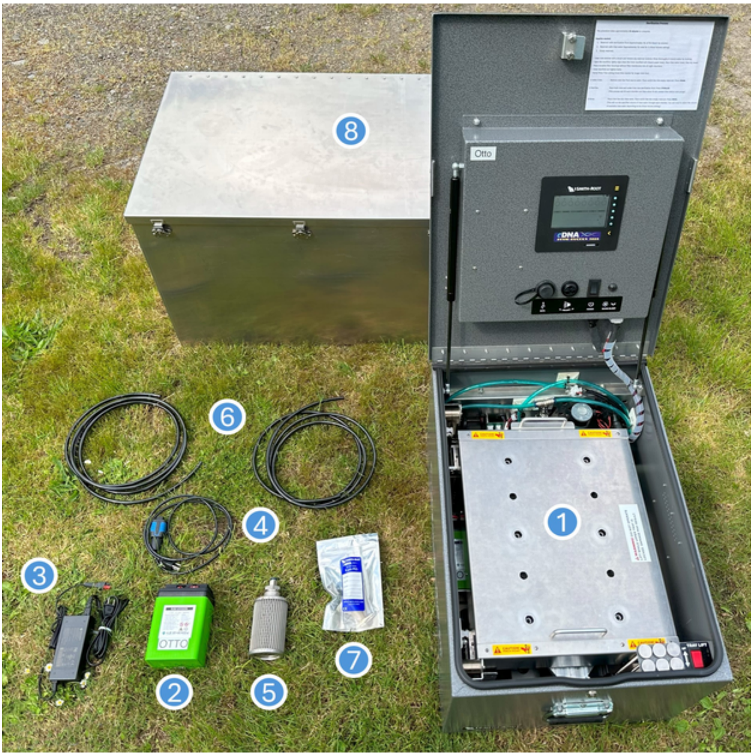

Figure 2.1: Components provided by Smith-Root

1. *e*DNA Autosampler (1)
2. Battery (1)
3. External battery charger (1)
4. AC power cable (1)
5. Intake strainer (1)
6. Tubing (100′)
7. Filterless nylon housings (8)

Additions items in the combo package:

8. Shipping case (1)
9. Additional battery (1) *(not shown)*
10. Additional intake strainer (1) *(not shown)*

2.1 Components

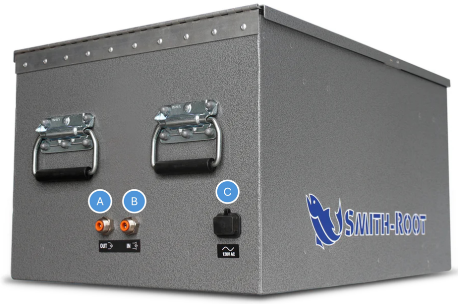

Figure 2.2: Rear

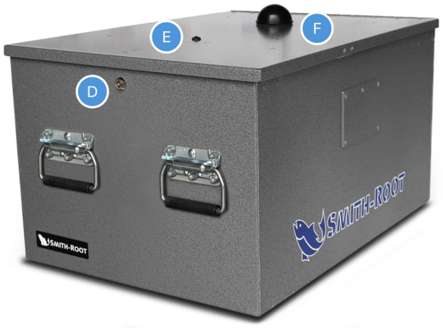

Figure 2.3: Front

A. Out port
B. In port
C. AC power inlet

D. Lock
E. Power LED
F. GPS antenna

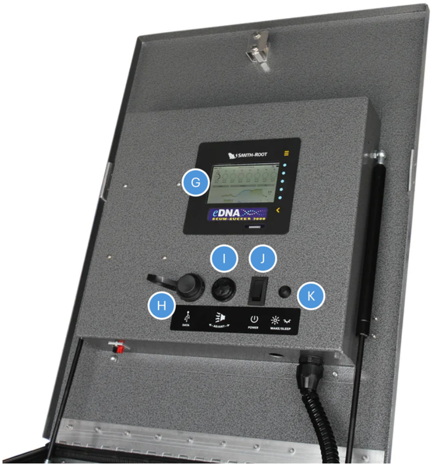

Figure 2.4: Control Panel

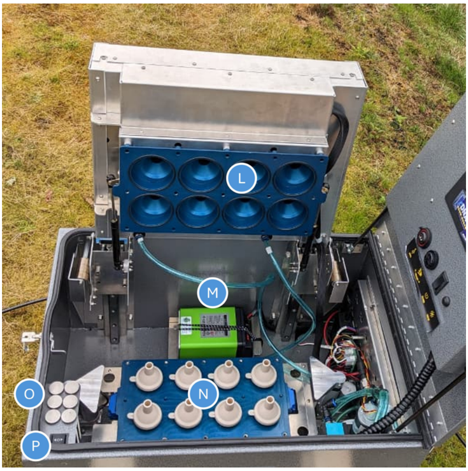

Figure 2.5: Open manifold

G. Control panel
H. USB data port
I. Beeper
J. Power switch
K. Sleep/wake button

L. Upper manifold
M. Battery tray and connector
N. Lower manifold
O. Manifold bolt retaining tray
P. Tray lift switch

3 Setup

3.1 Select a Location

- Locate the unit in a stable area with minimal direct sun exposure.
- Locate the intake strainer in an area with moderate flow not subject to high current velocities.
- Keep lift height under 2 meters. Large lift heights reduce filtration pressure.

ⓘ *Do not cover unit with tarp or plastic sheeting.*

ⓘ *Do not place intake strainer directly on the substrate (sediment or otherwise).*

💡 *Use an external enclosure if location is in direct sunlight, See 3.3 Secondary Enclosure (page 6).*

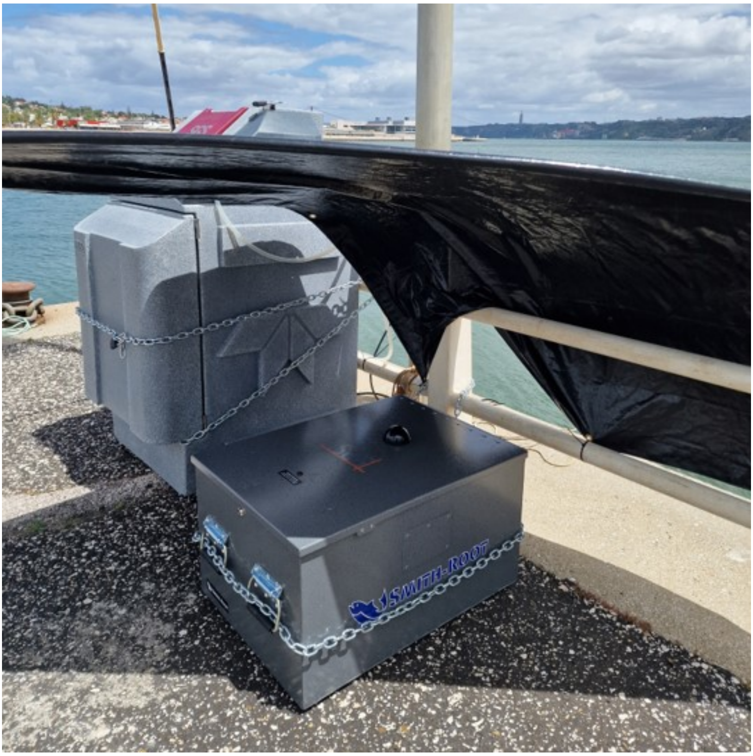

Figure 3.1: Installation location

3.2 Install

Supplies:

- Scissors or tubing cutter
- Zip ties
- Diagonal cutters
- Rebar or stake
- U stakes
- Mallet

Procedure:

1. Cut inlet and outlet tubing to length. Allow a bit of excess.
2. Attach one end of the inlet tube to the intake strainer, and the other end to the IN port. See section 4.4 CONNECT TUBING (page 9).
3. *(Optional)* Secure the intake strainer.
   (a) Drive rebar/stake into substrate with mallet.
   (b) Secure intake strainer to rebar/stake with zip ties. (Figure 3.2.)
   (c) Trim zip ties with diagonal cutters to minimize potential for catching debris.
4. Secure inlet tubing along the bottom of the water body and along the ground with U-stakes, additional rebar, or small rocks.

⚠ *Do not create pinch points or tripping hazards.*

5. Attach one end of the outlet tube to the OUT port, and position the open end in a location that can receive drainage water. See section 4.4 CONNECT TUBING (page 9).

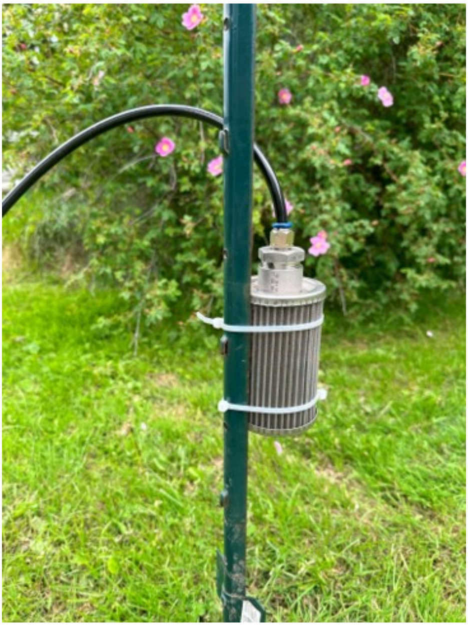

Figure 3.2: Strainer install

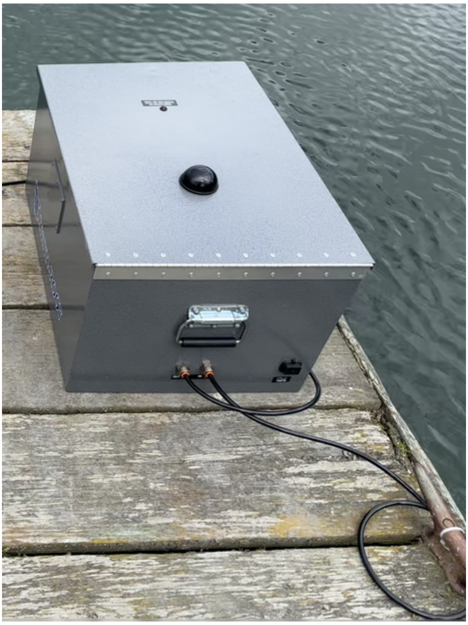

Figure 3.3: Tubing looped and secured

3.3 Secondary Enclosure

Use a secondary enclosure when installing in direct sunlight. The *e*DNA Autosampler enclosure is weather resistant, however, direct sun exposure may result in high temperatures that damage the preserved DNA. If possible, install in a shaded location.

Supplies:

- Cordless drill and 1/2" drill bit
- Secondary enclosure
  - *One suitable option:*
    Rubbermaid Model: FG374801OLVSS
    2' 7" × 5' Horizontal Resin Storage Shed

Installation procedure:

1. Assemble enclosure according to the manufacturer's instructions.
2. Drill two 1/2" holes for the inlet and outlet tubing.

For additional protection against chaffing, install an electrical conduit coupler.

3. Lock shed with padlock.

4 Basic Operation

4.1 Display

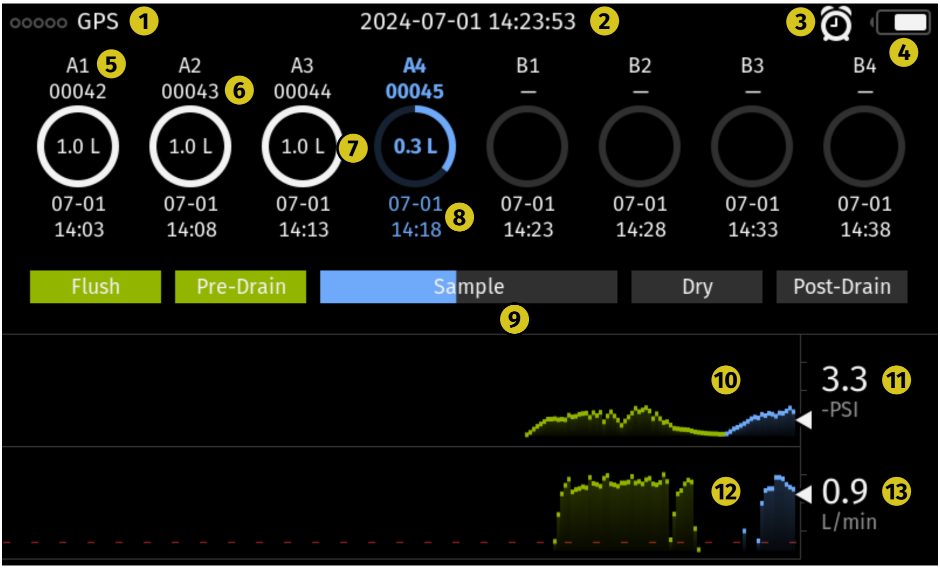

Figure 4.1: Main display view

1. GPS signal strength.
2. Clock.
3. Schedule active indicator – when a schedule is running, when no schedule is running.
4. Battery life.
5. Manifold location ID.
6. Sample ID – Uniquely identifies sample data. Auto-increments every time a new sample starts.
7. Sample volume – The volume of water passed through this filter membrane. The outer ring fills completely when TARGET VOLUME is achieved.
8. Sample start time.
9. Sample progress – Displays the status of the current sample, broken down by stage. Green indicates a flush/dry stage, blue indicates

sampling. For more details see 9.3 THE AUTOSAMPLING PROCESS (page 32).

10. Flow profile – Profile of the last ∼ 3.5 minutes of flow readings.
11. Flow readout.
12. Pressure profile – Profile of the last ∼ 3.5 minutes of pressure readings.
13. Pressure readout.

4.2 Open Manifold

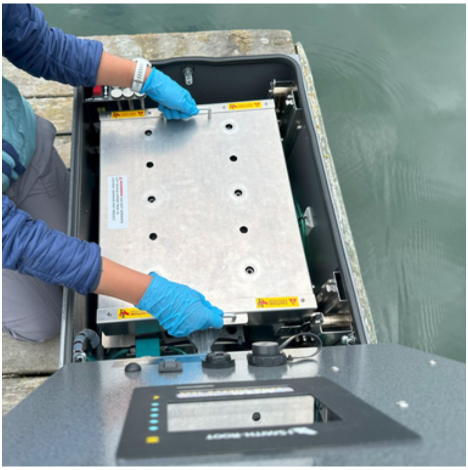

Figure 4.2: Lift

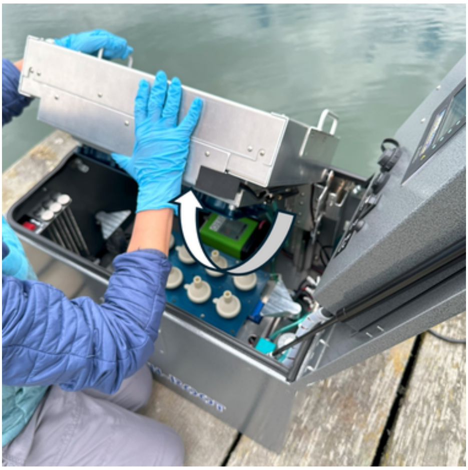

Figure 4.3: Articulate

To open the manifold:

Do not operate tray lift with bolts in place.

1. Loosen manifold bolts in a star pattern (figure 4.4).
2. Remove the 6 manifold bolts and place in retaining tray.
3. Press and hold tray lift switch. Release when manifolds separate.

The tray lift lowers automatically when the switch is released.

4. Lift the upper manifold by the handles until it articulates to a stop in a vertical position, exposing the lower manifold. (Figures 4.2 and 4.3.)

4.3 Close Manifold

To close the manifold:

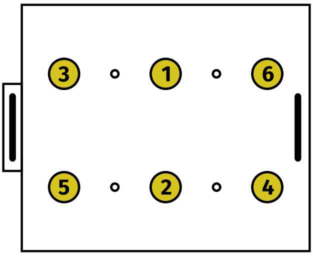

Figure 4.4: Bolt tightening/loosening star pattern

1. Close the top manifold by articulating it into position and pressing gently downward until it makes contact with the lower manifold.
2. Install manifold bolts.
   - *If it is difficult to thread the bolts, apply gentle downward pressure with your free hand.*

   (a) Loosely tighten each bolt in a star pattern (figure 4.4).

   (b) Continue tightening little-by-little around and around in a star pattern (figure 4.4) until all bolts are hand-tight.
   - *Do not use tools. Hand-tighten only.*

4.4 Connect Tubing

To connect tubing:

- *If end of tubing is damaged, cut and use a new section of tubing.*

1. Press tubing end firmly into fitting.
2. Pull gently to ensure tubing is fully seated.

4.5 Disconnect Tubing

To disconnect tubing:

1. Press and hold the orange release collar in towards the fitting.
2. Pull the tubing out of the fitting.

5 The Sampling Process

5.1 Load Manifold

Supplies:

- Smith-Root self-preserving filters (1–8)

💡 *For best results, use a filter pore size of 5µm or larger.*

- Single-use gloves (1)

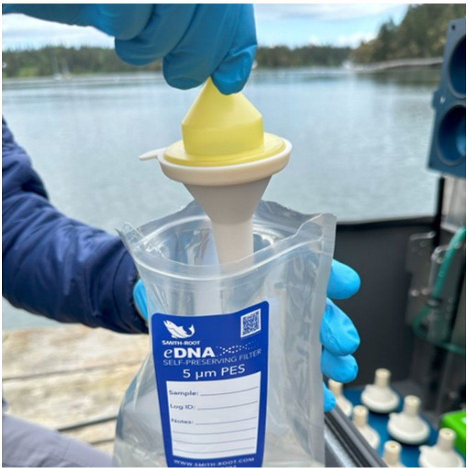

Figure 5.1: Open pouch

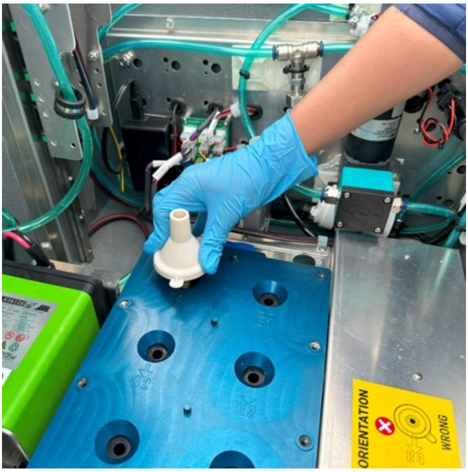

Figure 5.2: Install filter

Procedure:

1. Open the manifold. See section 4.2 OPEN MANIFOLD (page 8).
2. Put on a fresh pair of single-use gloves.
3. Remove a Smith-Root self-preserving filter from its packet by grabbing the barbed end (figure 5.1).

ⓘ *Do not place hands directly over the open nozzle of the filter housing to minimize risk of contamination.*

4. Push and twist filter housing firmly into lower manifold at position A1 (figure 5.2).

⚠ *Ensure the filter is fully seated and tab is oriented in the direction of the arrow etched in the manifold.*

5. Reseal the filter packet and save.
6. Continue loading filters in sequential order from A1 through B4 until the desired number of filters are loaded.
7. Close the manifold. See section 4.3 CLOSE MANIFOLD (page 8).

5.2 Schedule a Sample Run

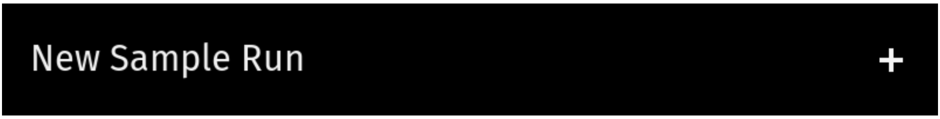

Figure 5.3: New Sample Run

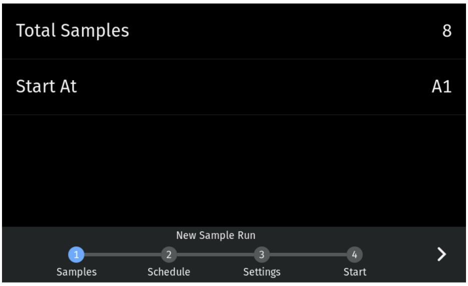

Figure 5.4: Page 1

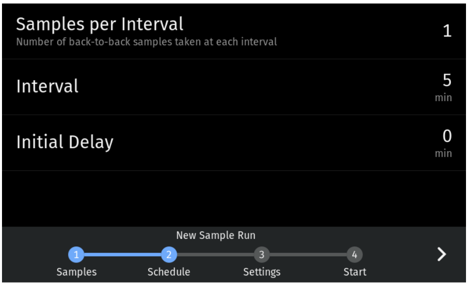

Figure 5.5: Page 2

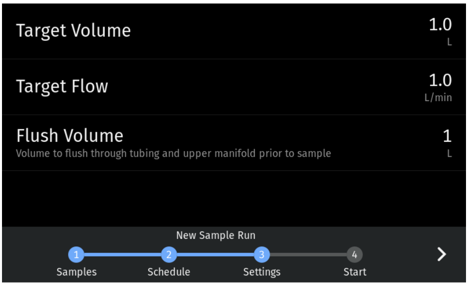

Figure 5.6: Page 3

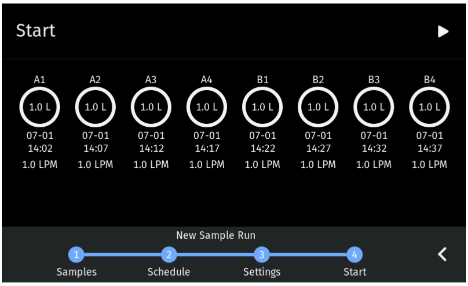

Figure 5.7: Page 4

To start a new run of samples:

1. Load filters following directions in section 5.1 LOAD MANIFOLD (page 9).
2. Inspect the intake strainer and clean if necessary. See section 7.2 CLEANING (page 21).
3. Select ≡ → NEW SAMPLE RUN.

⚙ *You are presented with the new sample run dialog (figure 5.4).*

4. On the SAMPLES page (figure 5.4):

   (a) TOTAL SAMPLES – Enter total number of samples to take (1–8).
   (b) START AT – Enter manifold ID of the first sample (A1–B4). Samples are taken sequentially starting at this position.
5. On the SCHEDULE page (figure 5.5):
   (a) SAMPLES PER INTERVAL – Select number of samples to take in each group, where a group is a series of back-to-back samples. For evenly-spaced samples select 1.
   (b) INTERVAL – Enter time between groups.
   (c) INITIAL DELAY – Enter time before the first sample.
6. On the SETTINGS page (figure 5.6):
   (a) TARGET VOLUME – Enter desired volume of water to pass through each filter.
   (b) TARGET FLOW – Enter desired rate of flow at which to take each sample.
   (c) FLUSH VOLUME – Enter amount of water flushed through the unit prior to each sample. This water is *not* passed through the filter.
7. On the START page (figure 5.7):
   (a) Review the schedule.
   Press < to revisit previous pages.
   (b) Press START ▶ to begin sampling.
   Do not turn the main power switch off.
   The unit goes to sleep in 30 seconds—unless a sample is scheduled soon.

5.3 Recover Samples

Supplies:

- Marker
- Filter pouches (saved from when the filters were opened)
- Single-use gloves
- Ziploc bag filled with fresh paper towels

Procedure:

1. Unlock and open the lid.
2. Turn on the POWER switch, or press the WAKE/SLEEP button if unit is asleep.
3. Pre-label each pouch using data on the display. For example:

- SAMPLE: 00345 - A1
- NOTES: 7/8/23, 14:30, 1.9 L

ⓘ *At minimum, label the 5-digit sample ID so that samples can be matched with log data.*

4. Open the manifold. See section 4.2 OPEN MANIFOLD (page 8).
5. Put on a fresh pair of single-use gloves.

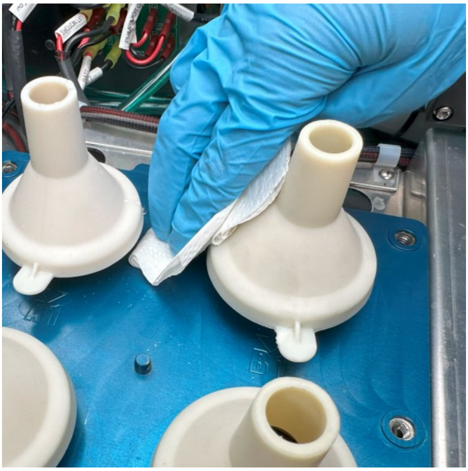

Figure 5.8: Wipe drops

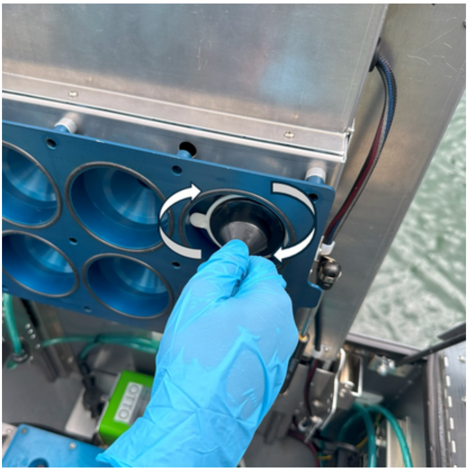

Figure 5.9: Stuck filter

6. Use a clean paper towel from a sealed container to wipe visible water drops Place used paper towel in field waste container. (Figure 5.8.)

ⓘ *Do not place hands directly over the open nozzle of the filter housing to minimize risk of contamination.*

💡 *If any filters are stuck in the upper manifold, grab the barbed end and gently rotate/pull until the filter comes free. Use mild force, as to not separate the upper and lower housings and expose the filter. (figure 5.9.) Place the filter back in its appropriate location in the lower manifold.*

7. Starting with filter A1, slide fingers under the filter housing and lift straight up to remove from manifold. (Figure 5.10.)
8. Place filter back in its original pouch that was pre-labeled in step 3. Minimize air volume in bag and completely seal the zip-top.
9. Continue until all filters are bagged.

ⓘ *Do not place filters in direct sunlight. Temperature-regulated storage is not necessary unless extreme heat is expected. See section*

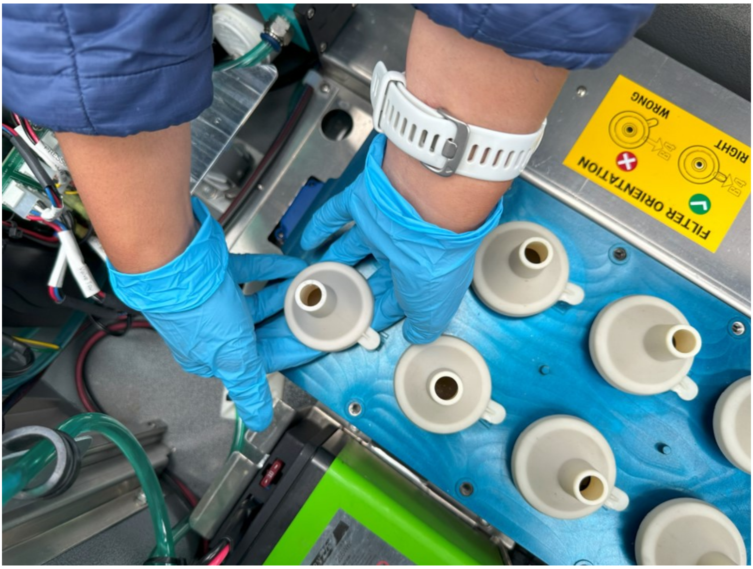

Figure 5.10: Remove filter

7.7 Sample Storage & Transport (page 24).

5.4 Download Logs

Retrieve logs with a FAT32-formatted USB thumb drive.

To retrieve logs:

1. Insert drive into the USB data port.
   You are presented with a time frame selection menu.
2. Select Day, Week, Month, Year, or All.
   Transfer time depends on unit usage and the time frame selection. More than 60 seconds is abnormal.
3. Remove drive when prompted.

For a description of the log files see section 9.2 Log File Format (page 29).

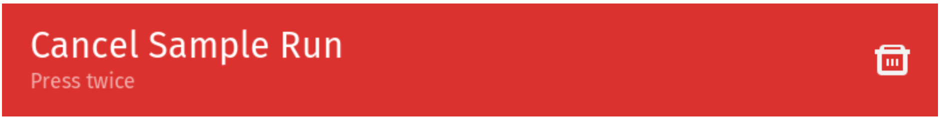

Figure 6.1: Cancel Sample Run

6 Advanced Operation

6.1 Cancel a Sample Run

To cancel a sample run:

1. Select ≡ → Cancel Sample Run.

Only available when a sample run is in-progress.

2. Press twice to confirm.

Cancelling a run may leave unused filters in the manifold. These filters may be utilized without unsealing the manifold by starting a new sample run with the Start At setting set to the position of the first unused filter. See section 5.2 Schedule a Sample Run (page 11). For example: if an 8 sample run is canceled after the 3rd sample, the run may be continued by starting a new sample run with Start At set to A4 and Total Samples set to 5.

6.2 Check In on a Sample Run

Waking the unit up in the middle of a sample run does not interfere with its schedule. If the unit happens to be awake at a scheduled sample time, the sample is automatically taken as usual. When in-between samples, a banner appears at the bottom of the display that indicates the amount of time until the next sample (figure 6.2).

To check-in on a sample run:

1. Press Wake/Sleep to wake the unit up.
2. View the main screen logs.
3. Press Wake/Sleep to put the unit back to sleep.

Do not turn the main power switch off.

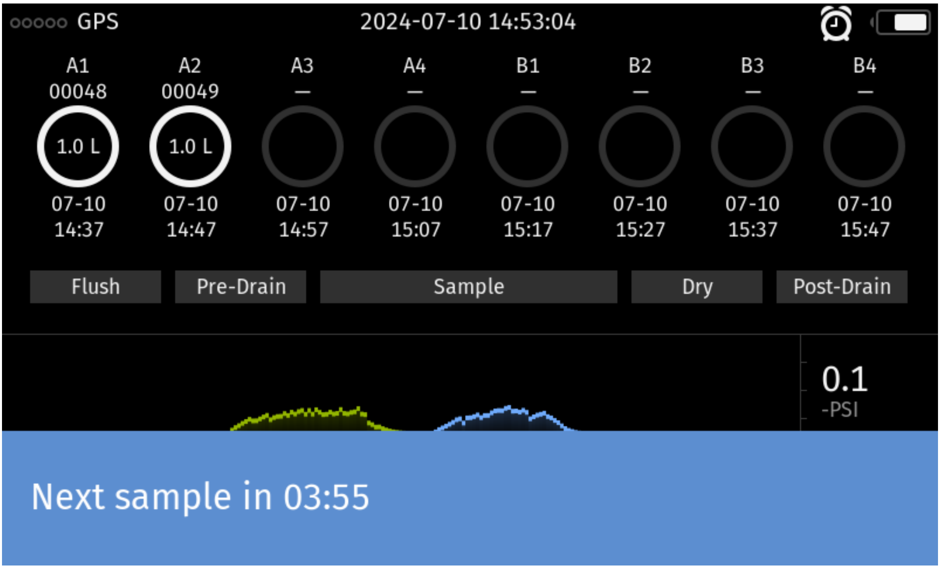

Figure 6.2: In-between samples

6.3 Filter Clog Point

To prevent clogging, a sample aborts when flow falls below a set point. Small values ensure target volume is achieved, large values promote better filter drying. Ideal value is as large as possible, but not so large that target volume is not achieved.

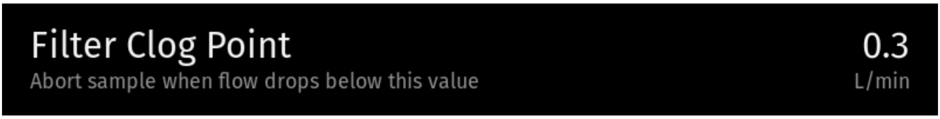

Figure 6.3: Filter Clog Point

To adjust filter clog point:

1. Select ≡ → FILTER CLOG POINT.
2. Enter a value with the ∧ / ∨ buttons.
3. Press ‹ to close the edit pane.

6.4 Prime Time

To prevent the automatic sampling process from getting "stuck," a sample aborts when no water is drawn for a timeout period. Small timeouts allow closer back-to-back sampling, large timeouts accommodate longer tubing.

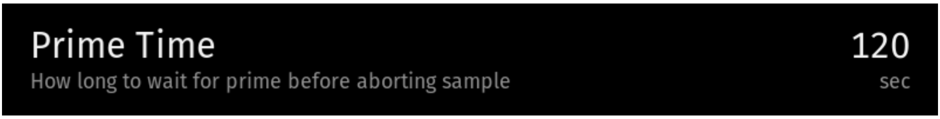

Figure 6.4: Prime Time

To adjust prime time:

1. Select ≡ → PRIME TIME.
2. Enter a value with the ∧/∨ buttons.
3. Press 〈 to close the edit pane.

6.5 Advanced Scheduling

Advanced scheduling supports arbitrary sample times and per-sample settings for target volume, target flow, and maximum pressure. If these features are not required, use the standard scheduler as described in section 5.2 SCHEDULE A SAMPLE RUN (page 11).

To use the advanced schedule feature, create a schedule file on a PC and then transfer it to the unit via a USB thumb drive.

6.5.1 Create Schedule File

To run an advanced schedule, you first need to create a CSV (Comma Separated Value) text file on a PC with a row for each sample event (up to 8 rows), and 5 columns with the following column definitions:

1. FILTER ID – Manifold location (A1–B4).
2. UTC TIME – Sample time.
3. TARGET VOLUME – Volume of water to filter. 0.5–20 Liters.
4. TARGET FLOW – Target flow rate. 0.2–14 L/min.
5. PRESSURE LIMIT – Maximum pressure. Pump is throttled to keep pressure below this limit, dropping below TARGET FLOW if necessary.

0.6–14.7 -PSI.

A header row with column descriptions is optional. When finished, name the file “schedule.csv”.

```
Filter ID,UTC Time,Target Volume,Target Flow,Maximum Pressure
A1,2023-01-01 12:00,0.8,0.7,12
A2,2023-01-02 12:00,1.0,0.7,12
A3,2023-01-03 12:00,1.2,0.7,12
A4,2023-01-04 12:00,1.4,0.7,12
B1,2023-01-05 12:00,1.8,0.7,12
```

Figure 6.5: CSV schedule file example (with header row)

The example schedule file in figure 6.5 defines five samples at manifold positions A1–B1 that sample daily at noon for five consecutive days using increasingly larger target volumes each day.

6.5.2 Load Schedule File

To load a CSV schedule file:

1. Insert thumb drive into PC.
2. Copy your CSV schedule file to the drive.
   ⓘ *Make sure the schedule file is named “schedule.csv”.*
3. Unmount and remove drive from PC.
4. Insert drive into the USB data port.
   The schedule is detected and loads automatically. You are presented with a confirmation screen.
5. Remove drive when prompted.
   ⓘ *Do not turn the main power switch off.*
   The unit goes to sleep in 30 seconds.

6.6 Collect a Field Blank

It is good practice to occasionally collect a field negative control to assess the potential for cross-sample contamination or other sources of field contamination.

Supplies:

- Smith-Root self-preserving 5µm filter (1)
- Reservoirs with openings large enough to insert the intake strainer (2)

- DNA-free water (12 L)
  - *Required volume depends on RINSE VOLUME and TARGET VOLUME settings. Typically around 12 liters.*

There are two methods for collecting a field blank: in a run of samples or in a separate run.

Method #1—Collect blank in a run of samples:

1. Take a sample run as usual, with the following exceptions:
   - Load an extra filter at position B4.
   - Program the sample run as usual—but stop short of position B4. (E.g. start at position A1 and take 7 samples.)
2. At the completion of the scheduled run:
   (a) Rinse debris from the intake strainer by removing the strainer from sampling waters and placing in the first reservoir of DNA-free water.
   Rinsing ensures dislodged debris does not clog the field blank.
   (b) Place intake strainer in the second reservoir filled with DNA-free water.
   (c) Start a new sample run that takes one sample at position B4. See section 5.2 SCHEDULE A SAMPLE RUN (page 11).

Method #2—Collect blank in a separate run:

1. After collecting samples from a run, load manifold with a single filter at location A1. See section 5.1 LOAD MANIFOLD (page 9).
2. Rinse debris from the intake strainer by removing the strainer from sampling waters and placing in the first reservoir of DNA-free water.
   Rinsing ensures dislodged debris does not clog the field blank.
3. Place intake strainer in the second reservoir of DNA-free water.
4. Start a new sample run that takes a single sample at position A1. See section 5.2 SCHEDULE A SAMPLE RUN (page 11).

6.7 Other Features

6.7.1 Clock & UTC Offset

The clock sets automatically via GPS and defaults to UTC time (i.e. Greenwich Mean). To operate in local “wall clock” time, adjust the UTC OFFSET setting to your local UTC time zone offset. In regions that observe daylight saving time, adjust this setting twice per year.

To automatically set the clock:

1. Place unit outdoors in a location with a clear view of the sky.
2. Power unit on.

⏱ *Wait for one or more GPS signal dots to appear.*

To adjust UTC offset:

1. Select ≡ → UTC OFFSET.
2. Enter your local time zone (-12–14).

6.7.2 Power LED

The power LED on top of the unit blinks to indicate when the unit is powered on or asleep. When awake the LED blinks fast (once per second), and when asleep it blinks slow (once every five seconds). The slow blink is brief and may be difficult to see in direct sunlight. Shade the LED with your hand for better visibility.

6.7.3 Beeper

The 🔈 beeper on the control panel beeps twice when the unit powers on or wakes up, and beeps three times when the pump starts or stops. Rotate the baffle to adjust volume.

7 Maintenance

7.1 Battery

7.1.1 Charge Battery

ⓘ Do not leave battery discharged for an extended period of time.

To charge in unit:

1. Connect to AC outlet using the provided AC cord.

To charge out of unit:

⚠ *Only charge with the provided Smith-Root charger.*

1. Remove battery. See section 7.1.2 REMOVE BATTERY (page 21).
2. Connect battery to the provided charger and plug into AC outlet.

7.1.2 Remove Battery

1. Open the manifold. See section 4.2 OPEN MANIFOLD (page 8).
2. Unplug battery connector.
3. Unclasp battery retention cord.
4. Remove battery.

7.1.3 Install Battery

1. Open the manifold. See section 4.2 OPEN MANIFOLD (page 8).
2. Insert battery into battery tray.
3. Clasp battery retention cord over battery.
4. Plug connector into battery.

7.1.4 Battery Disposal

All batteries are subject to disposal and recycling regulations that vary by country and region. Always check and follow your applicable regulations before disposing of any battery. Contact your local battery recycling organization or return to Smith-Root for recycling.

7.2 Cleaning

⚠ *Do not use solvent-based cleaners.*

1. Wipe the exterior with a soft cloth and a mild soap and water solution.
2. Scrub the intake strainer with a soft nylon brush.

7.3 Sterilize

⏱ *The sterilization procedure takes approximately 30 minutes.*

Supplies:

- Reservoirs (2)
  1. Sterilization fluid reservoir filled with 10 liters of a 5% bleach solution.
  2. Rinse water reservoir filled with ~12 liters, or 12 times the volume specified by the RINSE VOLUME setting (figure 7.3).
  3. Empty reservoir.

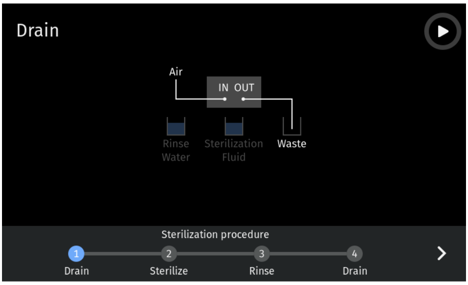

Figure 7.1: Pre-drain

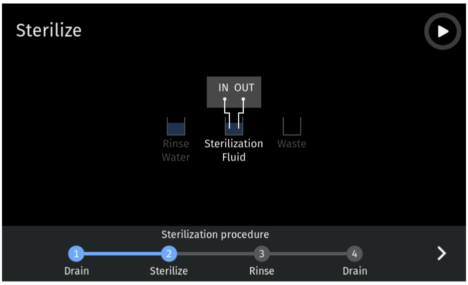

Figure 7.2: Sterilize

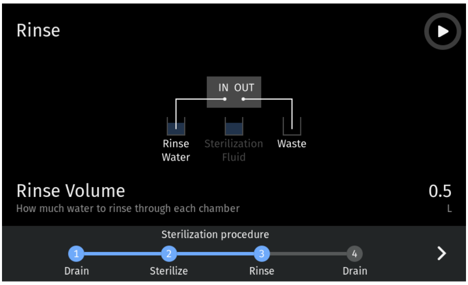

Figure 7.3: Rinse

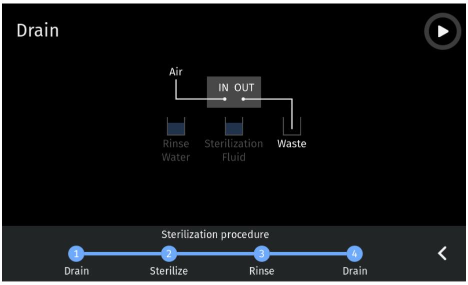

Figure 7.4: Final drain

- Low-lint cloths (2)

Procedure:

1. Open the manifold. See section 4.2 OPEN MANIFOLD (page 8).
2. Wipe manifold with a cloth dampened in sterilization fluid.
3. Wipe manifold with a cloth dampened in rinse water.
4. Dry manifold with a dry cloth.
5. Load reusable filter housings with no filter membranes into all eight chambers.

ⓘ *Ensure correct tab orientation.*

6. Close the manifold. See section 4.3 CLOSE MANIFOLD (page 8).
7. Power unit on.
8. Connect tubing to the IN and OUT ports. See 4.4 CONNECT TUBING (page 9).
9. Verify the PRIME TIME setting. See section 6.4 PRIME TIME (page 17).
10. Select ≡ → STERILIZE.
11. For each of the four pages of the sterilize dialog (figure 7.1), place the inlet and outlet tubes in the position indicated by the on-screen

diagram and press ▶ to run the cycle. The circle fills to indicate progress. When complete, press 〉 to go to the next cycle.
  - The DRAIN cycle removes water from through the lower manifold and each of the eight manifold positions.
  - The STERILIZE cycle fills the lower manifold and each of the eight manifold positions with sterilization fluid, waits 10 minutes, then drains the system.
  - The RINSE cycle flushes the system with a volume of water determined by the RINSE VOLUME setting (figure 7.3). Longer tubing requires a larger rinse volume.

12. Press 〈 to return to the menu screen.

Cycles may be ran individually or out of sequence.

7.4 Update Software

Contact Smith-Root to obtain a .psc update file.

Software update procedure:

1. On a PC, save the .psc update file to an empty, FAT32-formatted USB thumb drive.
2. Eject and remove the drive from the PC.
3. Insert the drive into the USB data port.

Updates take about 8 seconds to install.

4. Remove the drive when instructed.

The unit restarts with the new version.

7.5 Storage

- Sterilize prior to storage. See section 7.3 STERILIZE (page 21).
- Use provided caps on the IN and OUT ports when unit is not in use.
  - This insures adequate internal moisture.

  The pump may be damaged if it dries out.
- Charge battery prior to storage. See section 7.1.1 CHARGE BATTERY (page 20).
- Do not subject to freezing temperatures.

7.6 Battery Storage

Batteries slowly discharge during storage. Fully charge the batteries before storing. Recharge once every 6 months of storage at 20°C. Storing batteries at temperatures above 40°C may reduce their cycle life and increase their self-discharge rate. Batteries store better at low temperatures. Ideal storage temperatures are 0–4°C. Do not expose batteries to temperatures below -20°C. After storage, charge battery before use.

7.7 Sample Storage & Transport

Samples may be aggregated and stored at room temperature (70°F, 21°C) until bulk laboratory processing. Keep samples protected from sunlight at all times. Current data supports up to 6 months of preservation before DNA extraction. Samples may be shipped for processing—if concerned about temperature, include an ice pack in the shipping container.

8 Troubleshooting

8.1 Diagnostics

The diagnostics screen (figure 8.1) displays the status of all sensors.

To view the diagnostics screen:

1. Select ≡ → DEVICE INFORMATION → DIAGNOSTICS.
2. Press ‹ to return to the device information screen.

The following fields are displayed:

- DATE/TIME – UTC time.
- TEMPERATURE – Internal box temperature.
- GPS
  1. POSITION – Latitude and longitude.
  2. ALTITUDE
- MOTOR
  1. VERSION – Motor control board firmware version.
  2. BOARD REVISION – Motor control board hardware version.
  3. MAXIMUM PRESSURE – Maximum pressure setting.
  4. TARGET FLOW – Target flow setting.
  5. PRESSURE – Instantaneous pressure reading.

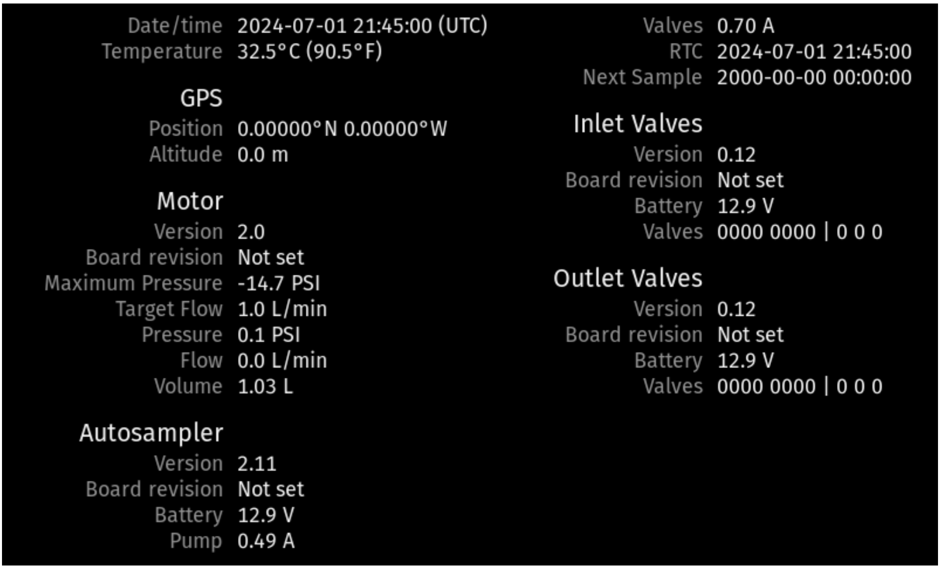

Figure 8.1: Diagnostics screen

6. Flow – Instantaneous flow reading.
7. Volume – Instantaneous volume reading.

- Autosampler
  1. Version – Autosampler board firmware version.
  2. Board revision – Autosampler control board hardware version.
  3. Battery – Battery voltage.
  4. Pump – Pump current draw.
  5. Valves – Cumulative valve actuator current draw.
  6. RTC – Real Time Clock (RTC) time in UTC.
  7. Next sample – Next sample time. Only set when a sample run is in-progress, otherwise, value reads "`2000-00-00 00:00:00`".
- Inlet/Outlet Valves (×2)
  1. Version – Valve board firmware version.
  2. Board revision – Valve board hardware version.
  3. Battery – Battery voltage reading.
  4. Valve – State of the valves on this board. 0 is closed, 1 is open. See figures 8.2 and 8.3.

```
0000 0000 | 0 0 0
|       |   | | └── Air 1
|       |   | └──── Water
|       |   └────── Purge
|       └────────── B4
|                   ...
└────────────────── A1
```

Figure 8.2: Inlet/upper valves

```
0000 0000 | 0 0 0
|       |   | | └── Unused
|       |   | └──── Unused
|       |   └────── Air 2
|       └────────── B4
|                   ...
└────────────────── A1
```

Figure 8.3: Outlet/lower valves

8.2 Manual Control

Manual control helps identify malfunctioning components. It is not necessary for normal operation. Refer to figure 8.5 FLOW DIAGRAM (page 27).

ⓘ *Do not open air valves while pump is off. Water may leak into case.*

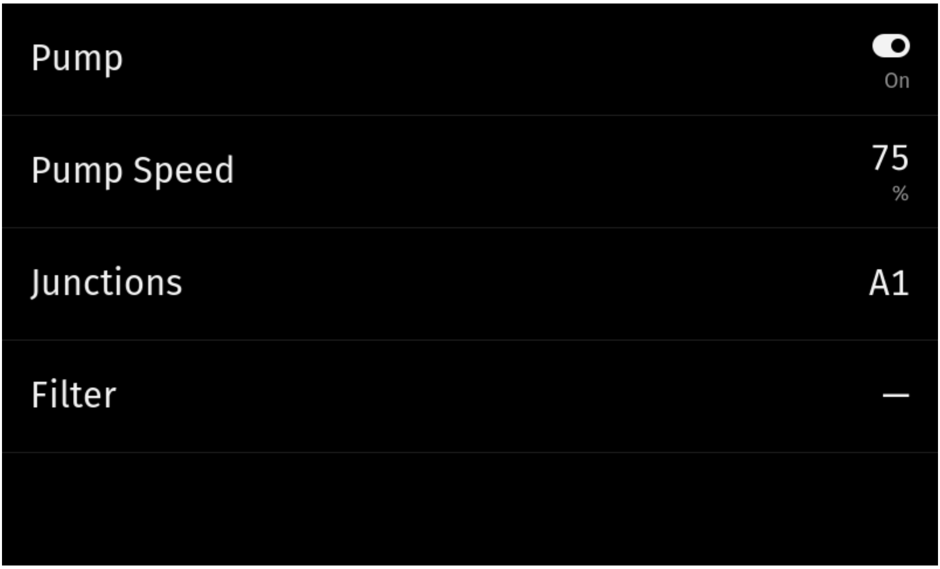

Figure 8.4: Manual control

To manually operate individual components:

1. Select ≡ → MANUAL CONTROL.
2. Use controls to manually operate the unit:
   (a) PUMP – Toggle pump on and off.

(b) Pump speed – Set pump speed, where 0% is (effectively) off and 100% is full speed.
(c) Junctions – Open/close the purge, water, air1, and air2 valves.
(d) Filter – Open/close the inlet and outlet valves for each manifold position. Use the spinner to select a manifold location, then use the toggles to select the inlet and outlet valve positions for that location.

3. Press ‹ to return to the menu screen.

⏱ *Settings remain in effect until power-cycled.*

8.2.1 Flow Diagram

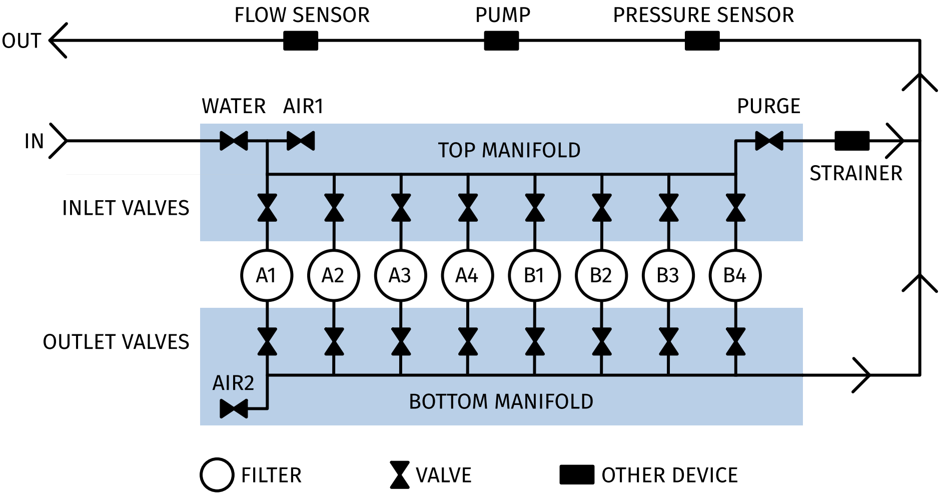

Figure 8.5: Flow diagram

8.3 Common Issues

8.3.1 Unable to achieve pressure

- Is the manifold is fully seated?
- Are the manifold bolts tight?
- Is the o-ring push connector for the intake tube fully sealed?

8.3.2 Excess moisture in unit

- Is there adequate ventilation around the side vent?

- A few drops on the manifold after a run is normal.
- Wipe pools with an absorbent towelette. Leave open to dry.

ⓘ *Do not cover unit with tarp or plastic sheeting.*

8.3.3 Can't download logs

- Is the drive full?
- Is the drive in FAT-32 format?
- Try another drive.

8.3.4 Filter stuck in upper manifold

- When loading, ensure filter housing is fully seated in lower manifold to prevent filters from getting stuck.
- Grasp the barbed end of the filter housing and gently twist and rock the filter housing.

ⓘ *Do not use force—filter housing may separate.*

8.3.5 Premature clogging

- Is the strainer touching the substrate of the water body?
- Decrease lift (distance between unit and water surface).
- Use a larger filter pore size.

9 Specifications

9.1 Technical Specifications

**General**	
Maximum Samples	8
Operable Filter Pore Size	$>$ 1.0µm ($\geq$ 5.0µm recommended)
Operable Lift	0–2 m
Enclosure Rating	Water resistant (IP 43)
**Sensors**	
Pressure Sensor Type	Transducer
Pressure Range	-14.5–30 PSI
Pressure Accuracy	±0.1 PSI
Flow Sensor Type	Paddlewheel

Flow Range	0.3–1.4 L/min
Flow Accuracy	±0.1 L/min
Volume Sensor Type	Derived from flow
Volume Range	0.2–999.9 L
Volume Accuracy	±80 mL per liter of target volume
**GPS**	
Compatibility	GNSS
	GPS, Galileo, GLONASS, BeiDou
Acccuracy	2.5 m
**Pump**	
Ports	3/8″ OD push-to-connect
Tubing	3/8″ OD 1/4″ ID polyurethane
	95 A durometer
Dry Vacuum	$\sim$ 10 PSI
Wetted Vacuum	$\sim$ 12 PSI
**Size & Weight**	
Length	31″ (787.4 mm)
Width	21″ (533.4 mm)
Height	15.5′ (393.7 mm)
Weight	94 lbs (42.6 kg)
	100 lbs (45.4 kg) with battery
**Power**	
Battery	22.5 Ah 12.8 VDC $LiFePO_4$
Max. battery deployment	30 days
AC input	85–264 V 48–63 Hz

9.2 Log File Format

Logs are saved to the drive in a directory named with the unit serial number. Files in that directory are named with the log ID and manifold location. (e.g. 00292_A2.csv is log ID 00292, taken at manifold location A2). Figure 9.1.

```
Drive
└── AB0123456
    ├── 00291_A1.csv
    ├── 00292_A2.csv
    ├── 00294_A3.csv
    └── ...
```

Figure 9.1: Log file listing

```
Serial Number,SS-3000
Sample ID,00532
Filter ID,A2
Start Time,2023-09-27 10:08:17-07:00
Filter Start Time,2023-09-27 10:11:41-07:00
Duration,0:10:53
Total Volume (l),1.01
Peak Pressure (psi),5.0
Peak Flow (l/min),1.1
Location,45.743593,-122.617424
Elevation (m),255.1
GPS Accuracy (±m),28.5

Elapsed time (s),Volume (l),Pressure (psi),Flow (l/min),Status,Stage
0,0.81,3.37,1.00,,1
2,0.84,3.96,1.00,,1
4,0.88,4.68,1.00,,1
6,0.91,3.59,1.00,,1
8,0.94,4.23,1.01,,1
```

Figure 9.2: Log file example

Each log file contains a header section at the top, followed by a data section (figure 9.2).

9.2.1 Header Section

The header section (figure 9.2) contains general information and statistics that pertain to all samples in the run.

1. Serial Number – The *e*DNA Autosampler unit serial number.
2. Sample ID – The auto-incrementing sample identifier (00000–99999).
3. Filter ID – Manifold location identifier (A1–B4).
4. Start Time – Start time of the flush stage.
5. Filter Start Time – Start time of the sample stage—when the filter element is first exposed to the environment. See section 9.3 THE

AUTOSAMPLING PROCESS (page 32).
6. Target Volume – The TARGET VOLUME setting for this sample.
7. Target Flow – The TARGET FLOW setting for this sample.
8. Flush Volume – The FLUSH VOLUME setting for this sample.
9. Filter Clog Point – The FILTER CLOG POINT setting for this sample.
10. Prime Time – The PRIME TIME setting for this sample.
11. Peak Pressure – Maximum pressure at any stage in -PSI.
12. Avg Flow – Average flow for all stages in l/min.
13. Location – GPS coordinates of the sample location.
14. Elevation – GPS elevation of the sample location in meters.
15. GPS Accuracy – GPS location accuracy in meters.

9.2.2 Data Section

The data section (figure 9.2) contains the profile of sensor data measured during the sample run. A data row is created once every two seconds when the pump is running.

1. Elapsed Time – Elapsed time in seconds since the beginning of the first stage of the sample.
2. Volume – Cumulative filtered volume in liters.
3. Pressure – Instantaneous pressure in -PSI.
4. Flow – Instantaneous flow in l/min.
5. Status – Special condition flags. When flags are present the unit is operating with one of the following special conditions:
   `l` = Low flow
   `m` = Minimum flow (too low to accurately meter)
   `f` = Freeze warning
   `t` = High temperature warning
6. Stage – Numeric sample stage identifier. One of:
   `-1` = Manual Control
   `0` = Off
   `1` = Flush
   `2` = Pre-Drain
   `3` = Sample
   `4` = Dry
   `5` = Post-Drain
   See section 9.3 THE AUTOSAMPLING PROCESS (page 32) for details.

9.3 The Autosampling Process

The sampling process consists of five stages, each represented by a segment in the main screen progress bar. Should any stage fail, that stage is aborted and the process continues at the next stage.

1. The **flush** stage primes the system and flushes a volume of water through the lower manifold. The PRIME TIME setting determines how long to wait for the pump to prime, and the FLUSH VOLUME setting determines the volume of water used.
2. The **pre-drain** stage removes water from the lower manifold. This step is necessary so that the next stage can achieve an accurate volume measurement.
3. The **sample** stage unseals the filter housing, primes the system, and pumps water through the filter membrane. The PRIME TIME setting determines how long to wait for the pump to prime, and the TARGET VOLUME setting determines how much water to pump through the filter membrane. This stage ends 250 ml shy of the target volume to account for water in the filter housing and tubing.
4. The **dry** stage removes water from the filter housing and dries the filter membrane. Water removed at this stage has passed through the filter membrane and is counted toward sample volume. At the end of this stage the filter housing is sealed off until the sample is collected.
5. The **post-drain** stage removes water from the lower manifold to keep the system cleaner while awaiting the next sample. At the end of this stage the unit automatically goes to sleep.

Index

Battery, 1, 7, 20
Beeper, 3, 20

Clock, 7
CSV, 17, 18, 29

Diagnostics, 24

Filter ID, 17
Flow, 8
Flow sensor, 28

GPS, 7, 28

Logs, 14, 29

Manifold ID, 7
Manual control, 26

Power LED, 20
Pressure, 8
Pressure sensor, 28

RTC, 25

Sample ID, 7
Schedule, 11, 15
Software update, 23
Sterilize, 21

USB, 3, 14, 17, 18, 23
UTC, 24

Volume, 7
Volume sensor, 28

Smith-Root
