# Supplementary material for: Automated eDNA sampling for marine monitoring and biosecurity: optimising temporal resolution, remote deployments, and community engagement
Source: PeerJ. 2026 May 28;14:e21287. doi: 10.7717/peerj.21287 (PMC13222548; doi:10.7717/peerj.21287)
Supplement: Supplemental Information 4 [file peerj-14-21287-s004.docx]

**Automated eDNA sampling for marine monitoring and biosecurity: optimising temporal resolution, remote deployments, and community engagement**

*Appendix 3: Feedback report from the local Māori community of Ngāti Manu*

**What value does Ngāti Manu see in the eDNA dataset for the hapū and local marine environment?**

The pace of climate change impacts is accelerating. The frequency and intensity of severe weather events are increasing. Recent severe weather events have brought about profound and lasting changes for whānau, and the landscapes we care for. Today, the Kāretu watershed - its estuaries and tributaries are sensitive to hydrological changes from development and pollutants. The rohe is especially vulnerable to the impacts of water pollution, biodiversity loss and land degradation. Flooding, landslides, rising sea levels and erosion, are posing threats to infrastructure, both public and private properties, marae, kāinga, taonga and sites of cultural importance as well as the local natural environment. How we face these changes together requires knowledge of changes and ongoing planning.

The eDNA dataset provides us with up-to-date knowledge of our marine and freshwater environments. This provides us with a way of tracking changes and builds our knowledge of and relationships with both. By understanding the impacts of climate change on our collective socio-environmental wellbeing, we will be able to better plan for our future.

**Did the tool meet the needs and expectations?**

Not only did the tool provide a wealth of information, but it built capability within our community and our understanding of the Matauranga-Science Interface. In particular, by ensuring our young people are central to this process, the tool has helped us to grow leaders with the skills to take us into the future.

**Did the results provide the information or insights your hapū hoped to gain?**

Yes, we have now built relationships with other hapu so that the knowledge can be widely available. It has also helped us to understand how we can observe changes in the natural environment.

**What improvements or additional features would like to see in this tool?**

It might be good to think about how the device might also support matauranga or embed matauranga more generally in its deployment and collection.

**Overall, how would you describe your experience with the device, the sampling process and the data output?**

excellent
